# Subsequent Event Risk in Individuals With Established Coronary Heart Disease: Design and Rationale of the GENIUS-CHD Consortium

**DOI:** 10.1161/CIRCGEN.119.002470

**Published:** 2019-04-16

**Authors:** Riyaz S. Patel, Vinicius Tragante, Amand F. Schmidt, Raymond O. McCubrey, Michael V. Holmes, Laurence J. Howe, Kenan Direk, Axel Åkerblom, Karin Leander, Salim S. Virani, Karol A. Kaminski, Jochen D. Muehlschlegel, Hooman Allayee, Peter Almgren, Maris Alver, Ekaterina V. Baranova, Hassan Behloui, Bram Boeckx, Peter S. Braund, Lutz P. Breitling, Graciela Delgado, Nubia E. Duarte, Marie-Pierre Dubé, Line Dufresne, Niclas Eriksson, Luisa Foco, Markus Scholz, Crystel M. Gijsberts, Charlotte Glinge, Yan Gong, Jaana Hartiala, Mahyar Heydarpour, Jaroslav A. Hubacek, Marcus Kleber, Daniel Kofink, Salma Kotti, Pekka Kuukasjärvi, Vei-Vei Lee, Andreas Leiherer, Petra A. Lenzini, Daniel Levin, Leo-Pekka Lyytikäinen, Nicola Martinelli, Ute Mons, Christopher P. Nelson, Kjell Nikus, Anna P. Pilbrow, Rafal Ploski, Yan V. Sun, Michael W.T. Tanck, W.H.Wilson Tang, Stella Trompet, Sander W. van der Laan, Jessica Van Setten, Ragnar O. Vilmundarson, Chiara Viviani Anselmi, Efthymia Vlachopoulou, Lawien Al Ali, Eric Boerwinkle, Carlo Briguori, John F. Carlquist, Kathryn F. Carruthers, Gavino Casu, John Deanfield, Panos Deloukas, Frank Dudbridge, Thomas Engstrøm, Natalie Fitzpatrick, Kim Fox, Bruna Gigante, Stefan James, Marja-Liisa Lokki, Paulo A. Lotufo, Nicola Marziliano, Ify R. Mordi, Joseph B. Muhlestein, Christopher Newton-Cheh, Jan Pitha, Christoph H. Saely, Ayman Samman-Tahhan, Pratik B. Sandesara, Andrej Teren, Adam Timmis, Frans Van de Werf, Els Wauters, Arthur A.M. Wilde, Ian Ford, David J. Stott, Ale Algra, Maria G. Andreassi, Diego Ardissino, Benoit J. Arsenault, Christie M. Ballantyne, Thomas O. Bergmeijer, Connie R. Bezzina, Simon C. Body, Eric H. Boersma, Peter Bogaty, Michiel L. Bots, Hermann Brenner, Jasper J. Brugts, Ralph Burkhardt, Clara Carpeggiani, Gianluigi Condorelli, Rhonda M. Cooper-DeHoff, Sharon Cresci, Nicolas Danchin, Ulf de Faire, Robert N. Doughty, Heinz Drexel, James C. Engert, Keith A.A. Fox, Domenico Girelli, Diederick E. Grobbee, Emil Hagström, Stanley L. Hazen, Claes Held, Harry Hemingway, Imo E. Hoefer, G. Kees Hovingh, Reza Jabbari, Julie A. Johnson, J. Wouter Jukema, Marcin P. Kaczor, Mika Kähönen, Jiri Kettner, Marek Kiliszek, Olaf H. Klungel, Bo Lagerqvist, Diether Lambrechts, Jari O. Laurikka, Terho Lehtimäki, Daniel Lindholm, B. K. Mahmoodi, Anke H. Maitland-van der Zee, Ruth McPherson, Olle Melander, Andres Metspalu, Anna Niemcunowicz-Janica, Oliviero Olivieri, Grzegorz Opolski, Colin N. Palmer, Gerard Pasterkamp, Carl J. Pepine, Alexandre C. Pereira, Louise Pilote, Arshed A. Quyyumi, A. Mark Richards, Marek Sanak, Agneta Siegbahn, Tabassome Simon, Juha Sinisalo, J. Gustav Smith, John A. Spertus, Steen Stender, Alexandre F.R. Stewart, Wojciech Szczeklik, Anna Szpakowicz, Jean-Claude Tardif, Jurriën M. ten Berg, Jacob Tfelt-Hansen, George Thanassoulis, Joachim Thiery, Christian Torp-Pedersen, Yolanda van der Graaf, Frank L.J. Visseren, Johannes Waltenberger, Peter E. Weeke, Pim Van der Harst, Chim C. Lang, Naveed Sattar, Vicky A. Cameron, Jeffrey L. Anderson, James M. Brophy, Guillaume Pare, Benjamin D. Horne, Winfried März, Lars Wallentin, Nilesh J. Samani, Aroon D. Hingorani, Folkert W. Asselbergs

**Affiliations:** 1 Institute of Cardiovascular Science (R.S.P., A.F.S., L.J.H., K.D., J.D., A.D.H., F.W.A), Faculty of Population Health Science, University College London, United Kingdom.; 2 Institute of Health Informatics (N.F., A. Timmis, H.H., F.W.A.), Faculty of Population Health Science, University College London, United Kingdom.; 3 Bart’s Heart Centre, St Bartholomew’s Hospital, London (R.S.P., J.D., A. Timmis).; 4 Division of Heart and Lungs, Department of Cardiology (V.T., A.F.S.,D.K.,F.W.A.), UMC Utrecht, the Netherlands.; 5 Laboratory of Experimental Cardiology (C.M.G.), UMC Utrecht, the Netherlands.; 6 Department of Clinical Chemistry and Hematology (I.E.H.), UMC Utrecht, the Netherlands.; 7 Department of Clinical Chemistry (G.P.), UMC Utrecht, the Netherlands.; 8 Intermountain Heart Institute, Intermountain Medical Center, Salt Lake City, UT (R.O.M., J.F.C., J.B.M., J.L.A., B.D.H).; 9 Clinical Trial Service Unit and Epidemiological Studies Unit, Nuffield Department of Population Health, Medical Research Council Population Health Research Unit, University of Oxford, United Kingdom (M.V.H).; 10 National Institute for Health Research Oxford Biomedical Research Centre, Oxford University Hospital, United Kingdom (M.V.H.).; 11 Uppsala Clinical Research Center, Sweden (A. Åkerblom, N.E., S.J., C.H., B.L., D. Lindholm, A. Siegbahn, L.W.).; 12 Division of Cardiology, Department of Medical Sciences (A. Åkerblom, C.H., D. Lindholm, S.J., B.L., L.W.), Uppsala University, Sweden.; 13 Division of Clinical Chemistry, Department of Medical Sciences (A. Siegbahn), Uppsala University, Sweden.; 14 Institute of Environmental Medicine, Karolinska Institute, Stockholm, Sweden (K.L., B.G., U.d.F.).; 15 Section of Cardiology, Michael E. DeBakey Veterans Affairs Medical Center, Houston, TX (S.S.V.).; 16 Section of Cardiovascular Research, Department of Medicine, Baylor College of Medicine, Houston, TX (S.S.V., C.M.B.).; 17 Department of Population Medicine and Civilization Disease Prevention (K.A.K.), Medical University of Bialystok, Poland.; 18 Department of Cardiology (K.A.K., A. Szpakowicz), Medical University of Bialystok, Poland.; 19 Department of Anesthesiology, Perioperative and Pain Medicine, Brigham and Women’s Hospital, Boston, MA (J.D.M., M.H.).; 20 Harvard Medical School, Boston, MA (J.D.M., M.H., S.C.B).; 21 Departments of Preventive Medicine and Biochemistry and Molecular Medicine (H.A., J.H.), Keck School of Medicine of USC, Los Angeles, CA.; 22 Institute for Genetic Medicine (J.H.), Keck School of Medicine of USC, Los Angeles, CA.; 23 Department of Clinical Sciences, Lund University, Malmö, Sweden (P.A., O.M.).; 24 Estonian Genome Centre, Department of Biotechnology, Institute of Genomics, Institute of Molecular and Cell Biology, University of Tartu, Estonia (M.A., A.M.).; 25 Division of Pharmacoepidemiology and Clinical Pharmacology, Utrecht University, the Netherlands (E.V.B., O.H.K., A.H.M.-v.d.Z.).; 26 Centre for Outcomes Research and Evaluation, Research Institute of the McGill University Health Centre, Montreal, QC, Canada (H.B., L.D., L.P., G.T., J.M.B.).; 27 Research Institute of the McGill University Health Centre, Montreal, QC, Canada (J.C.E.).; 28 Laboratory for Translational Genetics, Department of Human Genetics (B.B., D. Lambrechts), Katholieke Universiteit Leuven, Belgium.; 29 Department of Cardiovascular Sciences (F.V.d.W.), Katholieke Universiteit Leuven, Belgium.; 30 Laboratory for Translational Genetics, VIB Center for Cancer Biology, Belgium (B.B., D. Lambrechts).; 31 Department of Cardiovascular Sciences, BHF Cardiovascular Research Centre, University of Leicester, United Kingdom (P.S.B., C.P.N., N.J.S.).; 32 NIHR Leicester Biomedical Research Centre, Glenfield Hospital, United Kingdom (P.S.B., C.P.N., N.J.S.).; 33 Division of Clinical Epidemiology and Aging Research, German Cancer Research Center (DKFZ), Heidelberg (L.P.B., U.M.).; 34 Fifth Department of Medicine, Medical Faculty Mannheim, Heidelberg University, Germany (G.D., M. Kleber, W.M.).; 35 Heart Institute, University of Sao Paulo, Brazil (N.E.D., A.C.P.).; 36 Montreal Heart Institute, OC, Canada (M.-P.D., J.-C.T.).; 37 Faculty of Medicine, Université de Montréal, QC, Canada (M.-P.D., J.-C.T.).; 38 Preventive and Genomic Cardiology, McGill University Health Centre, Montreal, QC, Canada (L.D., J.C.E., G.T.).; 39 Institute for Biomedicine, Eurac Research, Affiliated Institute of the University of Lübeck, Bolzano, Italy (L.F.).; 40 Institute for Medical Informatics, Statistics, and Epidemiology (M.S.), University of Leipzig, Germany.; 41 LIFE Research Centre for Civilization Diseases (M.S., A. Teren, R.B., J.T.), University of Leipzig, Germany.; 42 Department of Cardiology, The Heart Centre, Copenhagen University Hospital, Rigshospitalet (C.G., T.E., R.J.).; 43 Amsterdam UMC, University of Amsterdam, Clinical and Experimental Cardiology, Amsterdam Cardiovascular Sciences, AMC Heart Center, the Netherlands (C.G., A.A.M.W., C.R.B.).; 44 Department of Pharmacotherapy and Translational Research, Centre for Pharmacogenomics (Y.G., R.M.C.-D., J.A.J.), University of Florida, Gainesville.; 45 Division of Cardiovascular Medicine, College of Medicine (R.M.C.-D., J.A.J., C.J.P.), University of Florida, Gainesville.; 46 Centre for Experimental Medicine, Institute for Clinical and Experimental Medicine, Prague, Czech Republic (J.A.H., J.P.).; 47 Assistance Publique-Hôpitaux de Paris (AP-HP), Department of Clinical Pharmacology, Platform of Clinical Research of East Paris (URCEST-CRCEST-CRB HUEP-UPMC), France (S.K.).; 48 Department of Cardio-Thoracic Surgery (P.K.), University of Tampere, Finland.; 49 Department of Clinical Chemistry (L.-P.L., T.L.), University of Tampere, Finland.; 50 Department of Cardiology (K.N.), University of Tampere, Finland.; 51 Department of Clinical Physiology (M. Kähönen), University of Tampere, Finland.; 52 Department of Cardio-Thoracic Surgery, Finnish Cardiovascular Research Center, Faculty of Medicine & Life Sciences (J.O.L.), University of Tampere, Finland.; 53 Department of Biostatistics and Epidemiology, Texas Heart Institute, Houston (V.-V.L.).; 54 Vorarlberg Institute for Vascular Investigation and Treatment (VIVIT), Feldkirch, Austria (A. Leiherer, C.H.S., H.D.).; 55 Private University of the Principality of Liechtenstein, Triesen (A. Leiherer, C.H.S., H.D.).; 56 Medical Central Laboratories, Feldkirch, Austria (A. Leiherer).; 57 Statistical Genomics Division, Department of Genetics (P.A. Lenzini, S.C.), Washington University School of Medicine, Saint Louis, MO.; 58 Cardiovascular Division, Department of Medicine (S.C.), Washington University School of Medicine, Saint Louis, MO.; 59 Division of Molecular and Clinical Medicine, School of Medicine, University of Dundee, Scotland, United Kingdom (D. Levin, I.R.M., C.C.L.).; 60 Department of Clinical Chemistry, Fimlab Laboratories, Tampere, Finland (L.-P.L., T.L.).; 61 Department of Medicine, University of Verona, Italy (N.M., D.G., O.O.).; 62 Department of Cardiology, Heart Center (K.N.), Tampere University Hospital, Finland.; 63 Department of Clinical Physiology (M. Kähönen), Tampere University Hospital, Finland.; 64 Department of Cardio-Thoracic Surgery, Heart Centre (J.O.L.), Tampere University Hospital, Finland.; 65 The Christchurch Heart Institute, University of Otago Christchurch, New Zealand (A.P.P., A.M.R., V.A.C.).; 66 Department of Medical Genetics (R. Ploski), Medical University of Warsaw, Poland.; 67 first Chair and Department of Cardiology (G.O.), Medical University of Warsaw, Poland.; 68 Department of Epidemiology, Emory University Rollins School of Public Health, Atlanta, GA (Y.V.S.).; 69 Amsterdam UMC, University of Amsterdam, Clinical Epidemiology and Biostatistics, The Netherlands (M.W.T.T.).; 70 Department of Biomedical Informatics (Y.V.S.), Emory Clinical Cardiovascular Research Institute, Emory University School of Medicine, Atlanta, GA.; 71 Division of Cardiology, Department of Medicine (A.S.-T., P.B.S., A.A.Q.), Emory Clinical Cardiovascular Research Institute, Emory University School of Medicine, Atlanta, GA.; 72 Department of Cellular and Molecular Medicine, Lerner Research Institute, Cleveland Clinic, OH (W.H.W.T., S.L.H.).; 73 Department of Cardiovascular Medicine, Heart and Vascular Institute, and Centre for Clinical Genomics, Cleveland Clinic, OH (W.H.W.T.).; 74 Department of Cardiovascular Medicine, Centre for Microbiome and Human Health, Heart and Vascular Institute, Cleveland Clinic, OH (S.L.H.).; 75 Section of Gerontology and Geriatrics, Department of Internal Medicine (S.T.), Leiden University Medical Centre, the Netherlands.; 76 Department of Cardiology (S.T., J.W.J.), Leiden University Medical Centre, the Netherlands.; 77 Division Heart and Lungs, Department of Cardiology, UMC Utrecht, University of Utrecht, the Netherlands (J.V.S.).; 78 Ruddy Canadian Cardiovascular Genetics Centre, University of Ottawa Heart Institute, ON, Canada (R.O.V., R.M., A.F.R.S.).; 79 Department of Biochemistry, Microbiology and Immunology (R.O.V., A.F.R.S.), University of Ottawa, ON, Canada.; 80 Departments of Medicine and Biochemistry, Microbiology and Immunology(R.M.), University of Ottawa, ON, Canada.; 81 Department of Cardiovascular Medicine, Humanitas Clinical and Research Centre, Milan, Italy (C.V.A., G.C.).; 82 Transplantation Laboratory (E.V., M.-L.L.), Helsinki University Hospital and University of Helsinki, Finland.; 83 Heart and Lung Centre (J.S.), Helsinki University Hospital and University of Helsinki, Finland.; 84 University Medical Centre, University of Groningen, the Netherlands (L.A.A., P.V.d.H.).; 85 University of Texas School of Public Health, Houston(E.B.).; 86 Clinica Mediterranea, Naples, Italy (C.B.).; 87 Cardiology Division, Department of Internal Medicine (J.F.C., J.B.M., J.L.A.), University of Utah, Salt Lake City.; 88 Department of Biomedical Informatics (B.D.H.), University of Utah, Salt Lake City.; 89 QMRI, Cardiovascular Sciences, University of Edinburgh, United Kingdom (K.F.C.).; 90 The University of Edinburgh, United Kingdom (K.A.A.F).; 91 ATS Sardegna, ASSL Nuoro—Ospedale San Francesco, Nuoro, Italy (G.C.).; 92 William Harvey Research Institute, Barts and the London Medical School (P.D), Queen Mary University of London, United Kingdom.; 93 Centre for Genomic Health (P.D.), Queen Mary University of London, United Kingdom.; 94 Department of Health Sciences, University of Leicester, United Kingdom (F.D.).; 95 Department of Cardiology, University of Lund, Sweden (T.E.).; 96 National Heart and Lung Institute, Imperial College and Institute of Cardiovascular Medicine and Science, Royal Brompton Hospital, London, United Kingdom (K.F.); 97 Centro de Pesquisa Clinica, Hospital Universitario, Universidade de Sao Paulo, São Paulo, Brazil (P.A. Lotufo, ).; 98 ATS Sardegna, ASL 3 Nuoro, Nuoro, Italy (N. Marziliano).; 99 Cardiovascular Research Center, Center for Human Genetic Research, Massachusetts General Hospital, Boston (C.N.-C.).; 100 Program in Medical and Population Genetics, Broad Institute, Cambridge, MA (C.N.-C.).; 101 Department of Medicine and Cardiology, Academic Teaching Hospital Feldkirch, Austria (C.H.S.).; 102 Heart Centre Leipzig, Germany (A. Teren).; 103 Respiratory Oncology Unit, Department of Respiratory Medicine, University Hospitals KU Leuven, Belgium (E.W.).; 104 Princess Al-Jawhara Al-Brahim Centre of Excellence in Research of Hereditary Disorders, Jeddah, Saudi Arabia (A.A.M.W.).; 105 Robertson Centre for Biostatistics, University of Glasgow, United Kingdom (I.F.).; 106 Institute of Cardiovascular and Medical Sciences, University of Glasgow, United Kingdom (D.J.S., N.S.).; 107 Division Laboratories, Pharmacy, and Biomedical Genetics, Laboratory of Clinical Chemistry and Hematology (S.W.v.d.L.), UMC Utrecht, Utrecht University, the Netherlands.; 108 Department of Neurology and Neurosurgery, Brain Centre Rudolf Magnus and Julius Centre for Health Sciences and Primary Care (A. Algra), UMC Utrecht, Utrecht University, the Netherlands.; 109 Julius Center for Health Sciences and Primary Care (M.B., D.E.G., Y.v.d.G.), UMC Utrecht, Utrecht University, the Netherlands.; 110 Department of Vascular Medicine (F.L.J.V), UMC Utrecht, Utrecht University, the Netherlands.; 111 CNR Institute of Clinical Physiology, Pisa (M.G.A, C.C).; 112 Cardiology Department, Parma University Hospital, Italy (D.A.).; 113 Centre de recherche de l’Institut Universitaire de cardiologie et de pneumologie de Québec, Canada (B.J.A.).; 114 Department of Medicine, Faculty of Medicine, Université Laval, QC, Canada (B.J.A.); 115 St Antonius Hospital, Department Cardiology, Nieuwegein, the Netherlands (T.O.B., B.K.M., J.M.t.B.).; 116 Department of Anesthesia, Critical Care & Pain Medicine, Beth Israel Deaconess Medical Center, Boston, MA (S.B.).; 117 Department of Cardiology, Erasmus MC, Thoraxcenter (E.H.B., J.J.B.).; 118 Cardiovascular Research School, Erasmus Medical Center (COEUR), Rotterdam, the Netherlands(E.H.B.).; 119 Laval University, Institute universitaire de cardiologie et de pneumologie de Québec, Canada (P.B.).; 120 Network Aging Research (NAR), University of Heidelberg (H.B.).; 121 Institute of Clinical Chemistry and Laboratory Medicine, University Hospital Regensburg, Germany (R.B.).; 122 Department of Biomedical Sciences, Humanitas University, Milan, Italy (G.C.).; 123 Assistance Publique-Hôpitaux de Paris (AP-HP), Department of Cardiology, Hôpital Européen Georges Pompidou & FACT (French Alliance For Cardiovascular Trials), Université Paris Descartes, France (N.D.).; 124 Université Paris-Descartes, France (N.D.).; 125 Heart Health Research Group, University of Auckland, New Zealand (R.N.D.).; 126 Drexel University College of Medicine, Philadelphia PA (H.D.).; 127 Division of Cardiology, Department of Medicine, Royal Victoria Hospital, McGill Univ Health Centre, Montreal, QC, Canada (J.C.E., G.T.).; 128 Department of Cardiology, Uppsala Clinical Research Centre, Uppsala University, Sweden (E.H.).; 129 Department of Vascular Medicine, Academic Medical Centre, Amsterdam (G.K.H.).; 130 Einthoven Laboratory for Experimental Vascular Medicine, LUMC, Leiden (J.W.J.).; 131 Interuniversity Cardiology Institute of the Netherlands, Utrecht (J.W.J.).; 132 Department of Internal Medicine, Jagiellonian University Medical College, Kraków, Poland (M.P.K., M.S., W.S).; 133 Cardiology Centre, Institute for Clinical and Experimental Medicine, Prague, Czech Republic (J.K.).; 134 Department of Cardiology and Internal Diseases, Military Institute of Medicine, Warsaw, Poland (M. Kiliszek).; 135 Department of Respiratory Medicine, Academic Medical Centre, University of Amsterdam (A.H.M.-v.d.Z.).; 136 Department of Internal Medicine, Skåne University Hospital, Malmö, Sweden (O.M.).; 137 Department of Forensic Medicine; Medical University of Bialystok (A.N.-J.).; 138 Pat Macpherson Centre for Pharmacogenetics and Pharmacogenomics, Division of Molecular and Clinical Medicine, Ninewells Hospital and Medical School, Dundee (C.N.P.).; 139 Department of Medicine, McGill University Health Centre, Montreal, QC, Canada (L.P., J.M.B.).; 140 Cardiovascular Research Institute, National University of Singapore (A.M.R.).; 141 Assistance Publique-Hôpitaux de Paris (AP-HP), Department of Clinical Pharmacology, Platform of Clinical Research of East Paris (URCEST-CRCEST-CRB HUEP-UPMC), FACT (French Alliance for Cardiovascular Trials), Sorbonne Université (T.S.).; 142 Paris-Sorbonne University, UPMC-Site St Antoine, France (T.S.).; 143 Department of Cardiology, Clinical Sciences, Lund University, Skåne University Hospital (J.G.S.).; 144 Wallenberg Centre for Molecular Medicine, Lund University Diabetes Centre, Lund University, Sweden (J.G.S.).; 145 Program in Medical and Population Genetics, Broad Institute, Cambridge, MA (J.G.S.).; 146 Saint Luke’s Mid America Heart Institute, University of Missouri-Kansas City (J.A.S.).; 147 Saint Luke’s Mid America Heart Insti Kansas City, MO (J.A.S.).; 148 Department of Clinical Biochemistry, Copenhagen University Hospital, Gentofte (S.S.).; 149 Department of Cardiology, The Heart Centre, Copenhagen University Hospital, Rigshospitalet (J.T.-H.).; 150 Department of Forensic Medicine, Faculty of Medical Sciences, University of Copenhagen, Denmark (J.T.-Hansen).; 151 Institute of Laboratory Medicine, Clinical Chemistry and Molecular Diagnostics, University Hospital, Leipzig, Germany (J.T.).; 152 Unit of Epidemiology and Biostatistics, Department of Health Science and Technology, Aalborg University Hospital, Denmark (C.T.-Pedersen).; 153 Department of Cardiovascular Medicine, University of Münster, Germany (J.W.).; 154 Department of Cardiology, Herlev and Gentofte Hospital, Hellerup, Denmark (P.E.W.).; 155 Department of Pathology and Molecular Medicine, McMaster University (G.P.).; 156 Population Health Research Institute, Hamilton, ON, Canada (G.P.).; 157 Synlab Academy, Synlab Holding Deutschland GmbH, Mannheim, Germany (W.M.).; 158 Clinical Institute of Medical and Chemical Laboratory Diagnostics, Medical University of Graz, Austria (W.M.).; 159 Durrer Centre of Cardiogenetic Research, ICIN-Netherlands Heart Institute, Utrecht (F.W.A.).

**Keywords:** coronary artery disease, genetics, myocardial infarction, prognosis, secondary prevention

## Abstract

**Background:**

The Genetics of Subsequent Coronary Heart Disease (GENIUS-CHD) consortium was established to facilitate discovery and validation of genetic variants and biomarkers for risk of subsequent CHD events, in individuals with established CHD.

**Methods:**

The consortium currently includes 57 studies from 18 countries, recruiting 185 614 participants with either acute coronary syndrome, stable CHD, or a mixture of both at baseline. All studies collected biological samples and followed-up study participants prospectively for subsequent events.

**Results:**

Enrollment into the individual studies took place between 1985 to present day with a duration of follow-up ranging from 9 months to 15 years. Within each study, participants with CHD are predominantly of self-reported European descent (38%–100%), mostly male (44%–91%) with mean ages at recruitment ranging from 40 to 75 years. Initial feasibility analyses, using a federated analysis approach, yielded expected associations between age (hazard ratio, 1.15; 95% CI, 1.14–1.16) per 5-year increase, male sex (hazard ratio, 1.17; 95% CI, 1.13–1.21) and smoking (hazard ratio, 1.43; 95% CI, 1.35–1.51) with risk of subsequent CHD death or myocardial infarction and differing associations with other individual and composite cardiovascular endpoints.

**Conclusions:**

GENIUS-CHD is a global collaboration seeking to elucidate genetic and nongenetic determinants of subsequent event risk in individuals with established CHD, to improve residual risk prediction and identify novel drug targets for secondary prevention. Initial analyses demonstrate the feasibility and reliability of a federated analysis approach. The consortium now plans to initiate and test novel hypotheses as well as supporting replication and validation analyses for other investigators.

## Abstract 2

Supplemental Digital Content is available in the text.

Major public health initiatives and policy changes, along with advances in drug and interventional therapies have significantly reduced cardiovascular morbidity and mortality in most high-income countries.^1–3^ However, the improved survival rates following an initial presentation with coronary heart disease (CHD) has, paradoxically, led to a growing number of patients living with established CHD (eg, 16M in the United States and 3M in the United Kingdom)^4,5^ who remain at substantially high risk of subsequent cardiovascular events. These include myocardial infarction (MI), repeated revascularizations but also heart failure, stroke, and sudden death.^4^

Despite a large body of knowledge on the pathophysiology of first CHD events in general populations,^6,7^ little is known about factors that influence disease progression or subsequent events in patients with established CHD, beyond those consequent to the acute index event in the short-term (such as biomarkers of myocardial dysfunction or necrosis, left ventricular function, or arrhythmia).^8^ As a result, although guidelines and treatment thresholds have progressively evolved over the past 2 decades, the targeted risk factors per se have remained largely unaltered.^9^ Novel therapies beyond lipid lowering, antiplatelet agents, and drugs recommended for high blood pressure and heart failure have been slow to emerge. Importantly, multiple novel and existing agents (eg, darapladib, varespladib, and folic acid) have failed in very late stage clinical development despite promising observational data.^10–13^ In contrast, some traditional risk factors, such as obesity, which show robust associations with initial CHD onset,^14^ continue to show inverse or null associations with subsequent events once CHD has developed.^15^

Ultimately, the high (residual) risk in individuals with existing CHD despite optimal contemporary therapy emphasizes the need for studying risk of subsequent events and their related causal pathways. For example, in the intervention arm of the IMPROVE-IT study (Vytorin Efficacy International Trial), despite simvastatin and ezetimibe treatment following an acute coronary syndrome, at 7 years, almost a third of participants experienced the primary end point (a composite of cardiovascular death, major coronary event, coronary revascularization, or nonfatal stroke).^16^ Similarly, in the FOURIER trial (Further Cardiovascular Outcomes Research with PCSK9 [proprotein convertase subtilisin-kexin type 9] Inhibition in Subjects with Elevated Risk), almost 10% of patients with established but stable CVD, experienced an event at 2.2 years despite high-intensity statin and PCSK9 inhibition, with achieved median LDL-C (low-density lipoprotein cholesterol) levels of 30 mg/dL.^17^ These data point to the existence of risk factors beyond traditional ones such as LDL-C, and the need to elucidate their related causal pathways.^18^ By studying those with established CHD at high risk of subsequent events, we plan to gain novel insights into other drivers of atherosclerosis or features that identify patients who may benefit most from novel therapies.^9^ Genetic and biomarker studies in these individuals may help identify novel molecular pathways and future drug targets with the goal of advancing precision medicine.

In the absence of a single-large resource to study the determinants of coronary heart disease prognosis, we have established the Genetics of Subsequent CHD (GENIUS-CHD) consortium.^19^ Assembling studies from across the globe that have recruited patients with different types of CHD at baseline, have acquired prospective follow-up, and have stored biological specimens, or genetic data, the consortium aims to: (1) investigate genetic and nongenetic determinants of risk for subsequent CHD, systematically and at scale and (2) facilitate access to data and expertise, as a platform to foster collaboration among investigators working in the field.

Here, we describe the design of the consortium, including details of participating studies, available data, and samples, as well as the governance procedures and the consortium’s approach to data sharing and collaboration to further advance the stated scientific aims. In addition, we present some early findings from an investigation of the association of patient characteristics and certain routinely recorded measures on the risk of subsequent events among patients with different types of CHD at baseline.

## Methods

In accordance with Transparency and Openness Promotion Guidelines, the data, analytic methods, and study materials will be made available to other researchers for purposes of reproducing the results or replicating the procedures. Participating studies received local institutional review board approval and included patients who had provided informed consent at the time of enrollment. The central analysis sites also received waivers from their local institutional review board for collating and analyzing summary-level data from these individual studies. Full details on the eligibility criteria, definitions of terminology, management of the consortium, and planned projects are provided in Materials in the Data Supplement.

## Results

**Figure 1.**
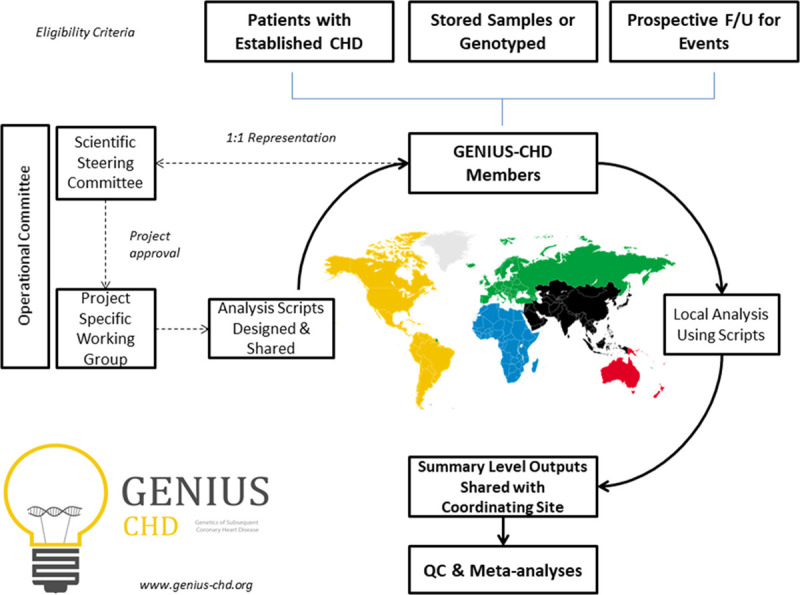
**Overview of the Genetics of Subsequent Coronary Heart Disease (GENIUS-CHD) consortium, illustrating inclusion criteria and governance structure.** Following project approval by the steering committee, analyses scripts are prepared and distributed to all members, with sharing of summary-level outputs before meta-analysis at the coordinating centers. Further details can be found at www.genius-chd.org. QC indicates quality control.

The design and structure of the GENIUS-CHD consortium are presented in Figure 1. Studies meeting the main eligibility criteria were identified and invited to participate (Methods in the Data Supplement). In brief, studies are eligible to join the GENIUS-CHD consortium if they meet 3 inclusion criteria: (1) included individuals with established CHD (defined as the presence of or confirmed history of acute coronary syndrome at baseline, or of coronary artery disease as evidenced by any revascularization procedure (percutaneous coronary intervention or bypass surgery) or demonstrable plaque in any epicardial vessel on direct coronary imaging); (2) acquired prospective follow-up of participants with ascertainment of one or more subsequent cardiovascular disease events as well as all-cause mortality; and (3) had stored blood samples, which are viable and suitable for DNA and biomarker analysis or previously collected such data before sample depletion.

At the time of writing, 57 studies from 18 countries are participating in the consortium and are listed in Table 1. Please refer to www.genius-chd.org for an updated list. Brief narrative descriptions of each study are provided in Methods in the Data Supplement.

**Table 1.**
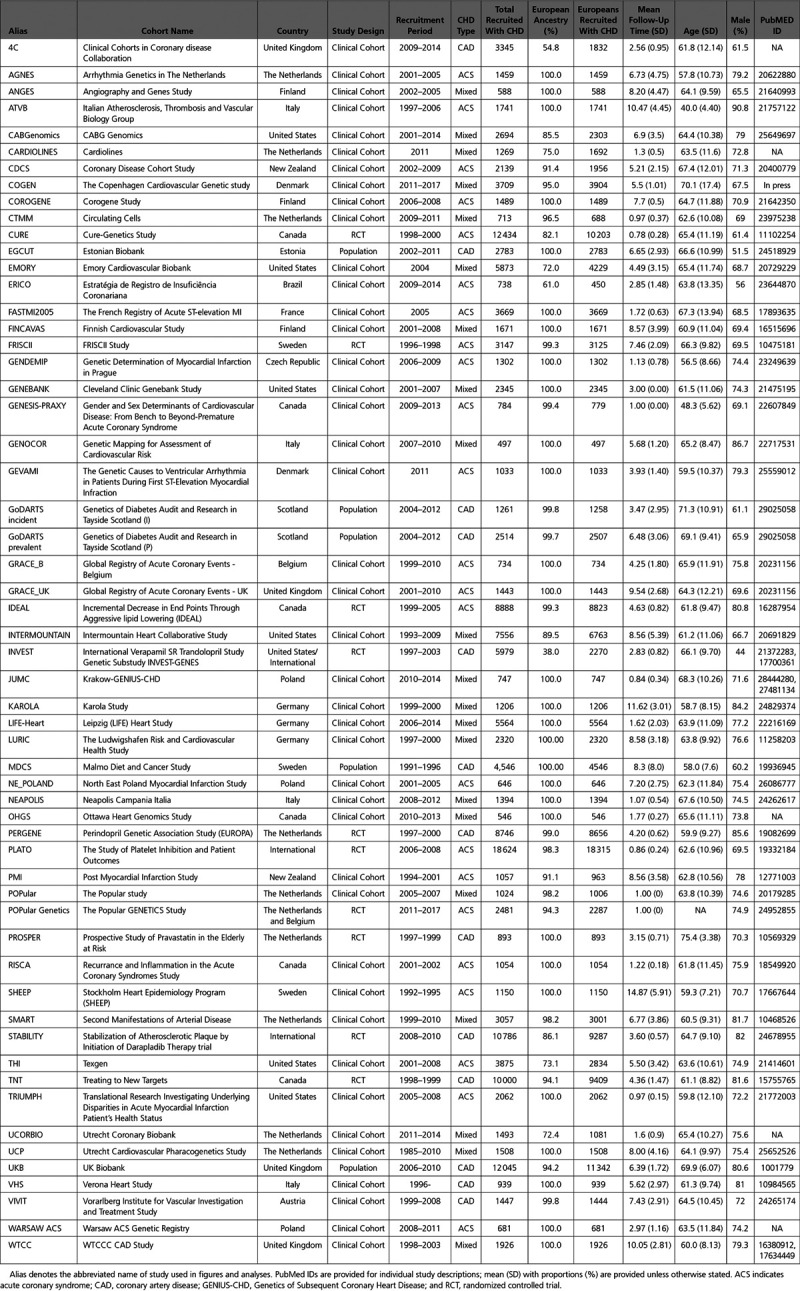
Overview of Each Study Participating in the GENIUS-CHD Consortium

The majority of studies are either investigator-led clinical cohorts (n=42), but clinical trials (n=10) and nested case-cohort (inception-study design) studies (n=5) are also included. Of the total, 23 studies have included participants at the time of an acute coronary syndrome, while the remainder recruited those with stable CHD or a mixture of the 2 (eg, from cardiac catheterization labs). Collectively, 185 614 participants have been enrolled with CHD at baseline (including 812 803 person-years of follow-up); of which 170 343 are of self-reported European descent. Recruitment times varied between studies, ranging from the earliest recruitment in 1985 to studies that remain actively recruiting to the present day. All studies enrolled patients >18 years of age, although one study exclusively recruited only those with premature CHD (MI <45 years), while another recruited only older subjects (>70 years). The overall mean age within each study reflects this heterogeneity, ranging from 40 to 75 years of age, and proportion of male sex ranging from 44% to 91% (Table 1).

### Available Data

#### Core Phenotypes

All studies collected data on age, sex, and ethnicity. Risk factor data are available for diabetes mellitus, obesity, and smoking status in almost all participating studies (96%), while data on concentrations of routine blood lipids (total cholesterol, LDL-C, HDL-C [high-density lipoprotein cholesterol], and triglycerides; 84%), and blood pressure values at enrollment (82%) were collected by the majority of studies. Data on statin use at baseline are available in 90% of all participating studies (Table 2).

**Table 2.**
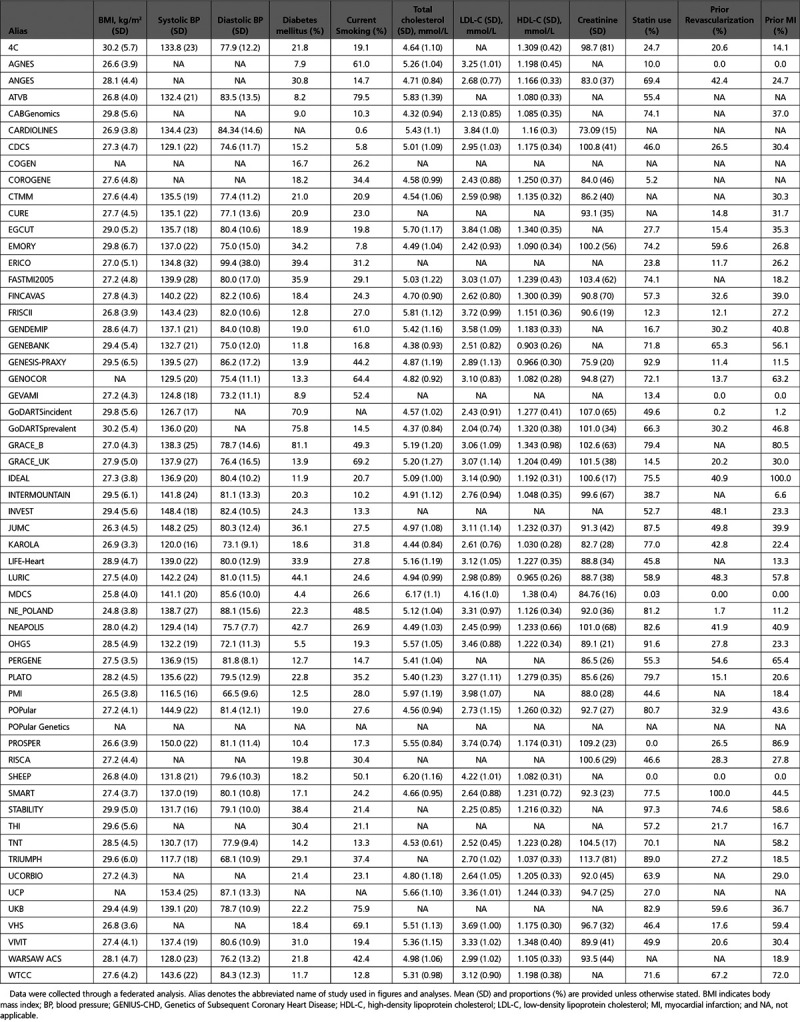
Participant Characteristics of Each Study Contributing to GENIUS-CHD

#### Additional Phenotypes

A list of selected additional phenotypes available by study is presented in Table I in the Data Supplement. Of note, 79% have available data on plasma CRP (C-reactive protein), while coronary disease burden information, from invasive angiography is available in 52% of studies. Finally, over a third of studies have also collected data on physical activity (38%) and socioeconomic status (37%).

#### Samples

Stored samples are available in most studies for future assay testing and stored frozen. The majority have stored plasma (75%), while others also have serum, blood EDTA, RNA, and urine (Table II in the Data Supplement).

#### DNA and Genotyping

More than two-thirds of the studies have DNA still available, either preextracted or as whole blood collected in EDTA and stored for future genotyping. All studies within the consortium have performed genotyping in some capacity, with genome-wide data available in a subset of studies (Table III in the Data Supplement).

#### Subsequent Events and Follow-Up

The most commonly collected end point was all-cause death, collected by all but 2 studies. CHD death during follow-up was collected in 70% of studies, while incident MI was reported by 82% of studies. Studies ascertained end points through different means, including telephone contact, in-person patient interviews, clinical chart reviews, and linkage to national mortality registers and hospital records (Table IV in the Data Supplement).

### Power Calculations

**Figure 2.**
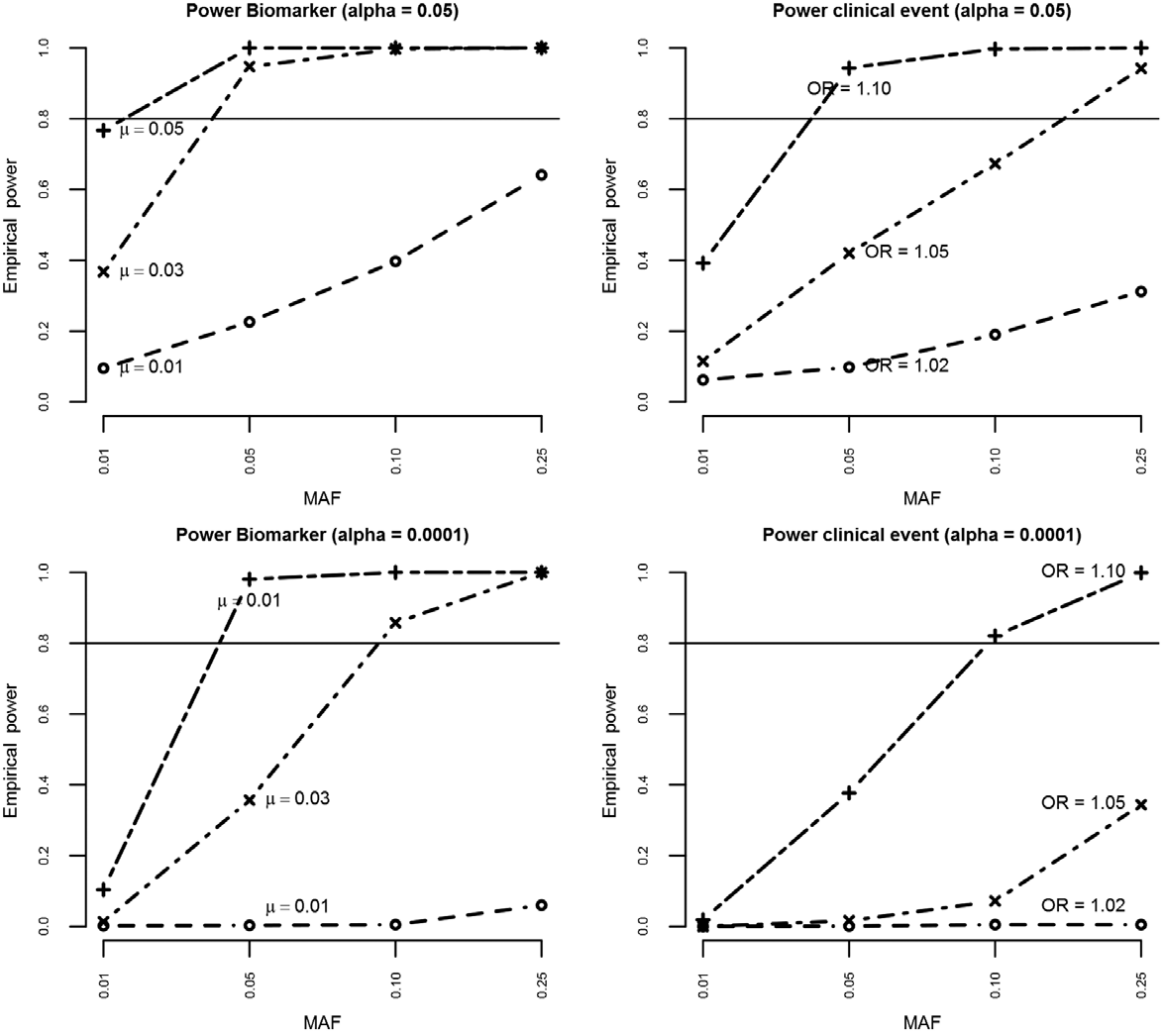
**Figure illustrating empirical power for detecting different effect sizes for biomarker variance and clinical events for both α 0.05 and 0.0001, by varying minor allele frequencies, for a conservative total number of 150 000 with an event rate of 10%.** MAF indicates minor allele frequency; and OR, odds ratio.

Empirical power was estimated based on a conservative sample size of 150 000 subjects with an event rate of 10% (across the entire follow-up period with a mean of about 5 years); Figure 2. Given that the GENIUS-CHD consortium is designed to answer multiple questions, power was estimated for a range of genetic single nucleotide polymorphisms (SNPs) and nongenetic (biomarkers and clinical risk factors) effects.

Minor allele frequencies of 0.01, 0.05, 0.10, and 0.25 were examined, representing rare to common SNPs. For each minor allele frequency, power was calculated for a range of plausible SNP effects on biomarkers (mean difference [μ] 0.01, 0.03, and 0.05) and clinical end points (odds ratios of 1.02, 1.05, and 1.10). For the association of SNPs with biomarkers, power was 80% (α=0.05) or more unless the SNP was rare (minor allele frequency of 0.01) or the effect size was small (eg, 0.01 per allele). For the association of SNPs with clinical end points, power was close to 80% when the effect size was large (odds ratio ≥1.10) or the minor allele frequency was ≥0.10.

Power of observational (ie, nongenetic) analysis was >99% for both continuous and binary exposures unless the odds ratio was close to 1. In addition to continuous and binary outcome data, GENIUS-CHD also collects time-to-event data. Given the similarity (in most empirical settings) between odds ratio and hazard ratios,^20^ similar power is to be expected for time-to-event analysis.

### Initial Analysis

To examine the feasibility of the federated analysis approach, we sought to collect data on participant characteristics, cardiovascular and mortality outcomes and association analyses with common clinical exposures. A standardized dataset was developed, with a federated analysis conducted using standardized statistical scripts. The summary-level outputs generated were then shared with the coordinating centers for aggregating and meta-analysis (Methods in the Data Supplement).

### Participant Characteristics

Detailed characteristics of participants by study are presented in Table 2. Prevalence of risk factors varied by study, with diabetes mellitus ranging from 4% to 76%; smoking from 8% to 79%. Mean total cholesterol by study ranged from 166.3 to 239.8 mg/dL, mean body mass index ranged from 24.8 to 30.2 kg/m^2^ and mean systolic blood pressure from 117 to 153 mm Hg. The proportion of participants with prior revascularization or MI was high in most studies reflecting the inclusion criteria for the consortium (Table 2).

Review of returned outputs from the federated analysis revealed good quality data with estimates falling within expected ranges for age, sex, and other variables, such as body mass index (Figure I in the Data Supplement).

### End Points

The primary end point preselected for the study was a composite of coronary death or MI (CHD death/MI). Mean follow-up was estimated in each study and ranged between 9 months and 15 years. In total, we estimated over 748 000 person-years of follow-up were available for the primary end point analysis.

Information was collected on 10 subsequent event end points in the initial feasibility analysis. Across all studies, the most frequently occurring event during prospective follow-up was the composite of all cardiovascular events (27%); followed by revascularization (21.8%); all-cause mortality (15%); coronary death or MI (14.2%); MI (10.7%); cardiovascular death (8.3%); coronary death (8%); heart failure (6.3%); all stroke (3.6%); and ischemic stroke (3.4%).

### Association Analyses

As a feasibility analysis, we examined associations between age, male sex, and smoking with the primary end point CHD death/MI as well as with the 9 other secondary end points, to investigate any differential associations across discrete subsequent events.

**Figure 3.**
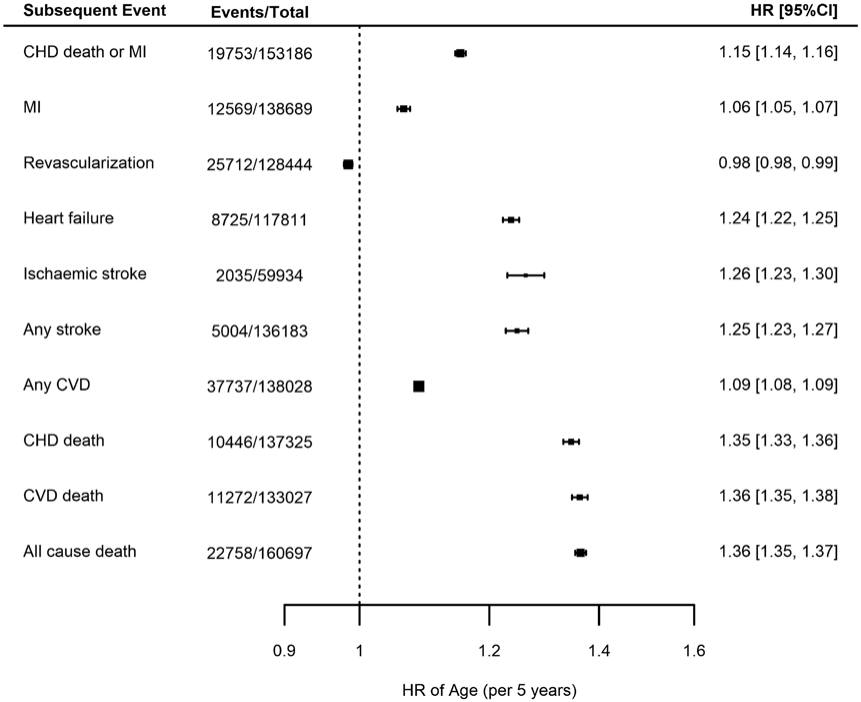
**Meta-analyses of the associations between age (per 5-year intervals) and different end points, adjusted for sex**. Estimates are presented as hazard ratios (HRs) with 95% CI. CHD indicates coronary heart disease; CVD, cardiovascular disease; and MI myocardial infarction.

In analyses unrestricted by race or type of CHD at baseline, but adjusted for sex, there was a strong association between each 5-year increment in age with subsequent risk of the primary end point of CHD death/MI (hazard ratio [HR] 1.15; 95% CI, 1.14–1.16). The largest observed HRs were for all-cause mortality (HR, 1.36; 95% CI, 1.35–1.37), cardiovascular death (HR, 1.36; 95% CI, 1.35–1.38), and heart failure (HR, 1.25; 95% CI, 1.24–1.27), while a smaller risk increase was observed for MI (HR, 1.06; 95% CI, 1.05–1.07). The risk of future revascularization, however, showed a modest inverse association with increasing age (HR, 0.98; 95% CI, 0.98–0.99; Figure 3).

**Figure 4.**
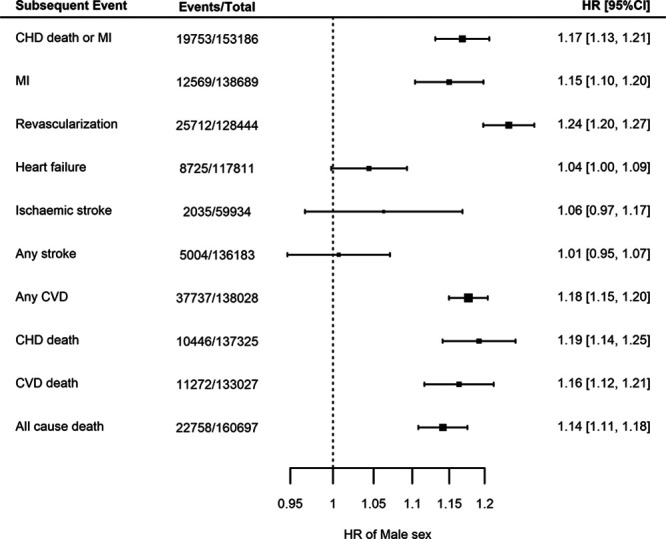
**Meta-analyses of the associations between male sex and different end points, adjusted for age.** Estimates are presented as hazard ratios (HRs) with 95% CI. CHD indicates coronary heart disease; CVD, cardiovascular disease; and MI, myocardial infarction.

Male sex was a risk factor for CHD death/MI (HR, 1.17; 95% CI, 1.13–1.21) and other coronary and mortality end points (Figure 4) after adjustment for age. In particular, the largest observed HR was for risk of revascularization, which was considerably higher in males (HR, 1.24; 95% CI, 1.20–1.27). In contrast, there was no strong evidence for an association between male sex and risk of stroke (ischemic or any stroke; Figure 4).

**Figure 5.**
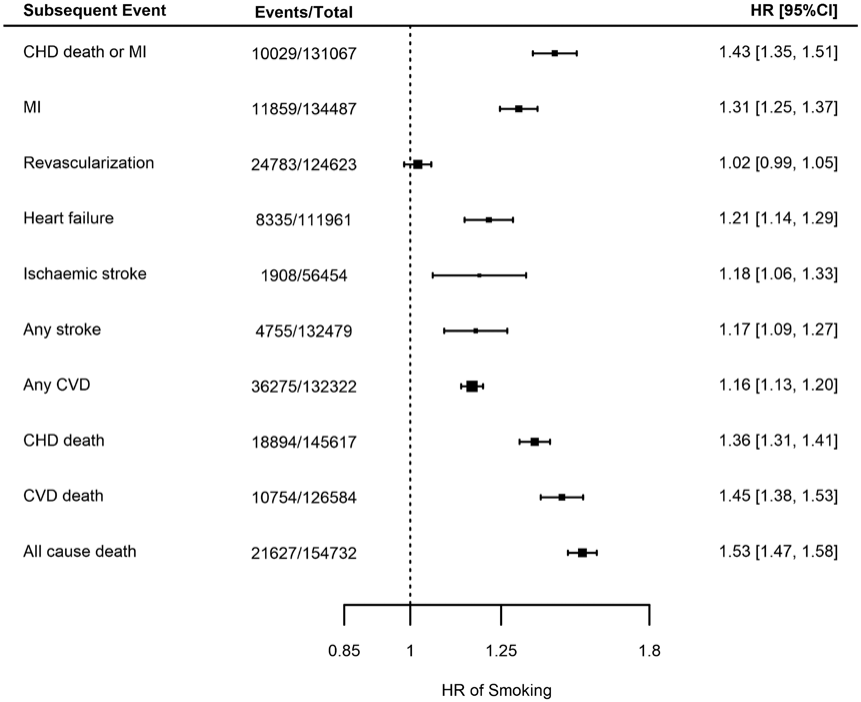
**Meta-analyses for the associations between smoking at coronary heart disease (CHD) indexing event compared to not smoking and for different end points, adjusted for age and sex.** Estimates are presented as hazard ratios (HRs) with 95% CI. CVD indicates cardiovascular disease; and MI, myocardial infarction.

Finally, in analyses adjusted for age and sex, current smoking (compared to prior or never smoking) at the time of enrollment showed a strong association with risk of future CHD death/MI (HR, 1.43; 95% CI, 1.35–1.51). Similarly, smoking was associated with an increased risk of all-cause mortality (HR, 1.53; 95% CI, 1.47–1.58) and an increased risk of all other end points, although there was no strong evidence for an association with incident revascularization (HR, 1.02; 95% CI, 0.99–1.05; Figure 5).

When stratified by type of CHD at enrollment, that is, among those presenting with an acute event, those with stable CAD without ever having had an MI and those with stable CAD and a prior MI, the findings were similar and directionally concordant to nonstratified analyses described above, for all end points (data not shown).

## Discussion

The GENIUS-CHD Consortium is a global collaborative effort engaging 57 studies, including almost 185 000 patients with established CHD, for whom genetic and prospective follow-up data are available. It brings together over 170 domain experts, including clinicians, data scientists, geneticists, and epidemiologists, all engaged in improving our understanding of the determinants of subsequent event risk in these patients. With an agreed governance structure and a proven federated analysis approach, we anticipate that this consortium will be a valuable long-term resource for genetic and nongenetic research in this field.

Genetic association studies for CHD disease progression, recurrence, and adverse events after a CHD event may have particular utility for identifying novel causal pathways and therapeutic targets that may be different than those for first events, a concept recently supported by research in other disease areas.^21^ However, information on the determinants of subsequent CHD event risk is scarce, in contrast to the extensive knowledge about risk factors for a first CHD event. This disparity is due, in part, to the relatively small sample sizes of individual studies in the secondary prevention setting. While larger registry and electronic health care records efforts will result in higher numbers, they typically suffer from the lack of necessary depth of phenotyping, accuracy, and availability of biospecimens to infer further biological insights.^22,23^ In contrast, large population studies with detailed phenotyping have relatively small numbers of mostly stable CHD patients, who have survived many years after their index event.^24,25^ By bringing together multiple investigator-led studies, the GENIUS-CHD consortium aims to address and overcome this major limitation to subsequent CHD risk research.

Importantly, the scale and depth of the GENIUS-CHD consortium offer greater scope to tackle key challenges within subsequent CHD risk-related research. First, CHD is a heterogeneous phenotype, consisting of stable, unstable, and pathologically distinct subtypes, which have often been combined for individual studies to satisfy the need for statistical power. With the sample size available in GENIUS-CHD, we anticipate being able to disaggregate CHD into more precise subphenotypes such as acute versus stable CHD at baseline, or those with versus without prior MI, which may help uncover relevant biological differences.^26^ Additional stratification on variables such as sex, time period of recruitment, duration of follow-up, country of study, LV function and treatment (such as statin, blood pressure lowering, and antiplatelet agent use) will also be possible, providing greater insights into the modifying influences of these variables on outcome.

A major strength of the consortium is the use of a federated analysis approach that permits individual level analysis without the need for sharing either samples or the individual datasets themselves, thereby overcoming major privacy and governance hurdles. The effort has been successful because (1) participation is entirely voluntary, with studies only participating in those analyses they feel are of value, or to which they have the capacity to contribute; (2) ownership of all data and samples remain with the principal investigator and are not shared nor stored centrally; and (3) there are open and transparent governance procedures. Our feasibility analysis has demonstrated that this federated approach works well and yields results that are consistent and suitable for high-quality meta-analysis.

Indeed, supported by this initial feasibility analysis, our findings demonstrate the validity of the data collected by confirming the anticipated associations of increasing age, male sex, and current smoking with higher risks of subsequent CHD death/MI during follow-up. Furthermore, by exploring multiple individual and composite end points, we can begin to unravel associations not discoverable in smaller studies. For example, we find that the risk of incident revascularization is lower with advancing age but higher for male sex and neutral for smoking. Plausible explanations may exist for each of these findings (eg, an association induced by clinical practice, with fewer older people being offered invasive treatments), but importantly they highlight the value of exploring multiple end points at an appropriate scale. This is especially relevant when exploring novel biomarkers or drug targets as these may, in turn, be used to inform clinical testing strategies and choice of end points to study in trials.

By virtue of the expertise it has assembled, the consortium is also well placed to address important methodological issues surrounding prognosis research in general. For example, selection bias is a key concern, whereby it is conceivable that those at highest risk may die early and not enter any of the member studies for evaluation (survival bias), or selection on an indexing event itself may distort patient characteristics and impact association findings (index event bias).^27^ In addition, treatment effects may alter the trajectory of disease by stabilizing or regressing plaque burden or altering baseline risk, such as with high-dose statin or PCSK9 inhibitor use.^17,28^ To address these and other issues, the consortium has established working groups of relevant national and international experts to explore the extent and impact of such biases/effects and if needed, to develop approaches to address these.^29^

There are inherent challenges to overcome when working with diverse multiple studies, including variations in definitions and processes for data collection and curation across different studies in different centers and different countries. The consortium members have attempted to standardize common data elements, for example, the measurement units for quantitative traits. Variability between studies will persist, but we anticipate that the overall size of the effort will help reduce the impact of such study level heterogeneity on any findings, which will also be explored through subgroup analyses where possible (eg, country, study size, and year of first recruitment). Analytical challenges will additionally include dealing with variability in length of follow-up across studies, handling multiple subsequent events along with competing risks, as well as confounding by treatment and selection biases as described above. The collective experience of the consortium members will be leveraged to address these as carefully as possible within each future analysis. Finally, factors influencing enrollment into genetic studies of CHD may limit the generalizability of findings. Men are over represented in participating CHD studies, partly reflecting sex-differential prevalence of disease but also underpinning a wider concern about underinvestigation of women, who may be inadvertently excluded given that entry criteria for most studies relies on documented presence of CHD. Similarly, many studies in the consortium have recruited mostly Europeans, limiting the opportunity to explore hypotheses in other ethnic groups. The steering committee is conscious of these imbalances and is actively seeking studies enriched for women and non-European participants to join the collaboration. In summary, the GENIUS-CHD consortium is a global collaboration among investigators who have recruited patients with CHD into multiple individual studies, seeking to gain a better understanding of subsequent CHD event risk and enhance secondary prevention. It seeks to be an open, collegiate, and transparent effort and we invite investigators with suitable studies to join and collectively enhance research efforts in this domain.

## Acknowledgments

The GENIUS-CHD collaborators would like to express their immense gratitude to all patients who participated in each of the individual studies as well as the many personnel who helped with recruitment, collection, curation, management, and processing of the samples and data.

## Sources of Funding

The funder(s) of the study had no role in study design, data collection, data analysis, data interpretation, or writing of the report. Within GENIUS-CHD, all participating investigators and sponsors who contributed data and analyses are acknowledged irrespective of academic or industry affiliations. Specific funding statements: Dr Patel is funded by a British Heart Foundation Intermediate Fellowship (FS/14/76/30933). This research was also supported by the National Institute for Health Research University College London Hospitals Biomedical Research Center; Dr Schmidt is funded by BHF grant PG/18/5033837; Dr Holmes works in a unit that receives funding from the UK Medical Research Council and is supported by a British Heart Foundation Intermediate Clinical Research Fellowship (FS/18/23/33512) and the National Institute for Health Research Oxford Biomedical Research Center; The AGNES study (Arrhythmia Genetics in The Netherlands) was supported by research grants from the Netherlands Heart Foundation (2001D019, 2003T302, 2007B202, and the PREDICT project [CVON 2012-10]), the Leducq Foundation (grant 05-CVD) and the Center for Translational Molecular Medicine (CTMM COHFAR).; The Cleveland Clinic Genebank Study was supported in part by NIH grants R0133169, R01ES021801, R01MD010358, and R01ES025786, R01HL103866, R01DK106000, R01HL126827, P20HL113452, P01HL098055, P01HL076491, and R01HL103931; The Clinical Cohorts in Coronary disease Collaboration (4C) study was supported in part by National Institute for Health Research (NIHR) and Barts Charity; The Corogene study was supported by grants from Aarno Koskelo Foundation, Helsinki University Central Hospital special government funds (EVO nos. TYH7215, TKK2012005, TYH2012209, and TYH2014312), and Finnish Foundation for Cardiovascular research; CABGenomics was supported by Stanton Shernan, C. David Collard, Amanda A. Fox/R01 HL 098601 National Heart, Lung, and Blood Institute (NHLBI); The Coronary Disease Cohort Study (CDCS) & Post Myocardial Infarction study (PMI) were funded by the Health Research Council and Heart Foundation of New Zealand; Dr Samman-Tahnan is supported by the National Institutes of Health/National Institutes of Aging grant AG051633; Dr Sandesara is supported by the Abraham J. & Phyllis Katz Foundation (Atlanta, GA); The Emory Cardiovascular Biobank is supported by National Institutes of Health (NIH) grants 5P01HL101398-02, 1P20HL113451-01, 1R56HL126558-01, 1RF1AG051633-01, R01 NS064162-01, R01 HL89650-01, HL095479-01, 1U10HL110302-01, 1DP3DK094346-01, and 2P01HL086773-06A1; The Estonian Biobank was funded by EU H2020 grant 692145, Estonian Research Council Grant IUT20-60, IUT24-6, PUT1660, PUT735, and European Union through the European Regional Development Fund Project No. 2014-2020.4.01.15-0012 GENTRANSMED, NIH-GIANT, ERA-CVD grant Detectin-HF and 2R01DK075787-06A1; FAST-MI (French Registry of Acute ST-Elevation or non–ST-elevation Myocardial Infarction) 2005 is a registry of the French Society of Cardiology, supported by unrestricted grants from Pfizer and Servier. Additional support was obtained from a research grant from the French Caisse Nationale d’Assurance Maladie; GENESIS-PRAXY is funded by the Canadian Institutes of Health Research and Heart and Stroke Foundations of Alberta, NWT & Nunavut, British Columbia and Yukon, Nova Scotia, Ontario, and Quebec (HSFC); The GENDEMIP study was supported by Project (MH, Czech Republic) No. 00023001 (ICEM, Prague); GoDARTS was funded by the Wellcome Trust (072960/Z/03/Z, 084726/Z/08/Z, 084727/Z/08/Z, 085475/Z/08/Z, and 085475/B/08/Z) and as part of the EU IMI-SUMMIT programme. Dr Palmer has received grant funding from the Wellcome Trust to develop the GoDARTS cohort; Dr Mordi is supported by an NHS Education of Scotland/Chief Scientist Office Postdoctoral Clinical Lectureship (PCL 17/07); the GENECOR study was supported in part by the Italian Ministry of Research’s Fund for Basic Research (FIRB 2005); GRACE UK was supported in part by an Educational Grant from Sanofi Aventis; Award from Chief Scientist Office, Scotland; INVEST-GENES was supported by the National Institute of Health Pharmacogenomics Research Network grant U01-GM074492, NIH R01 HL074730, University of Florida Opportunity Fund, BASF Pharma and Abbott Laboratories; IATVB was supported by Epidemiologia e Genetica della Morte Improvvisa in Sardegna; The KAROLA study has received financial support by the German Ministry of Education and Research (01GD9820/0 and 01ER0814), by the Willy-Robert-Pitzer Foundation, and by the Waldburg-Zeil Clinics Isny; The KRAKOW GENIUS Study was supported by a grant from the Polish Ministry of Science and Higher Education, no. NN402083939 and the National Science Centre, no. 2013/09/B/NZ5/00770; LIFE-Heart was funded by the Leipzig Research Center for Civilization Diseases (LIFE). LIFE is an organizational unit affiliated to the Medical Faculty of the University of Leipzig. LIFE is funded by means of the European Union, by the European Regional Development Fund (ERDF) and by funds of the Free State of Saxony within the framework of the excellence initiative; The LURIC study was supported by the 7th Framework Program (AtheroRemo, grant agreement number 201668 and RiskyCAD, grant agreement number 305739) of the European Union; Dr Smith was supported by grants from the European Research Council, Swedish Heart-Lung Foundation, the Swedish Research Council, the Crafoord Foundation, governmental funding of clinical research within the Swedish National Health Service, Skåne University Hospital in Lund, and the Scania county, a generous donation from the Knut and Alice Wallenberg foundation to the Wallenberg Center for Molecular Medicine at Lund University, and funding from the Swedish Research Council and Swedish Foundation for Strategic Research to the Lund University Diabetes Center; The NEAPOLIS CAMPANIA study was suppported by European Research Council Advanced Grant (CardioEpigen, no. 294609); Italian Ministry of Health (PE-2013-02356818); Italian Ministry of Education, University and Research (2015583WMX); The North East Poland Myocardial Infarction Study was supported by grant no. 402 529139 from the National Science Center (Poland); Dr Vilmundarson is supported by a graduate fellowship of the University of Ottawa Heart Institute; OHGS was funded in part by a Heart and Stroke Foundation grant; Dr Stott was supported in part by an investgator initiated grant from Bristol Myers Squibb; The PROSPER study was supported by an investigator initiated grant obtained from Bristol-Myers Squibb. Dr Jukema is an Established Clinical Investigator of the Netherlands Heart Foundation (grant 2001 D 032). Support for genotyping was provided by the seventh framework program of the European commission (grant 223004) and by the Netherlands Genomics Initiative (Netherlands Consortium for Healthy Aging grant 050-060-810).; The RISCA study was supported in part by FRSQ, HSFC, Merck Frost Canada, Pfizer Canada; The SHEEP study was supported by grants from the Swedish Council for Work Life and Social Research, and the Stockholm County Council; The TNT trial was sponsored by Pfizer who granted access to data, Genotyping of the samples was funded in part by grants from Genome Canada and Genome Quebec and the Canadian Institutes of Health Research (CIHR); Dr Arsenault holds a junior scholar award from the Fonds de recherche du Quebec-Sante (FRQS); Dr Cresci is supported, in part, by the National Institutes of Health (Cresci R01 NR013396). The TRIUMPH study was sponsored by the National Institutes of Health: Washington University School of Medicine SCCOR Grant P50 HL077113; The UCP studies were funded by the Netherlands Heart Foundation and the Dutch Top Institute Pharma Mondriaan Project; The Verona Heart Study was supported by the Cariverona Foundation; Veneto Region; Italian Ministry of Education, University, and Research (MIUR); LURM (Laboratorio Universitario di Ricerca Medica) Research Center, University of Verona; The Warsaw ACS Registry is supported by grant No. R13 0001 06 from The National Centre for Research and Development (NCBiR), Statutory Grant from Medical University of Warsaw; Dr Nelson is funded by the British Heart Foundation; Dr Samani is funded by the British Heart Foundation and is a NIHR Senior Investigator; Dr Hingorani is a NIHR Senior Investigator. Dr Asselbergs is supported by UCL Hospitals NIHR Biomedical Research Centre, EU/EFPIA Innovative Medicines Initiative 2 Joint Undertaking BigData@Heart grant no. 116074, the European Union’s Horizon 2020 research and innovation programme under the ERA-NET Co-fund action No. 01KL1802 (Druggable-MI-gene) jointly funded by the Dutch Heart Foundation and Netherlands Organization for Health Research and Development (ZonMw).

## Disclosures

Dr Patel has received speaker fees and honoraria from Amgen, Sanofi, and Bayer and research funding from Regeneron; Dr Holmes has collaborated with Boehringer Ingelheim in research, and in accordance with the policy of the The Clinical Trial Service Unit and Epidemiological Studies Unit (University of Oxford), did not accept any personal payment; Dr Akerblom has received institutional research grant and speakers fee from AstraZeneca, institutional research grant from Roche Diagnostics; Dr James has received grants from AstraZeneca, The Medicines Company, Swedish heart and lung foundation, Swedish research council, Janssen; personal fees from Bayer; Dr Hagstrom declares being an expert committee member, lecture fees, and institutional research grant from Sanofi, and Amgen; institutional research grants from AstraZeneca, and GlaxoSmithKline; expert committee member and lecture fees NovoNordisk and Behringer; Dr Held declares institutional research grant, advisory board member and speaker’s bureau from AstraZeneca; institutional research grants from Bristol-Myers Squibb Merck & Co, GlaxoSmithKline, Roche Diagnostics. Advisory board for Bayer and Boehringer Ingelheim; Dr Lindholm has received institutional research grants from AstraZeneca, and GlaxoSmithKline; Speaker fees from AstraZeneca, Speaker fees from AstraZeneca; Dr Siegbahn has received institutional research grants from AstraZeneca, Boehringer Ingelheim, Bristol-Myers Squibb/Pfizer, Roche Diagnostics, GlaxoSmithKline; Dr ten Berg reports receiving fees for board membership from AstraZeneca, consulting fees from AstraZeneca, Eli Lilly, and Merck, and lecture fees from Daiichi Sankyo and Eli Lilly, AstraZeneca, Sanofi and Accumetrics; Dr Wallentin reports institutional research grants, consultancy fees, lecture fees, and travel support from Bristol-Myers Squibb/Pfizer, AstraZeneca, GlaxoSmithKline, Boehringer Ingelheim; institutional research grants from Merck & Co, Roche Diagnostics; consultancy fees from Abbott; and holds a patent EP2047275B1 licensed to Roche Diagnostics, and a patent US8951742B2 licensed to Roche Diagnostics; Dr Asselbergs has received research funding from Regeneron, Pfizer, Sanofi.

## Supplementary Material
