# Supplementary material for: Subsequent Event Risk in Individuals With Established Coronary Heart Disease: Design and Rationale of the GENIUS-CHD Consortium
Source: Circ Genom Precis Med. 2019 Apr 16;12(4):e002470. doi: 10.1161/CIRCGEN.119.002470 (PMC6629546; doi:10.1161/CIRCGEN.119.002470)
Supplement: Supplementary file 1 [file hcg-12-e002470-s001.pdf]

SUPPLEMENTAL MATERIAL

1 Supplemental Methods

The GENIUS-CHD consortium builds on previous successful collaborations between individual investigator-led studies.[1-3] We identified additional studies meeting eligibility criteria (listed below) through a combination of literature, article bibliography and conference abstract searches as well as established investigator networks. Principal investigators (PIs) were invited to participate in the GENIUS-CHD consortium with membership remaining open to any study meeting eligibility criteria listed in **Figure 1** (in the main manuscript).

Terminology

In this article and for future consortium analyses, we use the term "subsequent" to refer to all coronary and cardiovascular diagnoses, procedures or events including death that occur after enrolment into a study, which may be at the time of an index CHD event (for ACS studies) or later.

Eligibility

Studies were eligible to join the GENIUS-CHD consortium if they:

1. Included individuals with established CHD. CHD was defined as the presence of or history of acute coronary syndrome at baseline, or of coronary artery disease as evidenced by any revascularization procedure (percutaneous coronary intervention or bypass surgery) or demonstrable plaque in any epicardial vessel on direct coronary imaging. One study also included physician confirmed stable angina but only as part of a wider definition of established CHD.[4]
2. Acquired prospective follow-up of participants with ascertainment of one or more subsequent cardiovascular disease events as well as all-cause mortality. No minimum time frame or person-years of follow up was required.

3. Stored blood samples for these individuals, which are viable and suitable for DNA and/or biomarker analysis or have previously collected such data prior to sample depletion.

Participating studies

We list studies participating in the GENIUS-CHD consortium at the time in **Table 1** of the main manuscript. Please refer to www.genius-chd.org for an updated list.

Following agreement to participate, each investigator submitted brief details about their study including study design, inclusion criteria and number of participants, available clinical and research phenotypes, sample availability and endpoints, using an online questionnaire with the results collated by the central analysis sites at University College London (UCL), UK and University Medical Centre (UMC) Utrecht, Netherlands. Full details describing the majority of studies have been published individually, and PubMed IDs are listed in **Table 1** of the main manuscript.

Participating studies received local ethical committee approval, and all included patients who provided informed consent at the time of enrolment. The central analysis sites also received favourable ethical reviews for collating and analysing summary level data from these individual studies.

Consortium management

The consortium represents a voluntary collaboration between multiple studies and management is based on a set of principles outlined in a memorandum of understanding (available at www.genius-chd.org).

A scientific steering committee, comprising of at least one representative from each study, represents the consortium, and is the decision-making group for projects and consortium activity. A smaller

committee oversees operational and management aspects for the group. Finally, smaller project-specific working groups (PSWG) initiate, lead and run individual approved projects reporting to the main steering committee. The overall structure of the consortium is presented in **Figure 1** in the main manuscript. More detail on the specific activities and membership of these subgroups is available at www.genius-chd.org.

Approach to Analysis

The consortium operates using a federated analysis approach whereby individual level data are analysed locally at each site using centrally developed and standardized statistical scripts, distributed to each group along with relevant instructions. Thereafter, analysis teams from individual studies share summary level outputs with the central analysis sites for meta-analyses.

For each study, a core standard dataset is first developed. Preparatory work conducted by the UCL/UMC Utrecht teams has ensured that standardized units and definitions are used for the variables of interest. Additionally, detailed and adaptable statistical scripts are available based on the open source statistical software package R,[5] allowing additional projects to be initiated in a timely fashion and by a standardized and easily implementable workflow.

Planned projects

Projects to be prioritized by the consortium include core internal proposals, developed by the steering committee, as well as external proposals, either from consortium members seeking validation and replication of their own study findings, or from external investigators who wish to run a *de novo* project within the consortium. All new projects are subject to approval by the scientific steering committee

based on feasibility, interest and scientific value. Individual studies are entitled to opt in or out of the proposed analyses at the discretion of their respective principal investigators.

Instructions and relevant information for submission of new proposals are on the website to guide investigators wishing to submit new proposals. Leaders of approved projects are asked to nominate a PSWG and to report on progress at regular intervals to the steering committee.

Future proposals will seek to perform *de novo* genotyping and run biomarker assays on stored samples. In such cases and wherever possible, core or central laboratories will be identified within each country/continent to facilitate this. Sharing of individual level data or biological materials outside of the host institution, should it be required, will be subject to relevant and strict ethical and review board approvals from each site. A major strength of the federated analysis approach is that it avoids the need for sharing individual level data, and only summary level estimates are shared, even though analyses have been performed using individual level data in a standardized way ensuring high quality and comparable results. This design also circumvents the need to transfer samples to a central processing site, avoiding ethical concerns associated with sending samples out of the country of origin.

Feasibility analysis

To help establish the consortium and demonstrate feasibility of this approach we developed an initial analysis plan, dataset of summary estimates and statistical script to collect descriptive data and examine the association of age, sex and smoking at baseline on the time to a primary outcome of subsequent CHD death or MI. Secondary endpoints included time to event: MI, revascularization, heart failure, stroke, ischemic stroke, CHD death, cardiovascular death, all cause death and a cardiovascular composite comprised of MI, revascularization, stroke or CV death. All time-to-event analyses were based

on a Cox proportional hazards model, with the proportional hazard assumption tested using a Chi-square “goodness of fit” statistic.[6]

Study-specific results were collected and central processing was performed jointly by the UMC Utrecht and UCL teams. Point estimates (ORs or HRs) and their corresponding standard errors were meta-analysed using the fixed effect inverse variance weighted method; random effects estimates are included in supplementary tables 5-7. Study specific proportional hazard tests were combined using Fisher’s method for p-value meta-analysis.[7] All analyses at the coordinating centres were conducted using R statistical software (v 3.4.1).[5]

Brief cohort descriptions

1) 4C - Clinical Cohorts in Coronary Disease Collaboration

4C was a cohort study of 1,493 patients with coronary artery disease that ran between 2009 and 2014. The patients underwent deep clinical, metabolomic and genetic phenotyping for identification of novel prognostic markers. The median follow-up was 2.43 years. All study participants gave written informed consent.

2) AGNES - Arrhythmia Genetics in the Netherlands

AGNES consists of patients presenting with a first acute ST-segment elevation MI that survived to hospital admission. Patients were recruited at seven heart centers in the Netherlands between 2001 and 2011. Individuals with an actual non-ST-segment elevation MI, prior MI, congenital heart defects, known structural heart disease, severe co-morbidity, electrolyte disturbances, trauma at presentation, recent surgery, previous coronary artery bypass graft or use of class I and III antiarrhythmic drugs. AGNES patients were of self-declared Dutch European descent. The Institutional Review Board of the Academic Medical Centre (University of Amsterdam) and other participating centres approved the AGNES study. All participants gave written informed consent [8].

3) ANGES - Angiography and Genes Study

The ANGES study population consists of 1,000 Finnish individuals who underwent coronary angiography at Tampere University Hospital to detect coronary artery disease between September 2002 and July 2005. Data were collected on age, sex, body mass index, alcohol consumption, smoking, medication as well as traditional risk factors of atherosclerosis. A clinical diagnosis of myocardial infarction was based on symptoms, electrocardiographic findings and biochemical marker tests measuring troponin I and creatine kinase. Previous cardiovascular diseases, therapeutic procedures and data on coronary artery disease and myocardial infarction were retrieved from patient records at Tampere University Hospital. Follow-up data were derived from the national health care registers maintained by the National Institute for Welfare and Health. The local ethical committee has approved the study and a written informed consent was obtained from all participants [9].

4) ATVB - Italian Atherosclerosis, Thrombosis and Vascular Biology Group

The ATVB study contributed 1,210 coronary heart disease cases to GENIUS analyses who were enrolled between January 1998 and January 2001. Cases were hospitalized for a first myocardial infarction before the age of 45 years and also underwent coronary arteriography at the time of hospitalization.
Study participants were given a standardized questionnaire including information on cardiovascular risk factors, medical diagnosis, lifestyle, and medication with collected data including age, sex, and traditional risk factors such as a family history of ischemic heart disease, smoking, high serum cholesterol levels, diabetes, hypertension, and cocaine use. Alcohol intake and the levels and pattern of physical exercise were also recorded. All of the study participants agreed to give blood samples for DNA analysis and cholesterol measurements. The Institutional Review Boards of the participating hospitals approved the study, and the cases and controls gave their written informed consent [10].

5) CABGenomics - Coronary Artery Bypass Genomics

The CABG Genomics Research Study is a prospective, ongoing two institution collaborative study conducted by investigators at the Department of Anesthesiology at Brigham and Women's Hospital, Boston, MA and at the Division of Cardiovascular Anesthesiology, Texas Heart Institute, St. Luke's Episcopal Hospital, Houston, TX. To date enrolled >3000 subjects undergoing primary coronary artery bypass graft (CABG) surgery for the primary purpose of identifying important genetic predictors of adverse postoperative outcomes. The study has active Institutional Review Board approval and patients signed written informed consents [11].

6) CardioLines

CardioLines is a study aiming to investigate the potential factors relating to success or failure of diagnosis and treatment amongst cardiovascular patients.

7) CDCS - Coronary Disease Cohort Study

The Coronary Disease Cohort Study (CDCS) is a cohort study of 2,140 patients with unstable angina or myocardial infarction admitted to Christchurch or Auckland Hospitals (New Zealand) from 2002-2009. Inclusion criteria were ischaemic discomfort plus one or more ECG change (ST-segment depression or elevation >=0.5mm, T-wave inversion of >=3mm in >=3 leads or left bundle branch block), elevated levels of cardiac markers, a history or coronary disease, age >=65 years and a history or diabetes or vascular disease. Patients were excluded if they had a severe comorbidity that reduced their life expectancy to <3 years. Anthropometric and clinical measures, including echocardiography and a large number of neurohormonal markers, were documented at baseline once patients were stable (approximately 1 month after hospital admission). Clinical outcomes were recorded by direct follow-up and from the New Zealand Health Information Service for a minimum of 3 years after admission (median 5 years, maximum 9.5 years). The study was approved by the New Zealand Multi-region Ethics Committee (CTY/02/02/018) and all patients provided written informed consent. The study was registered on the Australian New Zealand Clinical Trials Registry (ACTRN12605000431628).

8) COGEN - The Copenhagen Cardiovascular Genetic study

COGEN is a biobank of around 80,000 individuals recruited from six cardiology departments across Copenhagen, Denmark between 2010 and 2017.

9) COROGENE

COROGENE was a cohort of consecutive Finnish patients undergoing coronary angiogram between June 2006 and March, 2008 (n=5330), collected in the Helsinki University Central Hospital. The controls for COROGENE cases were selected from the greater Helsinki region participating for FINRISK 1997, 2002, and 2007 cohorts. All participants provided written informed consent [12].

10) CTMM - Circulating Cells

The Center for Translational Molecular Medicine (CTMM) – Circulating Cells is a prospective cohort study conducted in four Dutch medical centers. Between March 2009 and September 2011, the study enrolled 730 patients with stable or unstable angina pectoris undergoing coronary angiography. Details

on the study design have been described elsewhere [13]. Median follow-up was 300 days. Cardiovascular events during follow-up were verified by a clinical event committee. The study was approved by the ethical committees of the participating hospitals and was conducted in accordance with the Declaration of Helsinki.

11) CURE

CURE was an international, multicentre, randomized, parallel group trial of the combination of clopidogrel plus aspirin vs placebo plus aspirin in patients with acute coronary syndrome (unstable angina and non-Q wave myocardial infarction). The study involved 12,562 patients from 508 centres across 28 countries [14, 15].

12) EGCUT - Estonian Biobank

The Estonian Biobank is a population-based biobank of the Estonian Genome Center at the Institute of Genomics, University of Tartu (www.biobank.ee; EGCUT). The entire project is conducted according to the Estonian Gene Research Act and all of the participants have signed the broad informed consent. The cohort size is up to 52,000 individuals from 18 years of age and up, closely reflecting the age, sex and geographical distribution of the Estonian population. All of the subjects were recruited randomly by general practitioners and physicians between 2002 and 2011. A Computer Assisted Personal interview was filled within 1-2 hours at a doctor's office, including personal, genealogical, educational, occupational history and lifestyle data. Anthropometric measurements, blood pressure and resting heart rate were measured and venous blood taken during the visit. Medical history and current health status is recorded according to ICD-10 codes and the data are continuously updated through periodical linking to national electronic databases and registries.

Prevalent and incident CHD cases (n=2700) for this case-cohort study were obtained via linkage to the Estonian Health Insurance Fund (EHIF) database and the Estonian Causes of Death Registry. The mean follow-up time for this sample set is 6.6 years (SD 3.0 years) and median follow-up time 6.7 years.

13) EMORY - Emory Cardiovascular Biobank

The Emory Cardiovascular Biobank is a prospective cohort of 5,876 patients undergoing elective or emergent heart catheterization for suspected or confirmed CAD at 3 Emory healthcare sites in Atlanta, GA. Subjects with congenital heart disease and heart transplantation cancer were excluded. The study was approved by the Institutional Review Board at Emory University, Atlanta, GA, USA. All subjects provided written informed consent at the time of enrolment.

14) ERICO - Estratégia de Registro de Insuficiencia Coronariana

ERICO was a community hospital registry of 839 (mean age = 63 years-old; 60% men) patients with a medical diagnosis of the acute coronary syndrome. They were referred to primary care setting or private physician to evaluate the impact of the "real world" concern to secondary cardiovascular prevention. The enrolment period was from 2009 to 2014. The follow-up used the cold and hot pursuit of events. The contact was by phone periodically according to the study design [16].

15) FASTMI2005 – The French Registry of Acute ST-Elevation MI

FAST-MI (French Registry of Acute ST-Elevation or non- ST-elevation Myocardial Infarction) 2005 (NCT00673036) was a nationwide French registry that consecutively included patients with STEMI or NSTEMI admitted to cardiac intensive care units (ICU) within 48 hours of symptom onset. The primary objective was to evaluate the characteristics, management, and outcomes of AMI patients, as seen in routine clinical practice, on a country-wide scale. Of all centres managing AMI patients in France , including university teaching hospitals, general and regional hospitals, and private clinics receiving AMI emergencies, 60% (223 centres, 3059 patients during one month period and 3670 patients overall) participated in the study between October and December 2005. AMI was defined by increased levels of cardiac biomarkers together with either compatible symptoms or ECG changes. Exclusion criteria were (1) refusal to participate; (2) iatrogenic MIs, defined as occurring within 48 hours of any therapeutic procedure and (3) AMI diagnosis invalidated in favour of another diagnosis. Follow-up information was collected through contacts with the patients' physicians, the patients or their family, and registry offices of their places of birth. Events were adjudicated by a scientific committee whose members were unaware of patients' genotypes. The study was conducted in accordance with the

guidelines on good clinical practice and French law. A written consent was obtained from all participants. Additional written consent was obtained for those participating in the DNA bank. The study protocol was reviewed by the relevant Committee for the Protection of Human Subjects (CPP). Data file collection and storage were approved by the Commission Nationale Informatique et Liberté [17].

16) FINCAVAS - Finnish Cardiovascular Study

The Finnish Cardiovascular Study (FINCAVAS) includes 4,567 patients who underwent exercise stress tests at Tampere University Hospital. Study participants were followed up for major cardiovascular events, coronary procedures and cause of death with follow-up data gathered at 2, 5 and 10 years post-recruitment [18].

17) FRISCII - Fast Revascularisation during Instability in Coronary artery disease

The FRISC II study was a prospective randomised multicentre study of 3489 patients with non-ST-elevation acute coronary syndrome performed in 58 Scandinavian hospitals. The trial compared long-term (3 months) treatment with low molecular mass heparin (dalteparin = FRagmin) versus placebo and, in a factorial design, an early invasive versus a non-invasive treatment strategy. Outcome events were cardiovascular and total death, myocardial infarction, stroke and major bleeding. Complete follow-up with event adjudication was performed during the initial 2 years. Long-term follow-up by registries is available until 15 years. DNA for genetic analyses is available from 3606 patients.

18) GENDEMIP - GENetic Determination of Myocardial Infarction in Prague

Starting in 2004, data from men and women younger than 55 and 65 years, respectively, with acute coronary syndrome were collected from Coronary Care Unit at Clinic of Cardiology at Institute for Clinical and Experimental Medicine, Prague, Czech Republic. Information recorded included history of acute coronary syndrome, clinical course and procedures done at CCU. In addition, information regarding traditional cardiovascular risk factors including repeated measurements of nonfasting/fasting plasma lipids, glycemia and hsC-reactive protein during stay in hospital was obtained. In this population, samples for genetic analyses were collected. Patients were followed up in one year period on out-

patient visit or on mailing basis. Since 2016 age limitation was lifted and the data were more focused on clinical parameters, including quality of life and heart insufficiency questionnaires. Laboratory and genetic methods were moderately expanded. The methodology is the same. The number of included patients is recently approximately 1,600 men and 650 women. Questionnaire and list of laboratory measurements are available.

19) GENEBANK - Cleveland Clinic Genebank Study

The Cleveland Clinic GeneBank study is a single site sample repository generated from consecutive patients undergoing elective diagnostic coronary angiography or elective cardiac computed tomographic angiography with extensive clinical and laboratory characterization and longitudinal observation. Subject recruitment occurred between 2001 and 2007. Ethnicity was self-reported and information regarding demographics, medical history, and medication use was obtained by patient interviews and confirmed by chart reviews. All clinical outcome data were verified by source documentation. CAD was defined as adjudicated diagnoses of stable or unstable angina, myocardial infarction (MI) (adjudicated definition based on defined electrocardiographic changes or elevated cardiac enzymes), angiographic evidence of ≥ 50% stenosis of one or more major epicardial vessel, and/or a history of known CAD (documented MI, CAD, or history of revascularization). Prospective cardiovascular risk was assessed by the incidence of major adverse cardiac events (MACE) during three years of follow-up from the time of enrollment, which included nonfatal MI, nonfatal stroke, and all-cause mortality. Nonfatal events were defined as MI or stroke in patients who survived at least 48 hours following the onset of symptoms. Adjudicated outcomes ascertained over the ensuing 3 years for all subjects following enrollment were confirmed using source documentation. The GeneBank Study has been used previously for discovery and replication of novel genes and risk factors for atherosclerotic disease (refs given above). The present analysis included ~3703 Caucasian subjects for whom imputed genotype and CAD-related outcome data were available, depending on the phenotype in question.

20) GENESIS-PRAXY

GENESIS-PRAXY was a prospective, multicenter study of 1,210 patients aged 18-55 years and admitted to hospital with ACS. At baseline, questionnaires were administered, and anthropometric and biological measurements were performed. The patients were observed for at least 1 year, with additional questionnaires being administered at 1, 6, and 12 months post-discharge. A review of medical records were performed both at baseline and during follow-up [19].

21) GENOCOR – Genetic Mapping for Assessment of Cardiovascular Risk

The Italian GENOCOR (Genetic Mapping for Assessment of Cardiovascular Risk) study, included 1000 patients who were discharged between 1999 and 2007 with a diagnosis of ischaemic heart disease with patients given the option of voluntary genetic screening. Patients were eligible for inclusion in GENIUS analyses if they had all genotyping data and completed follow-up. The final cohort comprised 498 patients: 315 with MI and 183 with angina. The study was approved by the local ethics committee and written informed consent was obtained in all patients [20].

22) GEVAMI - The GEnetic causes to Ventricular Arrhythmia in patients during first ST-elevation Myocardial Infraction

The GEVAMI study is an ongoing prospective Danish case-control study among patients with first ST-elevation myocardial infarction (STEMI) between the ages of 18 and 80 years.(PMID:25559012) Cases are patients who experienced onset of ventricular fibrillation (VF) within the first 12 hours of symptoms of STEMI before guided catheter insertion for primary percutaneous coronary intervention (PPCI), and controls did not have VF during this time period or during PPCI. Baseline demographics and previous medical history are collected by research coordinators utilizing pre-designed questionnaires and whole blood is collected for genetic analysis. Follow-up on the patients is done by the Danish registries.

23/24) GoDARTS incident/prevalent

The Genetics of Diabetes Audit and Research in Tayside Scotland (GoDARTS) cohort profile has been described previously [21]. At the point of recruitment, all participants in GoDARTS provided, by invitation, informed consent for their data to be used for research purposes and explicit consent for use in collaboration with industry. The study complies with the Declaration of Helsinki.

25/26) GRACE_Belgium & GRACE_UK - Global Registry of Acute Coronary Events

The GRACE Genetics Study consists of the 3473 patients from whom DNA was collected by the eight participating centres in three countries (UK, Belgium, and Poland) between May 2001 and June 2007. Collected blood samples were transferred to the core laboratory (The Queens Medical Research Institute, Edinburgh, UK), where DNA was extracted and stored at minus 80C.

Patients were eligible if they were admitted to participating hospitals with a clinical diagnosis of ACS and at least one of the following features: electrocardiographic changes consistent with ACS, increases in serum biochemical markers of cardiac necrosis, documented prior MI; congestive heart failure believed to be due to ischaemia or resuscitated sudden death; history of/or new positive stress test or angina, with or without imaging; prior or new cardiac catheterization documenting artery disease, percutaneous coronary intervention (PCI), or coronary artery bypass graft (CABG)], or both.

To ensure enrolment of an unselected ACS population, sites recruited the first 10–20 consecutive eligible patients each month. Regular audits were performed at all participating hospitals to confirm conformance to eligibility criteria and correct interpretation of data in the case report form. Trained study coordinators collected data using standardized case report forms.

Demographic characteristics, medical history, presenting symptoms, duration of pre-hospital delay, biochemical and electrocardiographic findings, treatment practices, and outcomes were collected. All cases were assigned to one of the following categories using predefined criteria: (i) STEMI (including left bundle-branch block), (ii) non-STEMI, (iii) UA, and (iv) other cardiac or (v) non-cardiac diagnoses [22, 23].

27) IDEAL - Incremental Decrease in End Points Through Aggressive lipid Lowering

The IDEAL RCT was a prospective, randomized, open-label, blinded end-point evaluation, multicentre study of the occurrence of cardiovascular

events in patients treated with atorvastatin 80 mg/d or usual-dose simvastatin 20 mg/d to lower LDL-cholesterol as much as possible. Men or women up to 80 years of age either hospitalized with a definite acute myocardial infarction or with a history of definite myocardial infarction, eligible for statin therapy according to recent national guidelines [24].

28) INTERMOUNTAIN - Intermountain Heart Collaborative Study

The Intermountain Heart Collaborative Study is a registry encompassing a prospectively enrolled cohort of cardiac catheterization patients who electively, urgently, or emergently underwent coronary angiography for suspected coronary heart disease at the Intermountain Medical Center, LDS Hospital, and Cottonwood Hospital in Salt Lake City, Utah. The registry was established in 1994 and patient enrollment is ongoing.[25, 26] All cohort members were treated per clinical guidelines for coronary artery disease and myocardial infarction depending on the findings of angiography and other diagnostic testing, with treatments including revascularization (i.e., percutaneous coronary intervention or coronary bypass surgery) with medical therapy, or medical therapy alone. Cohort follow-up used passive surveillance of electronic health records from Intermountain Healthcare's network of 22 hospitals and >180 clinics in Utah and southeast Idaho, a geography in which Intermountain serves the healthcare needs of approximately two thirds of the population. Mortality was determined from those records, Utah death certificates, and the Social Security death master file.

29) INVEST - INternational VErapamil SR Trandolapril Study Genetic Substudy

INVEST (INternational VErapamil SR Trandolapril STudy) was an international, multicenter, randomized controlled clinical trial of 22,576 hypertensive individuals with documented coronary artery disease of ≥50 years old [27]. Patients were randomized to either a calcium channel blocker (Verapamil SR)-based treatment strategy (CCB strategy) or a β-blocker (atenolol)-based treatment strategy (β-blocker strategy), with HCTZ and trandolapril available as add-on agents in both strategies. The genetic substudy of INVEST (INVEST-GENES) includes DNA samples from 5,979 INVEST participants from the United States, including Puerto Rico. All study protocols were approved by local or central institutional review boards and all participants provided separate, voluntary, written informed consent for participation in INVEST and INVEST-GENES.

30) JUMC - Krakow-GENIUS-CHD

Between 2010-2015 in Jagiellonian University Medical College (Krakow, Poland), patients were enrolled into one of the two following studies: VISION (*Vascular Events in Noncardiac Surgery Patients Cohort Evaluation study*; 900 patients from Polish cohort undergoing vascular surgery) or LTIMI (*Leukotrienes*

and Thromboxane In Myocardial Infarction; 261 MI patients). Protocols for recruitment have been described elsewhere [28, 29]. From these 2 cohorts, patients with documented coronary artery disease (both acute coronary syndromes and stable coronary artery disease) were eligible to participate in the GENIUS project (n=747). DNA samples for genetic analyses were available for 704 subjects (94%). Cardiovascular mortality was defined as any death related to cardiovascular reasons obtained in the follow-up, including: sudden cardiac death, fatal myocardial infarction, death due to congestive heart failure, or death immediately after intervention to treat CAD, as well as fatal stroke. The median follow-up of study participants was 360 days with 0.3% of the sample lost to follow-up. All patients signed written informed consent to participate according to the local ethics committee and with accordance with the Helsinki Declaration.

31) KAROLA

The KAROLA study is a prospective cohort study comprising subjects that were recruited in 1999 and 2000 in two rehabilitation clinics in middle and southern Germany (Klinik am Südpark, Bad Nauheim; Schwabenland-Klinik, Isny-Neutrauchburg). All patients aged 30–70, who were undergoing inpatient cardiovascular rehabilitation in one of these clinics because of the recent occurrence of an acute cardiovascular event or procedure (acute coronary syndrome, acute myocardial infarction, coronary artery revascularisation) within the past 3 months before admission, were eligible for the study. Overall, the study included 1206 patients at baseline. The study protocol was approved by the ethics committees of the Universities of Ulm and Heidelberg, and by the ethics boards of the chambers of physicians of the federal states of Hessen and Baden-Wuerttemberg. Written informed consent was obtained from all participants before enrolment in the study.

Health related and sociodemographic data were collected from patients with self-administered standardised questionnaires at baseline. Additional information on medical findings and secondary diagnoses was obtained from hospital medical records. During follow-up, primary care physicians of patients were contacted to obtain medical information and incidence of non-fatal cardiovascular events. For patients deceased during follow-up, death certificates were retrieved from local health authorities and the main cause of death was coded according to the current International Classification of Diseases (ICD).

32) LIFE-HEART - Leipzig Heart Study

The LIFE-Heart study is an observational study of patients recruited at the Leipzig Heart Center, Germany. Patients with suspected coronary artery disease (CAD), stable CAD or myocardial infarction were recruited. Patients received a comprehensive assessment of vessel status and cardiologic function including coronary angiography, carotid ultrasound, ankle-brachial index, echo-cardiography and electrocardiography. Details of the study can be found in Beutner et al [30]. Genotyping information including quality control can be found in Pott et al [31].

The study was approved by the Ethics Committee of the Faculty of Medicine of Leipzig University, Germany (Reg. No 276-2005) and is registered at ClinicalTrials.gov (NCT00497887). Written informed consent was obtained from all participants included in the study.

33) LURIC - The LUdwigshafen RIsk and Cardiovascular Health Study

The Ludwigshafen Risk and Cardiovascular Health (LURIC) study is a monocentric hospital based prospective study including 3316 individuals referred for coronary angiography recruited in the Ludwigshafen Cardiac Center, southwestern Germany from 1997 – 2000 [32]. Clinical indications for angiography were chest pain or a positive non-invasive stress test suggestive of myocardial ischemia. To limit clinical heterogeneity, individuals suffering from acute illnesses other than acute coronary syndrome, chronic non-cardiac diseases and a history of malignancy within the five past years were excluded. All participants were completed a detailed questionnaire which gathered information on medical history, clinical, and lifestyle factors. Fasting blood samples were obtained by venipuncture in the early morning and stored for later analyses. Information on vital status during follow-up was obtained from local registries. Death certificates, medical records of local hospitals, and autopsy data were reviewed independently by two experienced clinicians who were blinded to patient characteristics and who classified the causes of death. Coronary heart disease (CHD) at baseline was defined as the presence of a visible luminal narrowing (>50% stenosis) in at least one of 15 coronary segments according to the classification of the American Heart Association. These patients were included in the current analysis. Samples were genotyped on an Affymetrix 6.0 array. Study protocols were approved by the ethics committee of the "Landesärztekammer Rheinland-Pfalz" and the study was conducted in accordance with the "Declaration of Helsinki”. Informed written consent was obtained from all participants.

34) MDCS - Malmo Diet and Cancer Study

Malmö Diet and Cancer is a prospective, community-based cohort study including 30,447 middle-aged participants from southern Sweden that underwent a baseline examination in 1991-1996. Participants filled out a questionnaire, underwent anthropometric measurements, and donated peripheral venous blood samples. Prevalent and incident diagnoses of myocardial infarction were identified by record linkage to national registers using personal identification numbers, The Swedish Hospital Discharge Register (HDR) and the Swedish Cause of Death Register (CDR) as described previously [33]. Subjects undergoing percutaneous coronary intervention (PCI) or coronary artery bypass graft (CABG) surgery for coronary heart disease were also identified from the HDR. In total, 2505 subjects were diagnosed with myocardial infarction during follow-up and were included in the current study. A total of 2041 individuals without infarction but undergoing PCI or CABG for coronary disease were also included. Information on death during follow-up was obtained from the CDR and cardiovascular mortality was defined as death from any code in the category of the International Classification of Disease 10th edition. All participants are residents of European descent. The local ethics committee approved of the study and written informed consent was obtained from all participants [33].

35) NE_POLAND - North East Poland Myocardial Infarction Study

The North East Poland Myocardial Infarction Study included 652 patients with ST-elevation myocardial infarction (STEMI) who survived the first 48 hours after hospital admission. They were all hospitalized in the years 2001-2005 and were subjected to log-term follow-up (mean 7.2 ± 2.8 years). Six hundred and three patients with completed follow-up and successful genotyping were included in the final analysis. All participants are residents of European descent. The local ethics committees have approved the protocol of the study. A written informed consent was obtained from all participants [34].

36) NEAPOLIS – NEAPOLIS CAMPANIA ITALIA

Neapolis is a cohort study of 1394 caucasian patients schedule to undergo elective DES implantation at the Clinica Mediterranea (Naples, Italy) between January 2008 and January 2010. One year follow up

was available for 75% of the patients. Exclusion criteria were either non-ST-elevation or ST-elevation myocardial infarction; cardiogenic shock; allergy/intolerance to aspirin and/or clopidogrel; serious bleeding or bleeding diathesis; platelet count ≤75.000/mm3; planned or undelayable noncardiac surgery; prior percutaneous coronary intervention (PCI) or coronary artery bypass graft; severe liver disease (e.g., cirrhosis or portal hypertension); and life expectancy <1 year due to other medical conditions. All enrolled patients gave informed consent prior to the index procedure. Diabetes mellitus (DM) was diagnosed according to current guidelines. Chronic kidney disease (CKD) was defined as an estimated glomerular filtration rate (eGFR) <60ml/min/1.73m2. This study was approved by the local ethic committee [35].

37) OHGS longitudinal - Ottawa Heart Genomics Study longitudinal

A total of 956 individuals were recruited to The Ottawa Heart Genomics Study (OHGS) longitudinal from the John and Jennifer Ruddy Canadian Cardiovascular Genetics Centre at The University of Ottawa Heart Institute, Ottawa, Canada, between 2010 and 2013, when recruitment ended. Recruited individuals were later sent a survey to follow-up on risk factors and behaviours related to CAD and hypertension. Participants with both survey and GWAS data available for the Lp(a) locus were included in the analysis. Institutional Research Ethics Board (REB) approval was obtained for this study. A total of 755 individuals participated in the survey and of those there were 522 with GWAS data available. Information related to cause of death of the patients during follow-up were obtained, with prior patient approval, from physician(s) at The University of Ottawa Heart Institute and/or any other institution were the patient would have sought medical attention for their cardiac condition. Median follow-up was 1.76 years with 21% of those recruited not participating in the follow-up survey. Cardiovascular mortality was defined as death related to CAD, death due to valve failure and death due to stroke.

38) PERGENE - Perindopril Genetic Association Study

The PERGENE study is a substudy of the EUROPA-trial [36] that will investigate genetic determinants of the treatment effect of ACE-inhibition in all subject. The EUROPA trial was a randomized, double-blind, placebo-controlled clinical trial, with 12,218 patients who were randomized to perindopril versus placebo after a 4-week run-in period. Mean follow-up was 4.2 years. The study recruited men and women aged ≥18 years without clinical evidence of heart failure and with evidence of coronary artery

disease documented by either previous MI, percutaneous or surgical coronary revascularization or angiographic evidence of ≥70% narrowing of ≥1 major coronary artery. Men were also recruited if they had a history of chest pain and a positive exercise test or regional wall motion abnormalities during stress echocardiography or nuclear scintigraphy or with transient perfusion defects during scintigraphy perfusion imaging. All participants are residents of European descent (majority Caucasian). The local ethics committees have approved EUROPA/PERGENE. A written informed consent was obtained from all participants [37].

39) PLATO - The study of Platelet Inhibition and patient outcomes

The PLATO trial was an international randomized double blind, double dummy phase III study comparing ticagrelor with clopidogrel in patients with either ST-elevation MI intended for primary PCI or with non-ST-elevation ACS, regardless if aimed for an initial invasive or non-invasive treatment strategy. A total of 18,624 patients were included. Follow up was 6 to 12 months, with a median of 9 months. The outcome events were centrally adjudicated and included cardiovascular and total death, myocardial infarction, stent thrombosis, stroke and major bleeding. DNA for genetics analyses is available from 9340 patients [38].

40) PMI - Post Myocardial Infarction Study

The Post Myocardial Infarction Study (PMI) is a cohort study of 1,063 patients with acute myocardial infarction admitted to Christchurch Hospital (New Zealand) from 1994-2001. Inclusion criteria were acute myocardial infarction (ST-elevation myocardial infarction and non-ST-elevation myocardial infarction) as defined by the presence of typical cardiac symptoms, ischaemic change on ECG in 2 or more contiguous leads and peak elevation of creatinine kinase of at least twice normal (400U/L). Anthropometric and clinical measures, including echocardiography and a large number of neurohormonal markers, were documented during the index hospital admission Clinical outcomes were recorded from the New Zealand Health Information Service over a median 9.3 years after admission (maximum 13.4 years). The study was approved by the Canterbury Ethics Committee (CTY/94/08/783/AM04) and all patients provided written informed consent. The study was registered on the Australian New Zealand Clinical Trials Registry (ACTRN12606000212550).

41) POPular – The POPular Study

POPular is a prospective, observational, single-center cohort study of 1069 (75% male, mean age 64 years) consecutive patients taking clopidogrel plus aspirin or vitamin K antagonists undergoing elective coronary stent implantation between December 2005 and December 2007. The purpose of this study was to investigate whether the level of platelet inhibition as assessed with five point-of-care platelet function assays correlates with clinical (periprocedural) outcomes such as acute myocardial infarction, death, target Vessel revascularization and/or stroke in patients undergoing elective percutaneous coronary intervention [39]. All participants are residents of European descent. The local ethics committee approved conduct of the POPular study. A written informed consent was obtained from all participants.

42) POPular Genetics – The POPular Genetics Study

POPular Genetics is a new cohort study of individuals with ST-segment elevation myocardial infarction (STEMI) undergoing primary percutaneous coronary intervention (PCI). Further details are due to be published in due course.

43) PROSPER - Prospective Study of Pravastatin in the Elderly at Risk

PROSPER was a randomized, double-blind, placebo-controlled trial of pravastatin treatment in 5,804 elderly patients with pre-existing vascular disease or at significant risk, that ran between 1997 and 1999. The patients were randomized for treatment with 40 mg pravastatin/day or placebo in 3 centers: Cork (Ireland), Glasgow (Scotland) and Leiden (the Netherlands) [40]. All participants were residents of European descent. The local ethics committees approved PROSPER. Written informed consent was obtained from all participants. For the purposes of the GENIUS study, 439 patients from PROSPER with known coronary heart disease (at baseline) who were allocated to placebo were included.

44) RISCA - Recurrence and Inflammation in the Acute Coronary Syndromes Study

The RISCA cohort consists of 1210 consecutive patients recruited from four tertiary and four Canadian community hospitals (seven in Quebec and one in New Brunswick). To be eligible, patients had to have an urgent admission to the hospital with a diagnosis of either acute MI or unstable angina. Of the 1210 patients enrolled, 1054 provided consent for genetic testing and 14 patients were excluded because of missing covariates, resulting in a final sample of 1040 patients for analysis. Follow-up occurred at 1 month via outpatient visit and at 1 year. All clinical data and events were verified by on-site visits and examination of all necessary supporting documents. Finally, all prospective and potential outcomes were centrally adjudicated independently by 2 cardiologist investigators.

45) SHEEP - Stockholm Heart Epidemiology Program

The Stockholm Heart Epidemiology Program (SHEEP) was originally designed as a case-control study aiming at studying risk factors of first time myocardial infarction. Between Jan 12, 1992 and Jan 12, 1994, all incident myocardial infarction events among men and women aged between 45 and 70 years and who were Swedish citizens residing in Stockholm county were identified. The identification of female cases continued for another year: from Jan 13, 1994 to Dec 31, 1994. The rate of study participation among 28-day survivors of myocardial infarction was 87%. The patients were mainly identified through a special organization set up at the 10 emergency hospitals within Stockholm county. Follow-up data concerning recurrent cardiovascular disease events and death up to December 31, 2012 have been extracted from national registers. Informed consent was obtained from all participants and the local ethics committee has approved the SHEEP. Details about the study design, including criteria for diagnosis of MI, have been described previously [41].

46) SMART – Second Manifestations of ARTerial disease

The Second Manifestations of ARTerial disease (SMART) study is an ongoing prospective cohort study at the University Medical Center Utrecht (UMCU) in the Netherlands. From September 1996 onwards, ≥12,000 patients were referred to the UMCU with clinically manifested vascular disease (coronary heart disease, cerebrovascular disease, peripheral arterial disease or abdominal aortic aneurysm) or vascular risk factors (dyslipidemia, hypertension or diabetes mellitus) and were asked to participate in the study. Written informed consent was obtained from all patients.

After inclusion, patients underwent a standardized vascular screening protocol consisting of a health questionnaire including medical history and risk factors, physical examination and laboratory testing. All patients are biannually asked to fill in a short questionnaire regarding hospitalization and outpatient clinical visits. If a patient reports a possible event, all available relevant data are collected and an outcome committee of three staff members assesses whether study outcomes (primary events: myocardial infarction, stroke and vascular death) occurred [42].

47) STABILITY - Stabilization of Atherosclerotic Plaque by Initiation of Darapladib Therapy trial

The STABILITY trial was an international prospective randomised, double-blind of darapladib versus placebo in patients with stable coronary heart disease and an indicator of increased risk. 15,828 patients were included. Follow-up was 3 – 5 years with a median of 3.7 years. The outcome events were centrally adjudicated and contained cardiovascular and total death, myocardial infarction and stroke. DNA for genetics analyses is available from 10,786 patients [43].

48) THI - Texgen

TexGen is a collaborative, prospective genetic registry which enrolls patients with any personal history of cardiovascular disease, who seek care at several institutions within the Texas Medical Center including the University of Texas Health Science Center, The University of Texas M.D. Anderson Cancer Center, Baylor College of Medicine and their affiliated hospitals, and the Texas Heart Institute at St. Luke's Episcopal Hospital. The registry includes patients presenting with ACS and those undergoing CABG surgery (with or without valve surgery procedures) from September 2001 through September 2008. Written informed consent was obtained from all study participants.

49) TNT - Treating to New Targets

The TNT study was a randomized, double-blind clinical trial to compare high-dose to moderate-dose atorvastatin as secondary prevention in patients with established stable CAD defined as previous MI, previous or present angina with objective evidence of atherosclerotic CAD, or a history of coronary revascularization. A total of 10,001 patients were randomized to treatment with either 10 mg of atorvastatin or 80 mg of atorvastatin daily from 256 sites in 14 countries between July 1998 and December 1999. Mean follow-up was 4.9 years. The study's primary endpoint was the occurrence of a first major cardiovascular event, defined as death from CAD, nonfatal nonprocedural-related MI, resuscitation, or fatal or nonfatal stroke. The study was performed under the terms of the Declaration of Helsinki and the study protocol was approved by the local review boards or ethics committees and all patients gave written informed consent. The genetic study was approved by the Montreal Heart Institute ethics committee [44].

50) TRIUMPH - Translational Research Investigating Underlying Disparities in Acute Myocardial Infarction Patient's Health Status

The TRIUMPH study was designed to explore racial differences in prognosis for myocardial infarction patients. Between June 1, 2005 and December 31, 2008, 31,567 patients with MI were prospectively screened of whom 6,152 had an eligible MI. Of eligible patients, 4,340 (71%) patients were enrolled into TRIUMPH from across 24 US centers with genetic data available for 2,979 (69%) of patients. Centralized follow-up interviews sought to quantify patients' post-discharge care and outcomes, with a focus on their health status (symptoms, function, and quality of life). At 1, 6 and 12 months, 23%, 27% and 24% were lost to follow-up. Vital status was available for 99% of patients at 12-months [45].

51) UCORBIO - Utrecht Coronary Biobank Study

The Utrecht Coronary Biobank Study (UCORBIO) enrolled 2,591 patients who underwent coronary angiography for any indication at the University Medical Center Utrecht (UMCU). Baseline assessment and blood sampling took place between 2011 and 2014. Patients were followed up (maximum: 3years) for the occurrence of major adverse cardiovascular events (stroke, myocardial infarction, coronary revascularization, death). The study was approved by the Ethics Committee of the UMCU and was conducted according to the Declaration of Helsinki.

UCORBIO is registered with clinicaltrials.gov (ID: NCT02304744)

52) UCP - Utrecht Cardiovascular Pharmacogenetics Study

Participants of the Utrecht Cardiovascular Pharmacogenetics (UCP) studies were enrolled through the Dutch population-based Pharmaco-Morbidity Record Linkage System (PHARMO) database. The PHARMO database links drug dispensing history from a representative sample of Dutch community pharmacies to the national registry of hospital discharge diagnoses. Several nested case-control studies to assess the interaction between SNPs, drug use and the risk of myocardial infarction were performed using UCP data [46, 47]. The UCP studies received ethical approval from the Medical Ethics Committee of the University Medical Center Utrecht, the Netherlands. A written informed consent was obtained from all participants.

53) UKB - UK Biobank

UK Biobank is a large-scale cohort study, including 502,655 participants aged between 40-69 years. Study participants were recruited from 22 recruitment centres across the United Kingdom between 2006 and 2010 [48]. A sub-sample of the cohort (N = 10,287) of European descent with coronary heart disease at baseline, phenotyped using ICD10 and OPS codes, were included in GENIUS analyses.

54) VHS - Verona Heart Study

The Verona Heart Study (VHS) is a regional survey aimed to search for new risk factors (RFs), particularly genetic RFs, for coronary artery disease (CAD) in subjects with angiographic documentation of their coronary vessels. Since 1996, the VHS has recruited over 2,500 adult patients of both sexes from those referring to the University Hospital of Verona and undergoing to coronary angiography. The VHS includes either patients with angiographically proven CAD or controls with completely normal coronary arteries, being initially submitted to coronary angiography for reasons other than CAD (mainly valvular heart disease, CAD-free group). Prospective data are collected with a median follow-up of 5 years.

55) VIVIT - Vorarlberg Institute for Vascular Investigation and Treatment (VIVIT) Study

The VIVIT-study has consecutively recruited 1,804 Caucasian patients who underwent coronary angiography for the evaluation of established or suspected CAD at the Academic Teaching Hospital Feldkirch (Austria) between September 1999 and April 2008. Patients undergoing coronary angiography for other reasons were not enrolled. In particular, no patients with acute coronary syndromes were enrolled. The ethics committee of the University of Innsbruck approved the study, and all participants gave written informed consent [49].

56) WARSAW ACS - Warsaw ACS Genetic Registry

Warsaw ACS genetic registry comprised of unselected, consecutive patients with ACS, (with STEMI and NSTEMI/UA) hospitalized in the years 2008-2011 in I Chair and Department of Cardiology, Medical University of Warsaw (Warsaw, Poland). Patients were treated according to the local hospital protocol, which was based on STEMI ESC guidelines. This involved primary percutaneous coronary intervention (pPCI) as a default treatment strategy in majority of patients.

The protocol was accepted by the Ethics Committee of the Medical University of Warsaw. Informed patient consent was obtained from all participants included in the registry. The analyzed end-point was total long-term mortality. Data concerning survival was retrieved from the local population registry run by a Government Office.

57) WTCCC - Welcome Trust Case Control Consortium CAD study

Cases were European Caucasians who had a validated history of either myocardial infarction (MI) or coronary revascularisation (coronary artery bypass surgery or percutaneous coronary angioplasty) before their 66th birthday. Recruitment was carried out on a national basis through: (i) responses to a sustained UK-wide media campaign, (ii) through responses to posters placed within hospitals and GP (family physician) surgeries throughout the UK, and (iii) in a pilot-phase contacting patients listed on computer based coronary artery disease databases, in the two lead centres (Leeds and Leicester). The recruitment period was from April 1998 to November 2003. The primary purpose was to recruit families with two or more available siblings with CAD, for a linkage study called the BHF Family Heart Study (BHF-FHS) [50]. During the course of the project we however, also recruited subjects with CAD who had a family history of premature CAD (in parents or another sibling), but in whom a further affected sib was not available, for a related project called the GRACE Study [51].

2 Supplemental Tables

Supplemental Table 1: Additional phenotypes available in GENIUS-CHD studies. X denotes availability of the relevant phenotype; ECG = electrocardiogram; MRI = magnetic resonance imaging; IMT = intima media thickening; ACE = Angiotensin Converting Enzyme; CRP = C=Reactive Protein;

Supplemental Table 2: Samples available in GENIUS-CHD studies. X denotes availability of the relevant sample type; RNA = ribonucleic acid; DNA = deoxyribonucleic acid; EDTA = Ethylenediaminetetraacetic acid

Supplemental Table 3: DNA & Genotyping platform details for GENIUS-CHD studies.

Supplemental Table 4: Subsequent events and follow up data available in GENIUS-CHD studies. X denotes availability of the relevant event/endpoint; CVD = cardiovascular disease; CHD = coronary heart disease; MI = myocardial infarction; mean and (SD) provided for follow up length.

Supplemental Tables 5 - 7: Random effects Meta-analyses estimates for each of age, sex and smoking.

Supplemental Table 1. Availability of phenotypes in studies contributing to GENIUS-CHD consortium.

Alias	Medications			Biomarkers			Bloods			Imaging			Vascular Function			Scales		
	Anti-plt	*Beta blocker*	*ACE Inhibitor*	*Hs-CRP*	*Lp(a)*	*IL-6*	*Hb*	*fasting gluc*	*ALT*	*Angiography*	*Echo*	*Cardiac MRI*	*Art stiffness*	*Carotid IMT*	*AB Index*	*Quality of Life*	*Physical activity*	*Socio economic*
4C	X	X	X	X						X	X	X				X		X
AGNES		X	X	X		X	X	X	X									
ANGES		X	X	X		X	X	X	X									
ATVB	X	X	X					X		X								
CABGenomics		X	X						X	X	X					X	X	X
CARDIOLINES	X	X	X	X	X	X	X	X	X	X	X	X	X					
CDCS	X	X	X	X	X		X	X		X	X						X	
COGEN	X	X	X	X	X		X	X	X	X	X							X
COROGENE	X	X	X	X			X		X	X	X							
CTMM	X	X	X	X		X	X	X	X	X					X			
CURE	X	X	X	X		X	X	X	X							X	X	X
EGCUT	X	X	X														X	X
EMORY	X	X	X	X		X	X		X	X	X					X	X	X
ERICO	X	X	X	X		X	X	X	X	X	X		X	X	X	X	X	X
FASTMI2005	X	X	X	X			X			X								
FINCAVAS		X	X	X		X	X	X	X	X							X	
FRISCII	X	X		X		X	X											
GENDEMIP	X	X	X	X			X	X	X									
GENEBANK	X	X	X	X	X	X	X	X		X	X						X	X
GENESIS-PRAXY	X	X	X	X	X	X	X	X		X						X	X	X
GENOCOR	X	X	X	X	X		X	X	X	X	X						X	X
GEVAMI	X	X	X	X			X	X		X	X	X					X	X
GoDARTSincident																		
GoDARTSprevalent	X	X	X	X		X		X	X									
GRACE_B	X	X	X			X												
GRACE_UK	X	X	X	X		X	X	X	X									
IDEAL	X	X	X			X	X	X	X									
INTERMOUNTAIN	X	X	X	X			X	X	X	X	X	X						
INVEST	X	X	X													X		
JUMC	X	X	X	X			X	X										
KAROLA	X	X	X	X	X		X	X		X						X	X	X

Alias	Medications			Biomarkers			Bloods			Imaging			Vascular Function			Scales		
	Anti-plt	*Beta blocker*	*ACE Inhibitor*	*Hs-CRP*	*Lp(a)*	*IL-6*	*Hb*	*fasting gluc*	*ALT*	*Angiography*	*Echo*	*Cardiac MRI*	*Art stiffness*	*Carotid IMT*	*AB Index*	*Quality of Life*	*Physical activity*	*Socio economic*
LIFE-Heart	X	X	X	X	X	X	X		X	X	X			X	X		X	
LURIC	X	X	X	X	X	X		X	X	X	X						X	
MDCS	X	X	X	X		X	X	X			X		X	X			X	X
NE_POLAND	X	X	X				X			X	X							
NEAPOLIS	X	X	X	X			X	X	X									
OHGS	X	X	X	X		X	X	X	X	X								
PERGENE	X	X	X															
PLATO	X	X		X		X	X			X						X		
PMI	X	X	X				X				X							
POPular	X	X	X	X		X	X		X	X								
POPular Genetics	X	X	X	X			X		X	X	X					X		
PROSPER	X	X	X	X	X	X		X	X									X
RISCA	X	X	X	X	X	X					X							
SHEEP	X	X		X	X	X		X								X	X	X
SMART	X	X	X	X		X	X	X	X					X	X	X	X	X
STABILITY	X	X		X		X	X											
THI	X	X	X				X	X		X								X
TNT	X	X	X		X	X	X	X	X								X	
TRIUMPH	X	X	X	X		X	X	X	X	X	X					X	X	X
UCORBIO		X	X	X		X			X	X	X					X	X	X
UCP	X	X	X	X		X	X	X	X								X	
UKB	X	X	X	X	X	X	X	X	X			X				X	X	X
VHS	X	X	X	X	X		X	X	X									
VIVIT	X	X	X	X	X			X	X	X								X
WARSAW ACS				X		X	X	X	X	X	X							
WTCC	X	X		X		X	X	X	X									

Additional phenotypes available in GENIUS-CHD studies. X denotes availability of the relevant phenotype; ECG = electrocardiogram; MRI = magnetic resonance imaging; IMT = intima media thickening; ACE = Angiotensin Converting Enzyme; CRP = C=Reactive Protein;

Supplemental Table 2. Availability of stored samples in studies contributing to GENIUS-CHD.

Alias	Blood-Edta	Serum	Plasma	RNA	DNA/ Buffy Coat	Urine
4C		X	X	X		
AGNES						
ANGES						
ATVB	X		X			
CABGenomics	X	X	X	X	X	
CARDIOLINES	X	X	X	X	X	
CDCS			X		X	
COGEN	X	X				
COROGENE	X	X	X		X	
CTMM	X		X	X	X	
CURE	X	X	X			
EGCUT	X	X	X	X	X	
EMORY		X	X	X		
ERICO		X	X	X		X
FASTMI2005		X	X			
FINCAVAS	X	X	X		X	
FRISCII	X	X	X			
GENDEMIP			X		X	
GENEBANK	X	X	X			
GENESIS-PRAXY	X	X	X			
GENOCOR	X	X	X			
GEVAMI	X		X		X	
GoDARTSincident	X	X	X		X	X
GoDARTSprevalent						
GRACE_B					X	
GRACE_UK						
IDEAL			X		X	
INTERMOUNTAIN			X		X	
INVEST						
JUMC		X	X		X	
KAROLA	X	X	X		X	
LIFE-Heart	X	X	X	X	X	
LURIC	X		X			
MDCS	X		X			
NE_POLAND	X					
NEAPOLIS	X		X		X	

**Alias**	**Blood-Edta**	**Serum**	**Plasma**	**RNA**	**DNA/ Buffy Coat**	**Urine**
OHGS		X	X		X	
PERGENE	X	X	X		X	X
PLATO		X	X			
PMI					X	
POPular	X				X	
POPular Genetics	X					
PROSPER	X		X		X	
RISCA	X		X			
SHEEP		X	X			
SMART	X	X	X		X	X
STABILITY						
THI	X	X	X			
TNT	X		X		X	
TRIUMPH	X	X	X		X	
UCORBIO		X	X		X	
UCP	X					
UKB	X	X	X	X	X	X
VHS		X	X		X	
VIVIT	X	X	X			X
WARSAW ACS	X					
WTCC		X	X			

Samples available in GENIUS-CHD studies. X denotes availability of the relevant sample type; RNA = ribonucleic acid; DNA = deoxyribonucleic acid; EDTA = Ethylenediaminetetraacetic acid

Supplemental Table 3. Genotyping and imputation of studies contributing to GENIUS-CHD consortium.

Alias	Main Genotyping platform(s)	Imputation
4C	Custom	-
AGNES	Illumina Human610k-Quad v1 array	-
ANGES	Metabochip	-
ATVB	Affymetrix 6.0 GeneChip	-
CABGenomics	Sequenom	-
CARDIOLINES		
CDCS	Sequenom and Taqman assays	-
COGEN	Human CoreExome BeadChip (illumina)	HRC v 1.1
COROGENE	Illumina Human610k-Quad	-
CTMM	Affymetrix Axiom tx	-
CURE	Taqman assay	-
EGCUT	IllumniaInfiumCoreExome-24, OmniExpress, Human370CNV, GlobalScreeningArray	1000 Genomes (Phase 3)
EMORY	Multiplex or taqman	-
ERICO	Affymetrix axion	-
FASTMI2005	Applied Biosciences Inc	-
FINCAVAS	Metabochip, n = 2278, CoreExome, n = 926	-
FRISCII		-
GENDEMIP	Taqman assay	-
GENEBANK	Affymetrix 6.0 GeneChip	-
GENESIS-PRAXY	Sequenom: Platform: iPLEX® Gold Genotyping Technology Taqman: Platform: TaqMan® Genotyping Technology (Applied Biosystems)	-
GENOCOR	High resolution melting curve analysis-LC Green and a Light Scanner (Idaho Tech)	-
GEVAMI	Illumnia Global Screening Array	HRC version r1.1 panel
GoDARTSincident	Affy; Affy1KG; Illumina; Illumina 1KG; broad	-
GoDARTSprevalent		
GRACE_B	Sequenom (734 samples)	-
GRACE_UK	Sequenom	-
IDEAL	Sequenom MassArray Maldi-TOF System; Illumina MEGA	-
INTERMOUNTAIN	Applied Biosciences Inc	-
INVEST	Taqman; Illumina IBC; Illumina OmniExpress	-
JUMC	TaqMan™ Real-Time PCR Assays (7900HT Fast Real Time System or OpenArray NT cycler)	-
KAROLA	restriction fragment length polymorphism (RFLP)	-
LIFE-Heart	Affymetrix Axiom CADLIFE (CEU+custom content) or Affymetrix Axiom CEU	IMPUTE2
LURIC	Affymetrix 6.0	IMPUTE2
MDCS	Sequenom MassArray or Illumina Omni Express Exome	-

NE_POLAND	ABI 7500 real time PCR	-
NEAPOLIS	GENOTYPING 7900HT Fast Real-Time PCR System	-
OHGS	Affymetrix Axiom	-
PERGENE	Taqman allelic discrimination assays (Applied Biosystems, Foster City, CA, USA) and Sequenom (San Diego, CA, USA) mass-spectrometric genotyping were used to genotype the selected SNPs, according to the manufacturer's protocols.	-
PLATO	Illumina HumanOmni2.5-4v1 BeadChip, Illumina Infinium HumanOmniExpressExome-8 v1	IMPUTE2
PMI	Sequenom and Taqman assays	-
POPular	TaqMan® Drug Metabolism Genotyping Assay performed on StepOnePlus™ system (Applied Biosystems)	-
POPular Genetics	TaqMan® Drug Metabolism Genotyping Assay performed on StepOnePlus™ system (ThermoFisher)	-
PROSPER	Illumina 660K quad beadchip	MACH
RISCA	iPLEX technology on a MassARRAY Compact Analyser	-
SHEEP	Illumina Cardiometabochip	-
SMART	TaqMan assay	-
STABILITY	Illumina HumanOmniExpressExome-8 v1 BeadChip	IMPUTE2
THI	Sequenom massarray system, TaqMan	-
TNT	Sequenom massarray Maldi-tof System; Illumina MEGA	-
TRIUMPH	Infinium HumanCore BeadChip array (GWAS) and the Illumina HumanExome v1.1 Analysis BeadChip array (Exome)	Mach/Minimac
UCORBIO	TaqMan assay	-
UCP	50K Illumina CARe iSelect (IBC)	-
UKB	Affymetrix1 UK BiLEVE Axiom™ Array /UK Biobank Axiom™ Array	
VHS	iPLEX MassARRAY platform, Multilocus genotyping assay by Roche	-
VIVIT	CardioMetaboChip	-
WARSAW ACS	TaqMan Assay; ABI 7500 real time PCR platform	-
WTCC	Affymetrix GeneChip® Human Mapping 500K Array Set	IMPUTE2

DNA & Genotyping platform details for GENIUS-CHD studies.

Supplemental Table 4. Subsequent events and ascertainment in studies contributing to GENIUS-CHD consortium.

Alias	All CVD	All Cause Death	CVD Death	CHD Death	CHD Death or MI	Myocardial Infarction	Revascularization	Heart Failure	Ischaemic Stroke	Any Stroke	Event Ascertainment Method
											**Linkage? Direct contact? Records? Adjudicated**
4C	X	X	X	X							Hospital records and linkage to mortality
AGNES	X	X	X			X	X	X		X	
ANGES	X	X	X	X	X	X	X	X	X	X	
ATVB	X	X	X	X	X	X	X			X	Telephone calls and analysis of clinical records
CABGenomics		X						X			
CARDIOLINES	X	X	X			X	X	X		X	
CDCS	X	X	X	X	X	X	X	X	X	X	New Zealand Health Information System
COGEN	X	X	X	X	X	X	X	X	X	X	Nationwide registries; Clinical records; Local revascularoization databases; cause of death was based on medical records or death certificates
COROGENE		X	X	X	X	X	X	X			Statistics Finland and Hospital discharge registry
CTMM	X	X	X			X					During follow-up, patients were questioned about the occurrence of cardiovascular events. Reported events were verified by an independent clinical event committee
CURE	X	X	X	X	X	X	X	X		X	Follow-up data were obtained based on linked information to the Estonian Health Insurance Fund (EHIF) database, regional hospital databases and the Estonian Causes of Death Registry
EGCUT	X	X	X	X	X	X	X	X	X	X	
EMORY	X	X	X	X	X		X	X		X	Follow-up data were collected by personnel blinded to the study data through telephone interview, chart review and query of the Social Security Death Index and State records. Medical records were accessed to validate all self-reported events.
ERICO	X	X	X	X	X	X					
FASTMI2005	X	X	X	X	X	X	X			X	Events were adjudicated by a scientific committee whose members were unaware of patients' medications and biobanking measurements
FINCAVAS	X	X	X	X	X	X	X	X	X	X	

Alias	All CVD	All Cause Death	CVD Death	CHD Death	CHD Death or MI	Myocardial Infarction	Revascularization	Heart Failure	Ischaemic Stroke	Any Stroke	Event Ascertainment Method
											**Linkage? Direct contact? Records? Adjudicated**
FRISCII	X	X	X	X	X	X	X				Clinical follow-up was performed via a visit to the outpatient clinic or by a telephone interview
GENDEMIP		X	X								population register
GENEBANK	X	X				X	X			X	
GENESIS-PRAXY	X	X	X			X	X	X		X	Through patient questionnaire
GENOCOR	X	X	X	X	X	X	X	X		X	Annual mail / telephone interview follow-up, clinical records; cause of death was based on medical records or death certificates
GEVAMI	X	X	X	X	X	X	X	X	X	X	Danish registry, questionnaires, medical records; Adjudicated
GoDARTS incident	X	X	X	X	X	X	X	X	X	X	From linked hospitalisation and death recs
GoDARTS prevalent	X	X	X	X	X	X	X	X	X	X	From linked hospitalisation and death recs
GRACE_B			X	X	X	X	X				
GRACE_UK	X	X		X	X	X	X	X		X	
IDEAL	X	X	X	X	X	X	X	X	X	X	
INTERMOUNTAIN	X	X	X	X	X	X	X	X		X	clinical referral for cardiac cath, enrollment prior to cath, blood obtained at cath
INVEST	X	X	X			X	X			X	
JUMC	X	X	X	X	X	X	X	X	X	X	phone call + medical records
KAROLA	X	X	X	X	X	X				X	Information on vital status and cause of death were retrieved from local health authorities; information on non-fatal outcomes were ascertained via questionnaires from physicians of participants
LIFE-Heart	X	X	X	X	X	X	X	X		X	Mail based follow-up, clinical records
LURIC	X	X	X	X	X	X				X	
MDCS	X	X	X	X	X			X	X	X	Nationwide registers
NE_POLAND		X									Data retrieved from the local population registry run by a Government Office.
NEAPOLIS	X	X	X	X	X	X	X	X	X	X	Clinical followup was performed via a visit to the outpatient clinic or by a telephone interview
OHGS	X	X	X	X	X	X	X			X	survey
PERGENE	X	X	X	X	X	X	X	X	X	X	
PLATO	X	X	X	X	X	X	X	X	X	X	Clinical followup was performed via a visit to the outpatient clinic or by a telephone interview

**Alias**	**All CVD**	**All Cause Death**	**CVD Death**	**CHD Death**	**CHD Death or MI**	**Myocardial Infarction**	**Revascularization**	**Heart Failure**	**Ischaemic Stroke**	**Any Stroke**	**Event Ascertainment Method**
											**Linkage? Direct contact? Records? Adjudicated**
PMI	X	X	X	X	X	X	X	X	X	X	New Zealand Health Information System
POPular	X	X	X	X	X	X	X		X	X	**clinical files and telephone contact with the patients**
POPular Genetics	X	X	X	X	X	X	X	X	X	X	**clinical files and questionnaires**
PROSPER	X	X	X	X	X	X	X	X		X	general practitioner and self-reported
RISCA	X	X	X	X	X	X	X	X		X	Hospital admission for UA or MI or post MI UA
SHEEP	X	X	X	X	X	X	X	X	X	X	Symptoms, cardiac enzymes, ECG
SMART		X				X	X		X	X	Bi-annual patient questionnaire + hospital discharge letters + ECG or brain imaging
STABILITY	X	X	X	X	X	X	X	X	X	X	Clinical followup was performed via a visit to the outpatient clinic or by a telephone interview
THI	X	X				X	X	X		X	
TNT	X	X	X	X	X	X	X	X		X	
TRIUMPH		X									The Social Security Administration Death Master File was queried to determine patients' vital status as of 12/31/2010 (http://www.ntis.gov/products/ssa-dmf.asp) and was available for all patients in this study. Of note, this query was performed prior to new restrictions and expunging of some records from the database.
UCORBIO	X	X	X			X	X	X	X		patients received questionnaires to obtain information on hospital admissions and major adverse cardiovascular events (MACE). The patients' general practitioner or the reported hospital were asked to confirm MACE. If a patient was hospitalized or dies, medical records were obtained to evaluate the relevance of the event or determine the cause of death.
UCP						X	X	X	X	X	
UKB	X	X	X	X	X	X	X	X	X	X	Electronic record linkage (HES/ONS)
VHS		X	X	X	X	X					Survival status was determined by searching in the National Population Register and by an ambulatory or telephone survey. Certification and date of death were obtained from the National Population Register. The causes of death were obtained from death certificates kept at the Italian Institute of Statistics (ISTAT). For non-fatal myocardial infarction a review of medical records has been also performed.

Alias	All CVD	All Cause Death	CVD Death	CHD Death	CHD Death or MI	Myocardial Infarction	Revascularization	Heart Failure	Ischaemic Stroke	Any Stroke	Event Ascertainment Method
											**Linkage? Direct contact? Records? Adjudicated**
VIVIT	X	X	X	X	X	X	X			X	National registry (Statistik Austria , Vienna, Austria) review of patient records
WARSAW ACS		X									Data retrieved from the local population registry run by a Government Office.
WTCC		X									

Subsequent events and follow up data available in GENIUS-CHD studies. X denotes availability of the relevant event/endpoint; CVD = cardiovascular disease; CHD = coronary heart disease; MI = myocardial infarction; mean and (SD) provided for follow up length

Supplemental Table 5: Random Effects meta-analysis estimates for age

Age	Number of events	Number of subjects	OR	2.5%	97.5%
CHD death or MI	19753	153186	1.152	1.121	1.184
MI	12569	138689	1.060	1.025	1.096
Heart Failure	8725	117811	1.295	1.247	1.346
Revascularization	25712	128444	0.985	0.967	1.004
Ischaemic Stroke	2035	59934	1.265	1.214	1.319
Any stroke	5004	136183	1.257	1.225	1.291
Any CVD	37737	138028	1.127	1.100	1.155
CHD death	10446	137325	1.368	1.327	1.411
CVD death	11272	133027	1.392	1.346	1.439
All cause death	22758	160697	1.386	1.351	1.423

Supplemental Table 6: Random Effects meta-analysis estimates for sex

Sex	Number of events	Number of subjects	OR	2.5%	97.5%
CHD death or MI	19753	153186	1.152	1.121	1.184
MI	12569	138689	1.060	1.025	1.096
Revascularization	25712	128444	0.985	0.967	1.004
Heart Failure	8725	117811	1.295	1.247	1.346
Ischaemic Stroke	2035	59934	1.265	1.214	1.319
Any stroke	5004	136183	1.257	1.225	1.291
Any CVD	37737	138028	1.127	1.100	1.155
CHD death	10446	137325	1.368	1.327	1.411
CVD death	11272	133027	1.392	1.346	1.439
All cause death	22758	160697	1.386	1.351	1.423

Supplemental Table 7: Random Effects meta-analysis estimates for smoking

Smoking	Number of events	Number of subjects	OR	2.5%	97.5%
CHD death or MI	10029	131067	1.413	1.309	1.526
MI	11859	134487	1.275	1.184	1.373
Revascularization	24783	124623	1.023	0.972	1.077
Heart Failure	8335	111961	1.207	1.104	1.318
Ischaemic Stroke	1908	56454	1.209	1.042	1.403
Any stroke	4755	132479	1.152	1.016	1.306
Any CVD	36275	132322	1.140	1.075	1.209
CHD death	18894	145617	1.315	1.229	1.407
CVD death	10754	126584	1.417	1.306	1.537
All cause death	21627	154732	1.499	1.410	1.594

Random effects Meta-analyses estimates for each of age, sex and smoking (Supp Tables 5,6,7).

3 Supplemental Figures

Supplemental Figure 1: Example QC plot for Body Mass Index

Quality control plot for BMI. Illustrative example of QC approaches to identify outliers and problems with variables explored in federated analyses. BMI is plotted showing by individual study the minimum (min); maximum (max); mean; standard deviation (sd) and inter-quartile range (iqr)

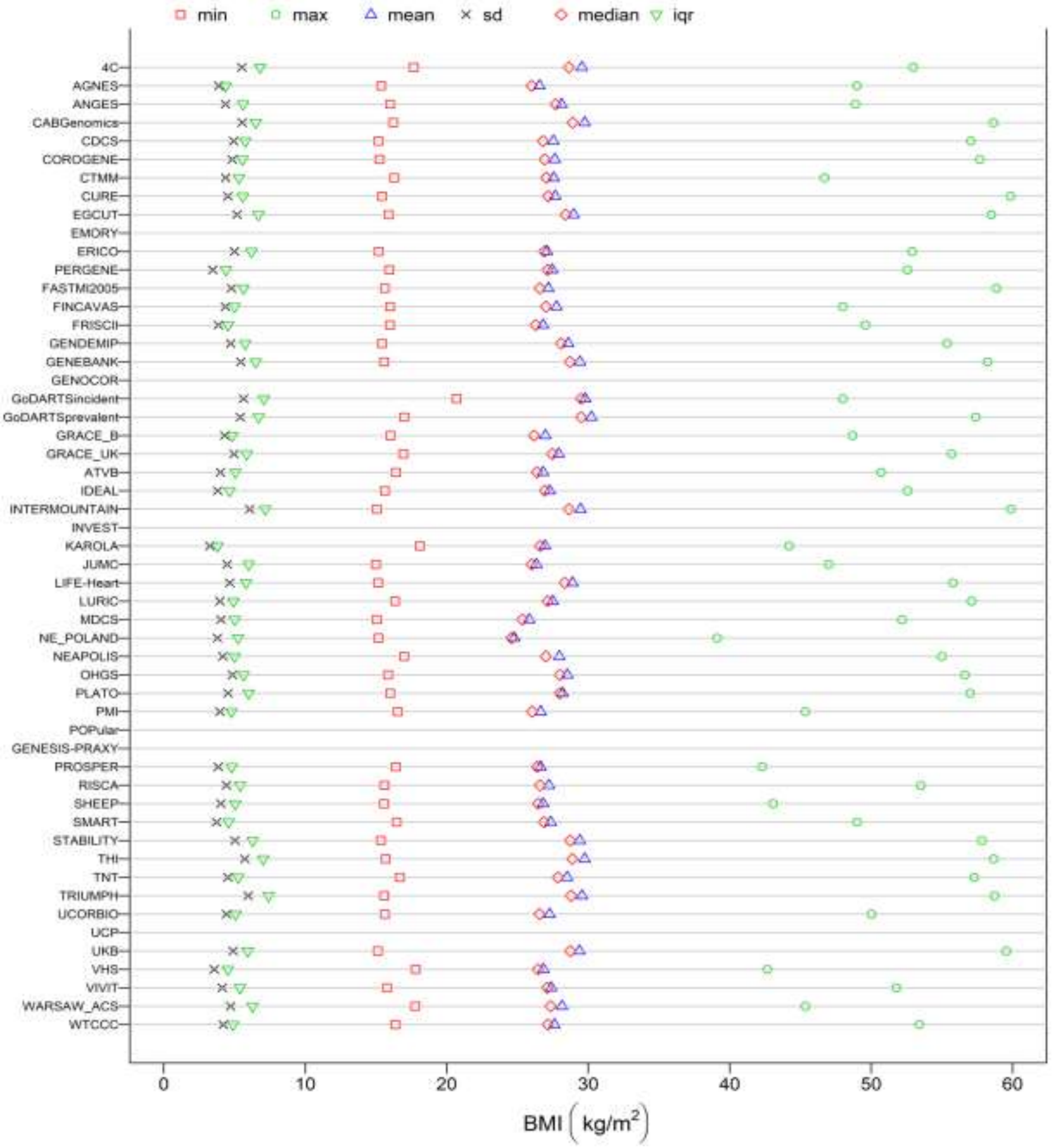

4 Supplemental References

1. Morgan TM, et al. Investigation of 95 variants identified in a genome-wide study for association with mortality after acute coronary syndrome. *BMC Medical Genetics*. 2011;12:127.
2. Reilly MP, et al. Identification of ADAMTS7 as a novel locus for coronary atherosclerosis and association of ABO with myocardial infarction in the presence of coronary atherosclerosis: two genome-wide association studies. *Lancet*. 2011;377:383-92.
3. Chan K, et al. Association between the chromosome 9p21 locus and angiographic coronary artery disease burden: a collaborative meta-analysis. *J Am Coll Cardiol*. 2013;61:957-70.
4. Gong Y, et al. Chromosome 9p21 haplotypes and prognosis in white and black patients with coronary artery disease. *Circ Cardiovasc Genet*. 2011;4:169-78.
5. R: A language and environment for statistical computing [computer program]. Vienna, Austria: R Foundation for Statistical Computing,; 2016.
6. Harrell F. *Regression Modeling Strategies: With Applications to Linear Models, Logistic Regression, and Survival Analysis*: Springer; 2001.
7. Fisher R. *Statistical methods for research workers*: Oliver and Boyd; 1934.
8. Bezzina CR, et al. Genome-wide association study identifies a susceptibility locus at 21q21 for ventricular fibrillation in acute myocardial infarction. *Nature genetics*. 2010;42:688.
9. Raitoharju E, et al. Common variation in the ADAM8 gene affects serum sADAM8 concentrations and the risk of myocardial infarction in two independent cohorts. *Atherosclerosis*. 2011;218:127-133.
10. Atherosclerosis T, et al. No evidence of association between prothrombotic gene polymorphisms and the development of acute myocardial infarction at a young age. *Circulation*. 2003;107:1117-1122.
11. Fox AA, et al. Genome-wide assessment for genetic variants associated with ventricular dysfunction after primary coronary artery bypass graft surgery. *PLoS One*. 2011;6:e24593.
12. Vaara S, et al. Cohort profile: the corogene study. *International journal of epidemiology*. 2011;41:1265-1271.
13. Hoefer IE, et al. Circulating cells as predictors of secondary manifestations of cardiovascular disease: design of the CIRCULATING CELLS study. *Clinical research in cardiology*. 2013;102:847-856.
14. Mehta S, et al. The Clopidogrel in Unstable angina to prevent Recurrent Events (CURE) trial programme; rationale, design and baseline characteristics including a meta-analysis of the effects of thienopyridines in vascular disease. *European heart journal*. 2000;21:2033-2041.
15. Investigators CiUAtPRET. Effects of clopidogrel in addition to aspirin in patients with acute coronary syndromes without ST-segment elevation. *New England Journal of Medicine*. 2001;345:494-502.
16. Goulart AC, et al. Design and baseline characteristics of a coronary heart disease prospective cohort: two-year experience from the strategy of registry of acute coronary syndrome study (ERICO study). *Clinics*. 2013;68:431-434.
17. Cambou J-P, et al. The F rench registry of A cute ST elevation or non-ST-elevation M yocardial I nfarction (FAST-MI): study design and baseline characteristics. *Archives des maladies du coeur et des vaisseaux*. 2007;100:524-534.
18. Nieminen T, et al. The Finnish Cardiovascular Study (FINCAVAS): characterising patients with high risk of cardiovascular morbidity and mortality. *BMC cardiovascular disorders*. 2006;6:9.
19. Pilote L, et al. GENESIS-PRAXY (GENdEr and Sex determInantS of cardiovascular disease: From bench to beyond-Premature Acute Coronary SYndrome). *American heart journal*. 2012;163:741-746. e2.

20. Andreassi MG, et al. Individual and summed effects of high-risk genetic polymorphisms on recurrent cardiovascular events following ischemic heart disease. *Atherosclerosis*. 2012;223:409-415.
21. Hébert HL, et al. Cohort Profile: Genetics of Diabetes Audit and Research in Tayside Scotland (GoDARTS). *International journal of epidemiology*. 2017.
22. Buysschaert I, et al. A variant at chromosome 9p21 is associated with recurrent myocardial infarction and cardiac death after acute coronary syndrome: the GRACE Genetics Study. *European heart journal*. 2010;31:1132-1141.
23. Wauters E, et al. Influence of 23 coronary artery disease variants on recurrent myocardial infarction or cardiac death: the GRACE Genetics Study. *European heart journal*. 2012;34:993-1001.
24. Pedersen TR, et al. High-dose atorvastatin vs usual-dose simvastatin for secondary prevention after myocardial infarction: the IDEAL study: a randomized controlled trial. *Jama*. 2005;294:2437-2445.
25. Horne BD, et al. Clinically feasible stratification of 1-year to 3-year post-myocardial infarction risk. *Open heart*. 2018;5:e000723.
26. Muhlestein JB, et al. Usefulness of in-hospital prescription of statin agents after angiographic diagnosis of coronary artery disease in improving continued compliance and reduced mortality. *The American journal of cardiology*. 2001;87:257-261.
27. Pepine CJ, et al. Rationale and design of the International Verapamil SR/Trandolapril Study (INVEST): an Internet-based randomized trial in coronary artery disease patients with hypertension. *Journal of the American College of Cardiology*. 1998;32:1228-1237.
28. Szczeklik W, et al. Urinary 11-dehydro-thromboxane B2 as a predictor of acute myocardial infarction outcomes: results of leukotrienes and thromboxane in myocardial infarction (LTIMI) study. *Journal of the American Heart Association*. 2016;5:e003702.
29. Devereaux P, et al. Association of postoperative high-sensitivity troponin levels with myocardial injury and 30-day mortality among patients undergoing noncardiac surgery. *Jama*. 2017;317:1642-1651.
30. Beutner F, et al. Rationale and design of the Leipzig (LIFE) Heart Study: phenotyping and cardiovascular characteristics of patients with coronary artery disease. *PLoS One*. 2011;6:e29070.
31. Pott J, et al. Genome-wide meta-analysis identifies novel loci of plaque burden in carotid artery. *Atherosclerosis*. 2017;259:32-40.
32. Winkelmann BR, et al. Rationale and design of the LURIC study-a resource for functional genomics, pharmacogenomics and long-term prognosis of cardiovascular disease. *Pharmacogenomics*. 2001;2:S1-S73.
33. Smith JG, et al. Atrial fibrillation in the Malmö Diet and Cancer study: a study of occurrence, risk factors and diagnostic validity. *European journal of epidemiology*. 2010;25:95-102.
34. Szpakowicz A, et al. The influence of renal function on the association of rs854560 polymorphism of paraoxonase 1 gene with long-term prognosis in patients after myocardial infarction. *Heart and vessels*. 2016;31:15-22.
35. Anselmi CV, et al. Routine assessment of on-clopidogrel platelet reactivity and gene polymorphisms in predicting clinical outcome following drug-eluting stent implantation in patients with stable coronary artery disease. *JACC: Cardiovascular Interventions*. 2013;6:1166-1175.
36. European Trial on Reduction of Cardiac Events with Perindopril in Stable Coronary Artery Disease Investigators. Efficacy of perindopril in reduction of cardiovascular events among patients with stable coronary artery disease: randomised, double-blind, placebo-controlled, multicentre trial (the EUROPA study). *The Lancet*. 2003;362:782-788.
37. Brugts JJ, et al. Genetic determinants of treatment benefit of the angiotensin-converting enzyme-inhibitor perindopril in patients with stable coronary artery disease. *European heart journal*. 2010;31:1854-1864.

38. James S, et al. Comparison of ticagrelor, the first reversible oral P2Y12 receptor antagonist, with clopidogrel in patients with acute coronary syndromes: rationale, design, and baseline characteristics of the PLATelet inhibition and patient Outcomes (PLATO) trial. *American heart journal*. 2009;157:599-605.
39. Breet NJ, et al. Comparison of platelet function tests in predicting clinical outcome in patients undergoing coronary stent implantation. *Jama*. 2010;303:754-762.
40. Shepherd J, et al. The design of a prospective study of pravastatin in the elderly at risk (PROSPER). *American Journal of Cardiology*. 1999;84:1192-1197.
41. Reuterwall C, et al. Higher relative, but lower absolute risks of myocardial infarction in women than in men: analysis of some major risk factors in the S heep study. *Journal of internal medicine*. 1999;246:161-174.
42. Simons PCG, et al. Second manifestations of ARTerial disease (SMART) study: rationale and design. *European journal of epidemiology*. 1999;15:773-781.
43. Stability Investigators. Darapladib for preventing ischemic events in stable coronary heart disease. *New England Journal of Medicine*. 2014;370:1702-1711.
44. LaRosa JC, et al. Intensive lipid lowering with atorvastatin in patients with stable coronary disease. *New England Journal of Medicine*. 2005;352:1425-1435.
45. Arnold SV, et al. Translational research investigating underlying disparities in acute myocardial infarction patients' health status (triumph): Design and rationale of a prospective multicenter registry. *Circulation: Cardiovascular Quality and Outcomes*. 2011;4:467-476.
46. Kröger E, et al. Treatment with rivastigmine or galantamine and risk of urinary incontinence: results from a Dutch database study. *Pharmacoepidemiology and drug safety*. 2015;24:276-285.
47. Ahmadizar F, et al. Cardiovascular medication use and cardiovascular disease in children and adolescents with type 1 diabetes: a population-based cohort study. *Pediatric diabetes*. 2016;17:433-440.
48. Bycroft C, et al. The UK Biobank resource with deep phenotyping and genomic data. *Nature*. 2018;562:203.
49. Muendlein A, et al. Evaluation of the prevalence and prospective clinical impact of the JAK2 V617F mutation in coronary patients. *American journal of hematology*. 2014;89:295-301.
50. Group BFHSR. A genomewide linkage study of 1,933 families affected by premature coronary artery disease: The British Heart Foundation (BHF) Family Heart Study. *The American Journal of Human Genetics*. 2005;77:1011-1020.
51. Samani NJ, et al. Genomewide association analysis of coronary artery disease. *New England Journal of Medicine*. 2007;357:443-453.
