# Supplementary material for: Subsequent Event Risk in Individuals With Established Coronary Heart Disease: Design and Rationale of the GENIUS-CHD Consortium
Source: Circ Genom Precis Med. 2019 Apr 16;12(4):e002470. doi: 10.1161/CIRCGEN.119.002470 (PMC6629546; doi:10.1161/CIRCGEN.119.002470)
Supplement: Supplementary file 2 [file hcg-12-e002470-s002.pdf]

Subsequent Event Risk in Individuals with Established Coronary Heart Disease: Design and Rationale of the GENIUS-CHD Consortium

Running title: *Patel, Tragante, Schmidt et al.; Design & Rationale of GENIUS-CHD*

Riyaz Patel, MD[1,2]*; Vinicius Tragante, PhD[3]*; Amand F. Schmidt, PhD[1,3]*; Raymond O. McCubrey, MS[4]; Michael V. Holmes, MD, PhD[5-7]; Laurence J. Howe, PhD[1]; Kenan Direk, PhD[1]; Axel Åkerblom, MD, PhD[8,9]; Karin Leander, PhD[10]; Salim S. Virani, MD, PhD[11,12]; Karol A. Kaminski, MD, PhD[13,14]; Jochen D. Muehlschlegel, MD, MMSc[15,16]; Hooman Allayee, PhD[17]; Peter Almgren, MSc[18]; Maris Alver, MSc[19,20]; Ekaterina V. Baranova, MSc[21]; Hassan Behloui, PhD[22]; Bram Boeckx, PhD[23,24]; Peter S. Braund, PhD[25,26]; Lutz P. Breitling, MD[27]; Graciela Delgado, MSc[28]; Nubia E. Duarte, PhD[29]; Marie-Pierre Dubé, PhD[30,31]; Line Dufresne, MSc[22,32]; Niclas Eriksson, PhD[8]; Luisa Foco, PhD[33]; Markus Scholz, PhD[34,35]; Crystel M. Gijsberts, MD, PhD[36]; Charlotte Glinge, MD[37,38]; Yan Gong, PhD[39]; Jaana Hartiala, PhD[17,40]; Mahyar Heydarpour, PhD[15,16]; Jaroslav A. Hubacek, DSc[41]; Marcus Kleber, PhD[28]; Daniel Kofink, PhD[3]; Salma Kotti, PharmD, PhD[42]; Pekka Kuukasjärvi, PhD[43]; Vei-Vei Lee, MS[44]; Andreas Leiherer, PhD[45-47]; Petra A. Lenzini, MS[48]; Daniel Levin, PhD[49]; Leo-Pekka Lyytikäinen, MD[50,51]; Nicola Martinelli, MD, PhD[52]; Ute Mons, PhD[27]; Christopher P. Nelson, PhD[25,26]; Kjell Nikus, PhD[53,54]; Anna P. Pilbrow, PhD[55]; Rafal Ploski, MD, PhD[56]; Yan V. Sun, PhD[57,58]; Michael W.T. Tanck, PhD[59]; W.H.Wilson Tang, MD[60,61]; Stella Trompet, PhD[62,63]; Sander W. van der Laan, PhD[64]; Jessica Van Setten, PhD[65]; Ragnar O. Vilmundarson, MSc[66,67]; Chiara Viviani Anselmi, PhD[68]; Efthymia Vlachopoulou, PhD[69]; Lawien Al Ali, MD[70]; Eric Boerwinkle, PhD[71]; Carlo Briguori, MD, PhD[72]; John F. Carlquist, PhD[4,73]; Kathryn F. Carruthers, MPhil[74]; Gavino Casu, MD[75]; John Deanfield, MD[1,2]; Panos Deloukas, PhD[76,77]; Frank Dudbridge, PhD[78]; Thomas Engstrøm, MD, PhD[79,80]; Natalie Fitzpatrick, MSc[81]; Kim Fox, MD, PhD[82]; Bruna Gigante, PhD[10]; Stefan James, MD, PhD[8,83]; Marja-Liisa Lokki, PhD[69], Paulo A. Lotufo, MD, PhD[84]; Nicola Marziliano, PhD[85]; Ify R. Mordi, MD[49]; Joseph B. Muhlestein MD[4,73]; Christopher Newton-Cheh, MD[86]; Jan Pitha, PhD[41]; Christoph H. Saely, MD[45,46,87]; Ayman Samman-Tahhan, MD[88]; Pratik B. Sandesara, MD[88]; Andrej Teren, MD[35,89], Adam Timmis, MD[81,90]; Frans Van de Werf, PhD[91]; Els Wauters, PhD[92]; Arthur A.M. Wilde, MD, PhD[93,94]; Ian Ford, MD, PhD[95]; David J. Stott, MD[96]; Ale Algra, MD[97]; Maria G. Andreassi, PhD[98], Diego Ardissino, MD[99], Benoit J. Arsenault, PhD[100,101]; Christie M. Ballantyne, MD[12]; Thomas O. Bergmeijer, MD[102]; Connie R. Bezzina, PhD[93]; Simon C. Body, MBChB, MPH[16,103]; Eric H. Boersma, MD, PhD[104,105]; Peter Bogaty, MD[106]; Michiel Bots, MD[107]; Hermann Brenner, MD, PhD[27,108]; Jasper J. Brugts, MD, PhD[104]; Ralph Burkhardt, MD[35,109]; Clara Carpeggiani, MD[98]; Gianluigi Condorelli, MD, PhD[68,110]; Rhonda M. Cooper-DeHoff, PharmD[39,111]; Sharon Cresci, MD[48,112]; Nicolas Danchin, MD, PhD[113,114]; Ulf de Faire, PhD[10]; Robert N. Doughty, MD[115]; Heinz Drexel, MD[45,46,116]; James C. Engert, PhD[32,117,118]; Keith A.A. Fox, MD, PhD[119]; Domenico Girelli, MD, PhD[52]; Diederick E. Grobbee, MD, PhD[107]; Emil Hagström, MD, PhD[9,120]; Stanley L. Hazen, MD, PhD[60,121]; Claes Held, MD, PhD[8,9]; Harry Hemingway, MD, PhD[81]; Imo E. Hoefer, MD, PhD[122]; G. Kees Hovingh, MD, PhD[123]; Reza Jabbari, MD, PhD[124]; Julie A. Johnson, PharmD[39,125]; J. Wouter Jukema, MD, PhD[63,126,127]; Marcin P. Kaczor, MD, PhD[128]; Mika Kähönen, PhD[129,130]; Jiri Kettner, PhD[131]; Marek Kiliszek, MD, PhD[132]; Olaf H. Klungel, PharmD, PhD[21]; Bo Lagerqvist, MD, PhD[8,83]; Diether Lambrechts, PhD[23,24]; Jari O. Laurikka, PhD[133,134]; Terho Lehtimäki, PhD[50,51]; Daniel Lindholm, MD, PhD[8,9]; B. K. Mahmoodi, MD, PhD[102]; Anke H. Maitland-van der Zee, PharmD, PhD[21,135]; Ruth McPherson, MD, PhD[66,136]; Olle Melander, MD, PhD[18,137]; Andres Metspalu, MD, PhD[19,20]; Anna Niemcunowicz-Janica, MD, PhD[138]; Oliviero Olivieri, MD[52]; Grzegorz Opolski, MD, PhD[139]; Colin N. Palmer, PhD[140]; Gerard Pasterkamp, MD, PhD[141]; Carl J. Pepine, MD[125]; Alexandre C. Pereira, MD, PhD[29]; Louise Pilote, MD[22,142]; Arshed A. Quyyumi, MD[88]; A. Mark Richards, MD, PhD[55,143]; Marek Sanak, MD, PhD[128]; Agneta Siegbahn, MD, PhD[8,144]; Tabassome Simon, MD, PhD[145,146]; Juha Sinisalo, MD, PhD[147]; J. Gustav Smith, MD, PhD[148-150]; John A. Spertus, MD, MPH[151,152]; Steen Stender, MD, DSc[153]; Alexandre F.R. Stewart, PhD[66,67]; Wojciech Szczeklik, MD, PhD[128]; Anna Szpakowicz, MD, PhD[14]; Jean-Claude Tardif, MD[30,31]; Jurriën M. ten Berg, MD, PhD[102]; Jacob Tfelt-Hansen, MD, DMSc[154]; George Thanassoulis, MD[32,22,118]; Joachim Thiery, MD[35,155]; Christian Torp-Pedrsen, MD, DSc[156,157]; Yolanda van der Graaf, MD[107]; Frank L.J. Visseren, MD[158]; Johannes Waltenberger[159]; Peter E. Weeke, MD, PhD[160]; Pim Van der Harst, MD, PhD[70]; Chim C. Lang MD[49]; Naveed Sattar, PhD[96]; Vicky A. Cameron, PhD[55]; Jeffrey L. Anderson, MD[4,73]; James M. Brophy, MD[22,142]; Guillaume Pare, MD[161,162]; Benjamin D. Horne, PhD, MPH[4,163]; Winfried März, MD[28,164,165]; Lars Wallentin, MD, PhD[8,83]; Nilesh J. Samani, MD, PhD[25,26]†; Aroon D. Hingorani, MD, PhD[1]†; Folkert W. Asselbergs, MD, PhD[1,3,166]†

[1]Insti of Cardiovascular Science, Faculty of Population Health Science, [81]Insti of Health Informatics, Faculty of Population Health Science, Univ College London; [2]Bart's Heart Centre, St Bartholomew's Hospital; [90]Bart's Heart Centre, St Bartholomew's Hospital, London; [3]Dept of Cardiology, Division Heart & Lungs, UMC Utrecht, Utrecht, Netherlands; [4]Intermountain Heart Insti, Intermountain Medical Ctr, Salt Lake City, UT; [5]Clinical Trial Service Unit & Epidemiological Studies Unit, Nuffield Dept of Population Health, [6]Medical Research Council Population Health Research Unit, Univ of Oxford; [7]National Insti for Health Research Oxford Biomedical Research Centre, Oxford Univ Hospital, Oxford, UK; [8]Uppsala Clinical Research Ctr; [9]Dept of Medical Sciences, Cardiology, [83]Dept of Medical Sciences, Cardiology, [144]Dept of Medical Sciences, Clinical Chemistry, Uppsala Univ, Uppsala; [10]Insti of Environmental Medicine, Karolinska Instit, Stockholm, Sweden; [11]Section of Cardiology, Michael E. DeBakey Veterans Affairs Medical Ctr; [12]Section of Cardiovascular Research, Dept of Medicine, Baylor College of Medicine, Houston, TX; [13]Dept of Population Medicine & Civilization Disease Prevention, [14]Dept of Cardiology; Medical Univ of Bialystok, Białystok, Poland; [15]Dept of Anesthesiology, Perioperative & Pain Medicine, Brigham & Women's Hospital; [16]Harvard Medical School, Boston, MA; [17]Depts of Preventive Medicine & Biochemistry & Molecular Medicine, [40]Insti for Genetic Medicine, Keck School of Medicine of USC, Los Angeles, CA; [18]Dept of Clinical Sciences, Lund Univ, Malmö, Sweden; [19]Estonian Genome Ctr, Insti of Genomics, [20]Dept of Biotechnology, Insti of Molecular & Cell Biology, Univ of Tartu, Tartu, Estonia; [21]Division of Pharmacoepidemiology & Clinical Pharmacology, Utrecht Univ, Utrecht, Netherlands; [22]Centre for Outcomes Research & Evaluation, Research Insti of the McGill Univ Health Centre; [117]Research Insti of the McGill Univ Health Centre, Montreal, Quebec, Canada; [23]Laboratory for Translational Genetics, Dept of

Human Genetics, [91]Dept of Cardiovascular Sciences, KU Leuven; [24] Laboratory for Translational Genetics, VIB Ctr for Cancer Biology, VIB, Belgium; [25]Dept of Cardiovascular Sciences, Univ of Leicester, BHF Cardiovascular Research Centre, [26]NIHR Leicester Biomedical Research Centre, Glenfield Hospital, Leicester, UK; [27]Division of Clinical Epidemiology & Aging Research, German Cancer Research Ctr (DKFZ), Heidelberg; [28]Vth Dept of Medicine, Medical Faculty Mannheim, Heidelberg Univ, Mannheim, Germany; [29]Heart Insti, Univ of Sao Paulo, São Paulo, Brazil; [30]Montreal Heart Insti; [31]Université de Montréal, Faculty of Medicine; [32]Preventive & Genomic Cardiology, McGill Univ Health Ctr, Montreal, QC, Canada; [33]Insti for Biomedicine, Eurac Research, Affiliated Insti of the Univ of Lübeck, Bolzano, Italy; [34]Insti for Medical Informatics, Statistics & Epidemiology, [35]LIFE Research Ctr for Civilization Diseases, Univ of Leipzig, Leipzig, Germany; [36]Laboratory of Experimental Cardiology, [122]Dept of Clinical Chemistry and Hematology, UMC Utrecht, Utrecht, Netherlands; [37]Dept of Cardiology, The Heart Centre, Copenhagen Univ Hospital, Rigshospitalet; [79]The Dept of Cardiology, The Heart Centre, Copenhagen Univ Hospital, Rigshospitalet; [124]Dept of Cardiology, The Heart Centre, Copenhagen Univ Hospital, Rigshospitalet, Copenhagen, Denmark; [38]Amsterdam UMC, Univ of Amsterdam, Clinical & Experimental Cardiology, Amsterdam Cardiovascular Sciences, AMC Heart Ctr; [93]Amsterdam UMC, Univ of Amsterdam, Clinical & Experimental Cardiology, Amsterdam Cardiovascular Sciences, AMC Heart Ctr, the Netherlands; [39]Dept of Pharmacotherapy & Translational Research & Ctr for Pharmacogenomics, [111]College of Medicine, Division of Cardiovascular Medicine, [125]College of Medicine, Division of Cardiovascular Medicine, Univ of Florida, Gainesville FL; [41]Centre for Experimental Medicine, Institut for Clinical & Experimental Medicine, Prague, Czech Republic; [42] Assistance Publique-Hôpitaux de Paris (APHP), Dept of Clinical Pharmacology, Platform of Clinical Research of East Paris (URCEST-CRCEST-CRB HUEP-UPMC), Paris, France; [43]Dept of Cardio-Thoracic Surgery, [51]Dept of Clinical Chemistry, [54]Dept of Cardiology, [130]Dept of Clinical Physiology, [134]Dept of Cardio-Thoracic Surgery, Finnish Cardiovascular Research Ctr - Tampere, Faculty of Medicine & Life Sciences, Univ of Tampere, Tampere, Finland; [44]Dept of Biostatistics & Epidemiology, Texas Heart Insti, Houston, TX; [45]Vorarlberg Insti for Vascular Investigation & Treatment (VIVIT), Feldkirch, Austria; [46]Private Univ of the Principality of Liechtenstein, Triesen, Liechtenstein; [47]Medical Central Laboratories, Feldkirch, Austria; [48]Dept of Genetics, Statistical Genomics Division, [112]Dept of Medicine, Cardiovascular Division, Washington Univ School of Medicine, Saint Louis, MO; [49]Division of Molecular & Clinical Medicine, School of Medicine, Univ of Dundee, Dundee, Scotland, UK; [50]Dept of Clinical Chemistry, Fimlab Laboratories, Tampere, Finland; [52] Dept of Medicine, Univ of Verona, Verona, Italy; [53]Dept of Cardiology, Heart Ctr, [129]Dept of Clinical Physiology, [133]Dept of Cardio-Thoracic Surgery, Heart Ctr, Tampere Univ Hospital, Tampere, Finland; [55]The Christchurch Heart Insti, Univ of Otago Christchurch, Christchurch, New Zealand; [56]Dept of Medical Genetics, [139]1st Chair and Dept of Cardiology, Medical Univ of Warsaw, Warsaw, Poland; [57]Dept of Epidemiology, Emory Univ Rollins School of Public Health; [58]Dept of Biomedical Informatics, [88]Division of Cardiology, Dept of Medicine, Emory Clinical Cardiovascular Research Insti, Emory Univ School of Medicine, Atlanta, GA; [60]Dept of Cellular & Molecular Medicine, Lerner Research Insti, [61]Dept of Cardiovascular Medicine, Heart & Vascular Insti, and Ctr for Clinical Genomics, [121]Dept of Cardiovascular Medicine, Heart and Vascular Insti & Ctr for Microbiome and Human Health, Cleveland Clinic, OH; [62]Section of Gerontology & Geriatrics, Dept of Internal Medicine, [63]Dept of Cardiology, Leiden Univ Medical Ctr, Leiden; [65] Dept of Cardiology, Division Heart and Lungs, UMC Utrecht, Univ of Utrecht, Utrecht, Netherlands; [66] Ruddy Canadian Cardiovascular Genetics Centre, Univ of Ottawa Heart Insti; [67]Dept of Biochemistry, Microbiology & Immunology, [136]Depts of Medicine & Biochemistry, Microbiology and Immunology, Univ of Ottawa, Ottawa, Ontario, Canada; [68]Dept of Cardiovascular Medicine, Humanitas Clinical & Research Ctr, Milan, Italy; [69]Transplantation Laboratory, [147]Heart and Lung Ctr Helsinki Univ Hospital & Univ of Helsinki, Helsinki, Finland; [70]Univ Medical Ctr, Univ of Groningen, Groningen, The Netherlands; [71]Univ of Texas School of Public Health, Houston, TX; [72]Clinica Mediterranea, Naples, Italy; [73]Cardiology Division, Dept of Internal Medicine, [163]Dept of Biomedical Informatics, Univ of Utah, Salt Lake City, UT; [74]QMRI, Cardiovascular Sciences, [119]Emeritus Professor of Cardiology, Univ of Edinburgh, Edinburgh, UK; [75]ATS Sardegna, ASSL Nuoro - Ospedale San Francesco, Nuoro, Italy; [76]William Harvey Research Insti, Barts & the London Medical School, [77]Centre for Genomic Health, Queen Mary Univ of London, London; [78]Dept of Health Sciences, Univ of Leicester, Leicester, UK; [80]Dept of Cardiology, Univ of Lund, Lund, Sweden; [82]National Heart and Lung Insti, Imperial College & Insti of Cardiovascular Medicine and Science, Royal Brompton Hospital, London, UK; [84]Centro de Pesquisa Clinica, Hospital Universitario, Universidade de Sao Paulo, São Paulo, Brazil; [85]ATS Sardegna, ASL 3 Nuoro, Nuoro, Italy; [86]Cardiovascular Research Ctr & Ctr for Human Genetic Research, Massachusetts General Hospital, Boston, MA; Program in Medical & Population Genetics, Broad Insti, Cambridge, MA; [87]Dept of Medicine & Cardiology, Academic Teaching Hospital Feldkirch, Feldkirch, Austria; [89]Heart Ctr Leipzig, Leipzig, Germany; [92]Respiratory Oncology Unit, Dept of Respiratory Medicine, Univ Hospitals KU Leuven, Leuven, Belgium; [94]Princess Al-Jawhara Al-Brahim Centre of Excellence in Research of Hereditary Disorders, Jeddah, Saudi Arabia; [95] Robertson Ctr for Biostatistics, Univ of Glasgow; [96] Insti of Cardiovascular & Medical Sciences, Univ of Glasgow, Glasgow, UK; [64]Laboratory of Clinical Chemistry & Hematology, Division Laboratories, Pharmacy, and Biomedical Genetics, [97]Dept of Neurology & Neurosurgery, Brain Centre Rudolf Magnus and Julius Ctr for Health Sciences and Primary Care, [107]Julius Ctr for Health Sciences & Primary Care, [158]Dept of Vascular Medicine, UMC Utrecht, Utrecht Univ, Utrecht, The Netherlands; [98] CNR Insti of Clinical Physiology, Pisa; [99] Cardiology Dept, Parma Univ Hospital, Parma, Italy; [100]Centre de recherche de l'Institut Universitaire de cardiologie et de pneumologie de Québec; [101]Dept of Medicine, Faculty of Medicine, Université Laval, Québec, Canada; [102]St. Antonius Hospital, Dept Cardiology, Nieuwegein, The Netherlands; [103] Dept of Anesthesia, Critical Care & Pain Medicine, Beth Israel Deaconess Medical Ctr, Boston, MA; [104]Dept of Cardiology, Erasmus MC, Thoraxcenter; [105]Cardiovascular Research School, Erasmus Medical Ctr (COEUR), Rotterdam, the Netherlands; [106]Laval Univ, Institut universitaire de cardiologie et de pneumologie de Québec, Québec City, Québec, Canada; [108]Network Aging Research (NAR), Univ of Heidelberg, Heidelberg; [109]Insti of Clinical Chemistry & Laboratory Medicine, Univ Hospital Regensburg, Germany; [110]Dept of Biomedical Sciences, Humanitas Univ, Milan, Italy; [113]Assistance Publique-Hôpitaux de Paris (APHP), Dept of Cardiology, Hôpital Européen Georges Pompidou & FACT (french Alliance for cardiovascular trials); Université Paris Descartes; [114]Université Paris-Descartes, Paris, France; [115]Heart Health Research Group, Univ of Auckland, Auckland, New Zealand; [116]Drexel Univ College of Medicine, Philadelphia PA; [118]Division of Cardiology, Dept of Medicine, Royal Victoria Hospital, McGill Univ Health Centre, Montreal, Quebec, Canada; [120]Uppsala Univ, Dept of Cardiology, Uppsala, Sweden & Uppsala Clinical Research Ctr, Uppsala, Sweden; [123]Dept of Vascular Medicine, Academic Medical Ctr, Amsterdam; [126]Einthoven Laboratory for Experimental Vascular Medicine, LUMC, Leiden; [127]Interuniversity Cardiology Insti of the Netherlands, Utrecht, The Netherlands; [128]Dept of Internal Medicine, Jagiellonian Univ Medical College, Kraków, Poland; [131]Cardiology Centre, Insti for Clinical & Experimental Medicine, Prague, Czech Republic; [132]Dept of Cardiology & Internal Diseases, Military Insti of Medicine, Warsaw, Poland; [135]Dept of Respiratory Medicine, Academic Medical Ctr, Univ of Amsterdam; [137]Dept of Internal Medicine, Skåne Univ Hospital, Malmö, Sweden; [138]Dept of Forensic Medicine; Medical Univ of Bialystok; [140] Pat Macpherson Centre for Pharmacogenetics & Pharmacogenomics, Division of Molecular & Clinical Medicine, Ninewells Hospital & Medical School, Dundee; [141]Dept of Clinical Chemistry, UMC Utrecht, the Netherlands; [142]Dept of Medicine, McGill Univ Health Centre, Montreal, Québec, Canada; [143]Cardiovascular Research Insti, National Univ of Singapore, Singapore; [145]Assistance Publique-Hôpitaux de Paris (APHP), Dept of Clinical Pharmacology, Platform of Clinical Research of East Paris (URCEST-CRCEST-CRB HUEP-UPMC), FACT (French Alliance for Cardiovascular trials); Sorbonne Université; [146]Paris-Sorbonne Univ, UPMC-Site St Antoine, Paris, France; [148]Dept of Cardiology, Clinical Sciences, Lund Univ & Skåne Univ Hospital; [149]Wallenberg Ctr for Molecular Medicine & Lund Univ Diabetes Ctr, Lund Univ, Lund, Sweden; [150]Program in Medical & Population Genetics, Broad Insti, Cambridge, MA; [151]Saint Luke's Mid America Heart Insti, Univ of Missouri-Kansas City; [152]Saint Luke's Mid America Heart Insti Kansas City, MO; [153]Dept of Clinical Biochemistry, Copenhagen Univ Hospital, Gentofte; [154]Dept of Cardiology, The Heart Centre, Copenhagen Univ Hospital, Rigshospitalet & Dept of Forensic Medicine, Faculty of Medical Sciences, Univ of Copenhagen, Copenhagen, Denmark; [155]Insti of Laboratory Medicine, Clinical Chemistry & Molecular Diagnostics, Univ Hospital, Leipzig, Germany; [156]Unit of Epidemiology & Biostatistics, [157]Dept of Health Science & Technology, Aalborg Univ Hospital, Aalborg, Denmark; [159]Dept of Cardiovascular Medicine, Univ of Münster, Münster, Germany; [160]Dept of Cardiology, Herlev & Gentofte Hospital, Hellerup, Denmark; [161]McMaster Univ, Dept of Pathology & Molecular Medicine; [162]Population Health Research Insti, Hamilton, ON, Canada; [164]Synlab Academy, Synlab Holding Deutschland GmbH, Mannheim, Germany; [165]Clinical Insti of Medical & Chemical Laboratory Diagnostics, Medical Univ of Graz, Graz, Austria; [166]Durrer Centre of Cardiogenetic Research, ICIN-Netherlands Heart Insti, Utrecht, Netherlands

*****contributed equally / †contributed equally

Correspondence:

Riyaz S. Patel
Institute of Cardiovascular Sciences
University College London
222 Euston Rd.
London, NW1 2DA
United Kingdom
Tel: 020 3549 5332
E-mail: Riyaz.Patel@ucl.ac.uk

Folkert W. Asselbergs
Department of Cardiology,
Division of Heart & Lungs,
University Medical Center Utrecht
3508GA, Utrecht
Netherlands
Tel: +31 887553358
E-mail: F.W.Asselbergs@umcutrecht.nl

Journal Subject Terms: Genetic, Association Studies; Cardiovascular Disease; Epidemiology; Secondary Prevention; Biomarkers

Abstract:

Background: The "**GEN**et**I**cs of s**U**b**S**equent **C**oronary **H**eart **D**isease" (GENIUS-CHD) consortium was established to facilitate discovery and validation of genetic variants and biomarkers for risk of subsequent CHD events, in individuals with established CHD.
Methods: The consortium currently includes 57 studies from 18 countries, recruiting 185,614 participants with either acute coronary syndrome, stable CHD or a mixture of both at baseline. All studies collected biological samples and followed-up study participants prospectively for subsequent events.
Results: Enrolment into the individual studies took place between 1985 to present day with duration of follow up ranging from 9 months to 15 years. Within each study, participants with CHD are predominantly of self-reported European descent (38%-100%), mostly male (44%-91%) with mean ages at recruitment ranging from 40 to 75 years. Initial feasibility analyses, using a federated analysis approach, yielded expected associations between age (HR 1.15 95% CI 1.14-1.16) per 5-year increase, male sex (HR 1.17, 95% CI 1.13-1.21) and smoking (HR 1.43, 95% CI 1.35-1.51) with risk of subsequent CHD death or myocardial infarction, and differing associations with other individual and composite cardiovascular endpoints.
Conclusions: GENIUS-CHD is a global collaboration seeking to elucidate genetic and non-genetic determinants of subsequent event risk in individuals with established CHD, in order to improve residual risk prediction and identify novel drug targets for secondary prevention. Initial analyses demonstrate the feasibility and reliability of a federated analysis approach. The consortium now plans to initiate and test novel hypotheses as well as supporting replication and validation analyses for other investigators.

Key words: coronary artery disease; genetics, association studies; residual risk; secondary prevention; recurrent event; myocardial infarction; prognosis

American Heart Association
Circulation: Genomic and Precision Medicine

Introduction

Major public health initiatives and policy changes, along with advances in drug and interventional therapies have significantly reduced cardiovascular morbidity and mortality in most high-income countries.[1-3] However, the improved survival rates following an initial presentation with coronary heart disease (CHD) has, paradoxically, led to a growing number of patients living with established CHD (e.g 16M in the USA, 3M in the UK)[4 5] who remain at substantially high risk of subsequent cardiovascular events. These include myocardial infarction (MI), repeated revascularizations, but also heart failure (HF), stroke and sudden death.[4]

Despite a large body of knowledge on the pathophysiology of *first* CHD events in general populations,[6, 7] little is known about factors that influence disease progression or *subsequent* events in patients with established CHD, beyond those consequent to the acute index event in the short term (such as biomarkers of myocardial dysfunction or necrosis, left ventricular function or arrhythmia).[8] As a result, although guidelines and treatment thresholds have progressively evolved over the last two decades, the targeted risk factors *per se* have remained largely unaltered.[9] Novel therapies beyond lipid lowering, anti-platelet agents, and drugs recommended for high blood pressure and heart failure have been slow to emerge. Importantly, multiple novel and existing agents (e.g darapladib, varespladib, , and folic acid) have failed in very late stage clinical development despite promising observational data.[10, 11 12, 13] In contrast, some traditional risk factors, such as obesity, which show robust associations with initial CHD onset,[14] continue to show inverse or null associations with subsequent events once CHD has developed.[15]

Ultimately, the high (residual) risk in individuals with existing CHD despite optimal contemporary therapy emphasizes the need for studying risk of subsequent events and their related causal pathways. For example, in the intervention arm of the IMPROVE-It study, despite

American Heart Association
Circulation: Genomic and Precision Medicine

simvastatin and ezetimibe treatment following an acute coronary syndrome, at 7 years, almost a third of participants experienced the primary endpoint (a composite of cardiovascular death, major coronary event, coronary revascularization or nonfatal stroke).[16] Similarly, in the FOURIER trial, almost 10% of patients with established but stable CVD, experienced an event at 2.2 years despite high intensity statin and PCSK9 inhibition, with achieved median LDL-C levels of 30mg/dL.[17] These data point to the existence of risk factors beyond traditional ones such as LDL-C, and the need to elucidate their related causal pathways.[18] By studying those with established CHD at high risk of subsequent events, we plan to gain novel insights into other drivers of atherosclerosis or features that identify patients who may benefit most from novel therapies.[9] Genetic and biomarker studies in these individuals may help identify novel molecular pathways and future drug targets with the goal of advancing precision medicine.

American Heart Association.
Circulation: Genomic and Precision Medicine

In the absence of a single large resource to study the determinants of coronary heart disease prognosis , we have established The **GEN**et**I**cs of S**U**b**S**equent **CHD** (GENIUS-CHD) consortium.[19] Assembling studies from across the globe that have recruited patients with different types of CHD at baseline, have acquired prospective follow up, and have stored biological specimens, or genetic data, the consortium aims to: (1) investigate genetic and non-genetic determinants of risk for subsequent CHD, systematically and at scale; and (2) facilitate access to data and expertise, as a platform to foster collaboration among investigators working in the field.

Here we describe the design of the consortium, including details of participating studies, available data and samples, as well as the governance procedures and the consortium's approach to data sharing and collaboration to further advance the stated scientific aims. In addition, we present some early findings from an investigation of the association of patient characteristics and

certain routinely recorded measures on the risk of subsequent events among patients with different types of CHD at baseline.

Methods

In accordance with Transparency and Openness Promotion (TOP) Guidelines, the data, analytic methods, and study materials will be made available to other researchers for purposes of reproducing the results or replicating the procedures. Participating studies received local institutional review board approval and included patients who had provided informed consent at the time of enrolment. The central analysis sites also received waivers from their local institutional review board for collating and analysing summary level data from these individual studies. Full details on the eligibility criteria, definitions of terminology, management of the consortium, and planned projects are provided in Supplementary Materials.

American Heart Association.
Circulation: Genomic and Precision Medicine

Results

The design and structure of the GENIUS-CHD consortium is presented in Figure 1. Studies meeting the main eligibility criteria were identified and invited to participate (Supplementary Methods). In brief, studies are eligible to join the GENIUS-CHD consortium if they meet three inclusion criteria: (1) included individuals with established CHD (defined as the presence of or confirmed history of acute coronary syndrome at baseline, or of coronary artery disease as evidenced by any revascularization procedure (percutaneous coronary intervention or bypass surgery) or demonstrable plaque in any epicardial vessel on direct coronary imaging); (2) acquired prospective follow-up of participants with ascertainment of one or more subsequent cardiovascular disease events as well as all-cause mortality; and, (3) had stored blood samples,

which are viable and suitable for DNA and/or biomarker analysis or previously collected such data prior to sample depletion.

At the time of writing, 57 studies from 18 countries are participating in the consortium and are listed in Table 1. Please refer to www.genius-chd.org for an updated list. Brief narrative descriptions of each study are provided in Supplementary Methods.

The majority of studies are either investigator-led clinical cohorts (n=42), but clinical trials (n=10) and nested case cohort (inception-study design) studies (n=5) are also included. Of the total, 23 studies have included participants at the time of an ACS, while the remainder recruited those with stable CHD or a mixture of the two (e.g. from cardiac catheterization labs). Collectively, 185,614 participants have been enrolled with CHD at baseline (including 812,803 person years of follow-up); of which 170,343 are of self-reported European descent. Recruitment times varied between studies, ranging from the earliest recruitment in 1985 to studies that remain actively recruiting to the present day. All studies enrolled patients >18 years of age, although one study exclusively recruited only those with premature CHD (MI<45 years), while another recruited only older subjects (>70 years). The overall mean age within each study reflects this heterogeneity, ranging from 40-75 years of age, and proportion of male sex ranging from 44-91% (Table 1).

American Heart
Circulation: Genomic and Precision Medicine

Available data:

Core phenotypes: All studies collected data on age, sex and ethnicity. Risk factor data are available for diabetes, obesity and smoking status in almost all participating studies (96%), while data on concentrations of routine blood lipids (total cholesterol, LDL-C, HDL-C and triglycerides) (84%) and blood pressure values at enrolment (82%) were collected by the

majority of studies. Data on statin use at baseline are available in 90% of all participating studies (Table 2).

***Additional phenotypes*:** A list of selected additional phenotypes available by study is presented in Supplementary Table 1. Of note, 79% have available data on plasma C-Reactive Protein (CRP), while coronary disease burden information, from invasive angiography is available in 52% of studies. Finally, over a third of studies have also collected data on physical activity (38%) and socioeconomic status (37%).

Samples: Stored samples are available in most studies for future assay testing and stored frozen. The majority have stored plasma (75%), while others also have serum, blood EDTA, RNA and urine (Supplementary Table 2).

American Heart Association. Circulation: Genomic and Precision Medicine

***DNA & Genotyping*:** More than two thirds of the studies have DNA still available, either pre-extracted or as whole blood collected in EDTA and stored for future genotyping. All studies within the consortium have performed genotyping in some capacity, with genome-wide data available in a subset of studies (Supplementary Table 3).

***Subsequent Events & Follow up*:** The most commonly collected endpoint was all-cause death, collected by all but two studies. CHD death during follow up was collected in 70% of studies, while incident MI was reported by 82% of studies. Studies ascertained endpoints through different means, including telephone contact, in-person patient interviews, clinical chart reviews and linkage to national mortality registers and hospital records (Supplementary Table 4).

<u>Power Calculations</u>

Empirical power was estimated based on a conservative sample size of 150,000 subjects with an event rate of 10% (across the entire follow-up period with a mean of about five years); Figure 2. Given that the GENIUS-CHD consortium is designed to answer multiple questions, power was

estimated for a range of genetic (SNPs) and non-genetic (biomarkers, clinical risk factors) effects.

Minor allele frequencies (MAF) of 0.01, 0.05, 0.10 and 0.25 were examined, representing rare to common SNPs. For each MAF, power was calculated for a range of plausible SNP effects on biomarkers (mean difference (μ) 0.01, 0.03, 0.05) and clinical endpoints (odds ratios (ORs) of 1.02, 1.05, 1.10). For the association of SNPs with biomarkers, power was 80% (alpha=0.05) or more unless the SNP was rare (MAF of 0.01) or the effect size was small (e.g., 0.01 per allele). For the association of SNPs with clinical endpoints, power was close to 80% when the effect size was large (OR $\geq$ 1.10) or the MAF was $\geq$ 0.10.

Power of observational (i.e. non-genetic) analysis was >99% for both continuous and binary exposures unless the OR was close to 1. In addition to continuous and binary outcome data, GENIUS-CHD also collects time-to-event data. Given the similarity (in most empirical settings) between OR and hazard ratios,[20] similar power is to be expected for time-to-event analysis.

American Heart Association.
Circulation: Genomic and Precision Medicine

Initial Analysis:

To examine the feasibility of the federated analysis approach, we sought to collect data on participant characteristics, cardiovascular and mortality outcomes and association analyses with common clinical exposures. A standardised dataset was developed, with a federated analysis conducted using standardised statistical scripts. The summary level outputs generated were then shared with the coordinating centres for aggregating and meta-analysis (Supplementary Methods).

Participant characteristics

Detailed characteristics of participants by study are presented in Table 2. Prevalence of risk factors varied by study, with diabetes ranging from 4 to 76%; smoking from 8 to 79%. Mean total cholesterol by study ranged from 166.3 to 239.8 mg/dL, mean BMI ranged from 24.8 to 30.2kg/m^2 and mean systolic blood pressure from 117 to 153mmHg. The proportion of participants with prior revascularization or MI was high in most studies reflecting the inclusion criteria for the consortium (Table 2).

Review of returned outputs from the federated analysis revealed good quality data with estimates falling within expected ranges for age, sex, and other variables such as body mass index (Supplementary Figure 1).

Endpoints

American Heart Association.
Circulation: Genomic and Precision Medicine

The primary endpoint pre-selected for the study was a composite of coronary death or MI (CHD death/MI). Mean follow up was estimated in each study and ranged between 9 months and 15 years. In total we estimated over 748,000 person years of follow up were available for the primary endpoint analysis.

Information was collected on 10 subsequent event endpoints in the initial feasibility analysis. Across all studies, the most frequently occurring event during prospective follow up was the composite of all cardiovascular events (27%); followed by revascularization (21.8%); all-cause mortality (15%); coronary death or MI (14.2%); myocardial infarction (10.7%); cardiovascular death (8.3%); coronary death (8%); heart failure (6.3%); all stroke (3.6%) and ischaemic stroke (3.4%).

Association analyses:

As a feasibility analysis, we examined associations between age, male sex and smoking with the primary endpoint CHD death/MI as well as with the nine other secondary endpoints, to investigate any differential associations across discrete subsequent events.

In analyses unrestricted by race or type of CHD at baseline, but adjusted for sex, there was a strong association between each 5-year increment in age with subsequent risk of the primary endpoint of CHD death/MI (HR 1.15, 95% CI 1.14-1.16). The largest observed hazard ratios were for all-cause mortality (HR 1.36, 95% CI 1.35-1.37), cardiovascular death (HR 1.36, 95% C.I. 1.35, 1.38) and heart failure (HR 1.25, 95% CI 1.24, 1.27), while a smaller risk increase was observed for MI (HR 1.06, 95% CI 1.05-1.07). The risk of future revascularization, however, showed a modest inverse association with increasing age (HR 0.98, 95% CI 0.98-0.99) (Figure 3).

American Heart Association
Circulation: Genomic and Precision Medicine

Male sex was a risk factor for CHD death/MI (HR 1.17, 95% CI 1.13- 1.21) and other coronary and mortality endpoints (Figure 4) after adjustment for age. In particular, the largest observed hazard ratio was for risk of revascularization, which was considerably higher in males (HR 1.24, 95% C.I. 1.20, 1.27). In contrast, there was no strong evidence for an association between male sex and risk of stroke (ischemic or any stroke, Figure 4).

Finally, in analyses adjusted for age and sex, current smoking (compared to prior or never smoking) at the time of enrolment showed a strong association with risk of future CHD death/MI (HR 1.43, 95% C.I. 1.35-1.51). Similarly, smoking was associated with an increased risk of all-cause mortality (HR 1.53, 95%CI 1.47-1.58) and an increased risk of all other end points, although there was no strong evidence for an association with incident revascularization (HR 1.02, 95% CI 0.99-1.05, Figure 5).

When stratified by type of CHD at enrolment, i.e. among those presenting with an acute event, those with stable CAD without ever having had an MI and those with stable CAD and a prior MI, the findings were similar and directionally concordant to non-stratified analyses described above, for all endpoints (data not shown).

Discussion

The GENIUS-CHD Consortium is a global collaborative effort engaging 57 studies, including almost 185,000 patients with established CHD, for whom genetic and prospective follow-up data are available. It brings together over 170 domain experts, including clinicians, data scientists, geneticists and epidemiologists, all engaged in improving our understanding of the determinants of subsequent event risk in these patients. With an agreed governance structure and a proven federated analysis approach, we anticipate that this consortium will be a valuable long-term resource for genetic and non-genetic research in this field.

American Heart Association. Circulation: Genomic and Precision Medicine

Genetic association studies for CHD disease progression, recurrence and adverse events after a CHD event may have particular utility for identifying novel causal pathways and therapeutic targets that may be different than those for first events, a concept recently supported by research in other disease areas.[21] However, information on the determinants of subsequent CHD event risk is scarce, in contrast to the extensive knowledge about risk factors for a first CHD event. This disparity is due in part to the relatively small sample sizes of individual studies in the secondary prevention setting. While larger registry and electronic health care records (EHR) efforts will result in higher numbers, they typically suffer from the lack of necessary depth of phenotyping, accuracy, and availability of bio-specimens to infer further biological insights.[22, 23] In contrast, large population studies with detailed phenotyping have relatively small

numbers of mostly stable CHD patients, who have survived many years after their index event.[24, 25] By bringing together multiple investigator-led studies, the GENIUS-CHD consortium aims to address and overcome this major limitation to subsequent CHD risk research.

Importantly, the scale and depth of the GENIUS-CHD consortium offers greater scope to tackle key challenges within subsequent CHD risk-related research. Firstly, CHD is a heterogeneous phenotype, consisting of stable, unstable and pathologically distinct subtypes, which have often been combined for individual studies to satisfy the need for statistical power. With the sample size available in GENIUS-CHD, we anticipate being able to disaggregate CHD into more precise sub-phenotypes such as acute vs. stable CHD at baseline, or those with vs. without prior MI, which may help uncover relevant biological differences.[26] Additional stratification on variables such as sex, time period of recruitment, duration of follow-up, country of study, LV function and treatment (such as statin, blood pressure lowering and anti-platelet agent use) will also be possible, providing greater insights into the modifying influences of these variables on outcome.

American Heart Association
Circulation: Genomic and Precision Medicine

A major strength of the consortium is the use of a federated analysis approach that permits individual level analysis without the need for sharing either samples or the individual datasets themselves, thereby overcoming major privacy and governance hurdles. The effort has been successful because (1) participation is entirely voluntary, with studies only participating in those analyses they feel are of value, or to which they have the capacity to contribute; (2) ownership of all data and samples remain with the PI and are not shared nor stored centrally; and (3) there are open and transparent governance procedures. Our feasibility analysis has demonstrated that this federated approach works well and yields results that are consistent and suitable for high quality meta-analysis.

Indeed, supported by this initial feasibility analysis, our findings demonstrate the validity of the data collected by confirming the anticipated associations of increasing age, male sex, and current smoking with higher risks of subsequent CHD death/MI during follow up. Furthermore, by exploring multiple individual and composite endpoints, we can begin to unravel associations not discoverable in smaller studies. For example, we find that the risk of incident revascularization is lower with advancing age but higher for male sex and neutral for smoking. Plausible explanations may exist for each of these findings (e.g. an association induced by clinical practice, with fewer older people being offered invasive treatments), but importantly they highlight the value of exploring multiple endpoints at appropriate scale. This is especially relevant when exploring novel biomarkers or drug targets as these may, in turn be used to inform clinical testing strategies and choice of endpoints to study in trials.

American Heart Association. Circulation: Genomic and Precision Medicine

By virtue of the expertise it has assembled, the consortium is also well placed to address important methodological issues surrounding prognosis research in general. For example, selection bias is a key concern, whereby it is conceivable that those at highest risk may die early and not enter any of the member studies for evaluation (survival bias), or selection on an indexing event itself may distort patient characteristics and impact association findings (index event bias).[27] In addition, treatment effects may alter the trajectory of disease by stabilizing or regressing plaque burden or altering baseline risk, such as with high dose statin or PCSK9 inhibitor use.[17, 28] To address these and other issues, the consortium has established working groups of relevant national and international experts to explore the extent and impact of such biases/effects and if needed, to develop approaches to address these.[29]

There are inherent challenges to overcome when working with diverse multiple studies, including variations in definitions and processes for data collection and curation across different

studies in different centres and different countries. The consortium members have attempted to standardize common data elements, for example the measurement units for quantitative traits. Variability between studies will persist, but we anticipate that the overall size of the effort will help reduce the impact of such study level heterogeneity on any findings, which will also be explored through subgroup analyses where possible (e.g. country, study size, year of first recruitment). Analytical challenges will additionally include dealing with variability in length of follow up across studies, handling multiple subsequent events along with competing risks, as well as confounding by treatment and selection biases as described above. The collective experience of the consortium members will be leveraged to address these as carefully as possible within each future analysis. Finally, factors influencing enrolment into genetic studies of CHD may limit the generalizability of findings. Men are over represented in participating CHD studies, partly reflecting sex-differential prevalence of disease but also underpinning a wider concern about under-investigation of women, who may be inadvertently excluded given that entry criteria for most studies relies on documented presence of CHD. Similarly, many studies in the consortium have recruited mostly Europeans, limiting the opportunity to explore hypotheses in other ethnic groups. The steering committee is conscious of these imbalances and is actively seeking studies enriched for women and non-European participants to join the collaboration. In summary, the GENIUS-CHD consortium is a global collaboration among investigators who have recruited patients with CHD into multiple individual studies, seeking to gain a better understanding of subsequent CHD event risk and enhance secondary prevention. It seeks to be an open, collegiate and transparent effort and we invite investigators with suitable studies to join and collectively enhance research efforts in this domain.

American Heart Association
Circulation: Genomic and Precision Medicine

Acknowledgments: The GENIUS CHD collaborators would like to express their immense gratitude to all patients who participated in each of the individual studies as well as the many personnel who helped with recruitment, collection, curation, management and processing of the samples and data.

Sources of Funding: The funder(s) of the study had no role in study design, data collection, data analysis, data interpretation, or writing of the report. Within GENIUS-CHD, all participating investigators and sponsors who contributed data and analyses are acknowledged irrespective of academic or industry affiliations.

Specific funding statements: Dr Patel is funded by a British Heart Foundation Intermediate Fellowship (FS/14/76/30933). This research was also supported by the National Institute for Health Research University College London Hospitals Biomedical Research Centre; Dr Schmidt is funded by BHF grant PG/18/5033837 ; Dr Holmes works in a unit that receives funding from the UK Medical Research Council and is supported by a British Heart Foundation Intermediate Clinical Research Fellowship (FS/18/23/33512) and the National Institute for Health Research Oxford Biomedical Research Centre.; The AGNES study was supported by research grants from the Netherlands Heart Foundation (2001D019, 2003T302, 2007B202 and the PREDICT project (CVON 2012-10)), the Leducq Foundation (grant 05-CVD) and the Center for Translational Molecular Medicine (CTMM COHFAR). ; The Cleveland Clinic Genebank Study was supported in part by NIH grants R0133169, R01ES021801, R01MD010358, and R01ES025786, R01HL103866, R01DK106000, R01HL126827, P20HL113452., P01HL098055, P01HL076491, and R01HL103931; The Clinical Cohorts in Coronary disease Collaboration (4C) study was supported in part by NIHR and Barts Charity; The Corogene study was supported by grants from Aarno Koskelo Foundation, Helsinki University Central Hospi-tal special government funds (EVO #TYH7215, #TKK2012005, #TYH2012209, #TYH2014312), and Finnish Foundation for Cardiovascular research ; CABGenomics was supported by Stanton Shernan, C. David Collard, Amanda A. Fox/R01 HL 098601 NHLBI; The CDCS and PMI studies were funded by the Health Research Council and Heart Foundation of New Zealand; Dr Samman-Tahnan is supported by the National Institutes of Health/ National Institutes of Aging grant AG051633; Dr Sandesara is supported by the Abraham J. & Phyllis Katz Foundation (Atlanta, GA, USA); The Emory Cardiovascular Biobank is supported by National Institutes of Health (NIH) grants

American Heart Association. Circulation: Genomic and Precision Medicine

5P01HL101398-02, 1P20HL113451-01, 1R56HL126558-01, 1RF1AG051633-01, R01 NS064162-01, R01 HL89650-01, HL095479-01, 1U10HL110302-01, 1DP3DK094346-01, 2P01HL086773-06A1; The Estonian Biobank was funded by EU H2020 grant 692145, Estonian Research Council Grant IUT20-60, IUT24-6, PUT1660, PUT735 and European Union through the European Regional Development Fund Project No.2014-2020.4.01.15-0012 GENTRANSMED, NIH – GIANT, ERA-CVD grant Detectin-HF and 2R01DK075787-06A1.; FAST-MI (French Registry of Acute ST-Elevation or non- ST-elevation Myocardial Infarction) 2005 is a registry of the French Society of Cardiology, supported by unrestricted grants from Pfizer and Servier. Additional support was obtained from a research grant from the French Caisse Nationale d'Assurance Maladie. ; GENESIS-PRAXY is funded by the Canadian Institutes of Health Research and Heart and Stroke Foundations of Alberta, NWT & Nunavut, British Columbia and Yukon, Nova Scotia, Ontario, and Quebec (HSFC); The GENDEMIP study was supported by Project (MH, Czech Republic) No. 00023001 (ICEM, Prague); GoDARTS was funded by the Wellcome Trust (072960/Z/03/Z, 084726/Z/08/Z, 084727/Z/08/Z, 085475/Z/08/Z, 085475/B/08/Z) and as part of the EU IMI-SUMMIT programme. C.N.A.P. has received grant funding from the Wellcome Trust to develop the GoDARTS cohort. ; Dr Mordi is supported by an NHS Education of Scotland/Chief Scientist Office Postdoctoral Clinical Lectureship (PCL 17/07); the GENECOR stuidy was supported in part by the Italian Ministry of Research's Fund for Basic Research (FIRB 2005); GRACE UK was supported in part by an Educational Grant from Sanofi Aventis; Award from Chief Scientist Office, Scotland; INVEST-GENES was supported by the National Institute of Health Pharmacogenomics Research Network grant U01-GM074492, NIH R01 HL074730, University of Florida Opportunity Fund, BASF Pharma and Abbott Laboratories. ; IATVB was supported by Epidemiologia e Genetica della Morte Improvvisa in Sardegna; The KAROLA study has received financial support by the German Ministry of Education and Research (01GD9820/0 and 01ER0814), by the Willy-Robert-Pitzer Foundation, and by the Waldburg-Zeil Clinics Isny.; The KRAKOW GENIUS Study was supported by a grant from the Polish Ministry of Science and Higher Education, no. NN402083939 and the National Science Centre, no. 2013/09/B/NZ5/00770; LIFE-Heart was funded by the Leipzig Research Center for Civilization Diseases (LIFE). LIFE is an organizational unit affiliated to the Medical Faculty of the University of Leipzig. LIFE is funded by means of the European Union, by the European Regional Development Fund (ERDF) and by

American Heart Association
Circulation: Genomic and Precision Medicine

funds of the Free State of Saxony within the framework of the excellence initiative.; The LURIC study was supported by the 7th Framework Program (AtheroRemo, grant agreement number 201668 and RiskyCAD, grant agreement number 305739) of the European Union; Dr JG Smith was supported by grants from the European Research Council, Swedish Heart-Lung Foundation, the Swedish Research Council, the Crafoord Foundation, governmental funding of clinical research within the Swedish National Health Service, Skåne University Hospital in Lund, and the Scania county, a generous donation from the Knut and Alice Wallenberg foundation to the Wallenberg Center for Molecular Medicine at Lund University, and funding from the Swedish Research Council and Swedish Foundation for Strategic Research to the Lund University Diabetes Center.; The NEAPOLIS CAMPANIA study was suppported by European Research Council Advanced Grant (CardioEpigen, #294609);Italian Ministry of Health (PE-2013-02356818);Italian Ministry of Education, University and Research (2015583WMX) ; The North East Poland Myocardial Infarction Study was supported by grant N N 402 529139 from the National Science Center (Poland); Dr Vilmundarson is supported by a graduate fellowship of the University of Ottawa Heart Institute; OHGS was funded in part by a Heart and Stroke Foundation grant; Dr Stott was supported in part by an investgator initiated grant from Bristol Myers Squibb USA; The PROSPER study was supported by an investigator initiated grant obtained from Bristol-Myers Squibb. Prof. Dr. J. W. Jukema is an Established Clinical Investigator of the Netherlands Heart Foundation (grant 2001 D 032). Support for genotyping was provided by the seventh framework program of the European commission (grant 223004) and by the Netherlands Genomics Initiative (Netherlands Consortium for Healthy Aging grant 050-060-810). ; The RISCA study ws supported in part by FRSQ, HSFC, Merck Frost Canada, Pfizer Canada; The SHEEP study was supported by grants from the Swedish Council for Work Life and Social Research, and the Stockholm County Council. ; The TNT trial was sponsored by Pfizer who granted access to data, Genotyping of the samples was funded in part by grants from Genome Canada and Genome Quebec and the Canadian Institutes of Health Research (CIHR).; Dr Arsenault holds a junior scholar award from the Fonds de recherche du Quebec- Sante (FRQS); Dr. Cresci is supported, in part, by the National Institutes of Health (Cresci R01 NR013396). The TRIUMPH study was sponsored by the National Institutes of Health: Washington University School of Medicine SCCOR Grant P50 HL077113. ; The UCP studies were funded by the Netherlands Heart Foundation and the Dutch Top Institute Pharma

American Heart Association. Circulation: Genomic and Precision Medicine

Mondriaan Project; The Verona Heart Study was supported by the Cariverona Foundation; Veneto Region; Italian Ministry of Education, University, and Research (MIUR); LURM (Laboratorio Universitario di Ricerca Medica) Research Center, University of Verona; The Warsaw ACS Registry is supported by grant N R13 0001 06 from The National Centre for Research and Development (NCBiR), Statutory Grant from Medical University of Warsaw; Dr Nelson is funded by the British Heart Foundation; Prof. Samani is funded by the British Heart Foundation and is a NIHR Senior Investigator; Prof Hingorani is a NIHR Senior Investigator. Prof Asselbergs is supported by UCL Hospitals NIHR Biomedical Research Centre, EU/EFPIA Innovative Medicines Initiative 2 Joint Undertaking BigData@Heart grant n° 116074, the European Union’s Horizon 2020 research and innovation programme under the ERA-NET Co-fund action N°01KL1802 (Druggable-MI-gene) jointly funded by the Dutch Heart Foundation and Netherlands Organization for Health Research and Development (ZonMw).

American Heart Association. Circulation: Genomic and Precision Medicine

Disclosures: Dr Patel has received speaker fees and honoraria from Amgen, Sanofi and Bayer and research funding from Regeneron; Dr Holmes has collaborated with Boehringer Ingelheim in research, and in accordance with the policy of the The Clinical Trial Service Unit and Epidemiological Studies Unit (University of Oxford), did not accept any personal payment; Dr Akerblom has received institutional research grant and speakers fee from AstraZeneca, institutional research grant from Roche Diagnostics.; Dr James has received grants from AstraZeneca, The Medicines Company, Swedish heart and lung foundation, Swedish research council, Janssen; personal fees from Bayer; Dr Hagstrom declares being an expert committee member, lecture fees, and institutional research grant from Sanofi, and Amgen; institutional research grants from AstraZeneca, and GlaxoSmithKline; expert committee member and lecture fees NovoNordisk and Behringer.; Dr Held declares institutional research grant, advisory board member and speaker’s bureau from AstraZeneca; institutional research grants from Bristol-Myers Squibb Merck & Co, GlaxoSmithKline, Roche Diagnostics. Advisory board for Bayer and Boehringer Ingelheim; Dr Lindholm has received institutional research grants from AstraZeneca, and GlaxoSmithKline; Speaker fees from AstraZeneca, Speaker fees from AstraZeneca.; Dr Siegbahn has received institutional research grants from AstraZeneca, Boehringer Ingelheim, Bristol-Myers Squibb/Pfizer, Roche Diagnostics, GlaxoSmithKline.; Dr. J.M. ten Berg reports receiving fees for board membership from AstraZeneca, consulting fees

from AstraZeneca, Eli Lilly, and Merck, and lecture fees from Daiichi Sankyo and Eli Lilly, AstraZeneca, Sanofi and Accumetrics; Prof Wallentin reports institutional research grants, consultancy fees, lecture fees, and travel support from Bristol-Myers Squibb/Pfizer, AstraZeneca, GlaxoSmithKline, Boehringer Ingelheim; institutional research grants from Merck & Co, Roche Diagnostics; consultancy fees from Abbott; and holds a patent EP2047275B1 licensed to Roche Diagnostics, and a patent US8951742B2 licensed to Roche Diagnostics. Prof Asselbergs has received research funding from Regeneron, Pfizer, Sanofi.

References:

1. Ford ES, et al. Proportion of the decline in cardiovascular mortality disease due to prevention versus treatment: public health versus clinical care. *Annu Rev Public Health*. 2011;32:5-22.

2. Sidney S, et al. Recent Trends in Cardiovascular Mortality in the United States and Public Health Goals. *JAMA Cardiol*. 2016;1:594-9.

3. Ford ES, et al. Explaining the decrease in U.S. deaths from coronary disease, 1980-2000. *N Engl J Med*. 2007;356:2388-98.

4. Benjamin EJ, et al. Heart Disease and Stroke Statistics-2017 Update: A Report From the American Heart Association. *Circulation*. 2017;135:e146-e603.

5. Scarborough PB, P; Wickramasinghe, K; Smolina, K; Mitchell, C; Rayner, M. *Coronary heart disease statistics 2010 edition*. London: British Heart Foundation; 2010.

6. Yusuf S, et al. Effect of potentially modifiable risk factors associated with myocardial infarction in 52 countries (the INTERHEART study): case-control study. *Lancet*. 2004;364:937-52.

7. Deloukas P, et al. Large-scale association analysis identifies new risk loci for coronary artery disease. *Nat Genet*. 2013;45:25-33.

8. Fox KA, et al. Prediction of risk of death and myocardial infarction in the six months after presentation with acute coronary syndrome: prospective multinational observational study (GRACE). *BMJ*. 2006;333:1091.

9. Schiele F, et al. Coronary artery disease: Risk stratification and patient selection for more aggressive secondary prevention. *Eur J Prevent Cardiol*. 2017;24:88-100.

10. Schwartz GG, et al. Effects of dalcetrapib in patients with a recent acute coronary syndrome. *N Engl J Med*. 2012;367:2089-99.

American Heart Association. Circulation: Genomic and Precision Medicine

11. White HD, et al. Darapladib for preventing ischemic events in stable coronary heart disease. *N Engl J Med*. 2014;370:1702-11.

12. Boden WE, et al. Niacin in patients with low HDL cholesterol levels receiving intensive statin therapy. *N Engl J Med*. 2011;365:2255-67.

13. Armitage JM, et al. Effects of homocysteine-lowering with folic acid plus vitamin B12 vs placebo on mortality and major morbidity in myocardial infarction survivors: a randomized trial. *JAMA*. 2010;303:2486-94.

14. Global BMIMC, et al. Body-mass index and all-cause mortality: individual-participant-data meta-analysis of 239 prospective studies in four continents. *Lancet*. 2016;388:776-86.

15. Wang ZJ, et al. Association of body mass index with mortality and cardiovascular events for patients with coronary artery disease: a systematic review and meta-analysis. *Heart*. 2015;101:1631-8.

16. Cannon CP, et al. Ezetimibe Added to Statin Therapy after Acute Coronary Syndromes. *N Engl J Med*. 2015;372:2387-97.

17. Sabatine MS, et al. Evolocumab and Clinical Outcomes in Patients with Cardiovascular Disease. *N Engl J Med*. 2017;376:1713-1722.

18. Ridker PM, et al. Antiinflammatory Therapy with Canakinumab for Atherosclerotic Disease. *N Engl J Med*. 2017;377:1119-1131.

19. Patel RS, et al. The GENIUS-CHD consortium. *Eur Heart J*. 2015;36:2674-6.

20. Rothman KJ, et al. *Modern epidemiology*; 2008.

21. Lee JC, et al. Genome-wide association study identifies distinct genetic contributions to prognosis and susceptibility in Crohn's disease. *Nat Genet*. 2017;49:262-268.

22. Ohman EM, et al. The REduction of Atherothrombosis for Continued Health (REACH) Registry: an international, prospective, observational investigation in subjects at risk for atherothrombotic events-study design. *Am Heart J*. 2006;151:786 e1-10.

23. Psaty BM, et al. The Cohorts for Heart and Aging Research in Genomic Epidemiology (CHARGE) Consortium as a model of collaborative science. *Epidemiology*. 2013;24:346-8.

24. Collins R. What makes UK Biobank special? *Lancet*. 2012;379:1173-4.

25. Chen Z, et al. China Kadoorie Biobank of 0.5 million people: survey methods, baseline characteristics and long-term follow-up. *Int J Epidemiol*. 2011;40:1652-66.

American Heart Association.
Circulation: Genomic and Precision Medicine

26. Reilly MP, et al. Identification of ADAMTS7 as a novel locus for coronary atherosclerosis and association of ABO with myocardial infarction in the presence of coronary atherosclerosis: two genome-wide association studies. *Lancet*. 2011;377:383-92.

27. Dahabreh IJ, et al. Index event bias as an explanation for the paradoxes of recurrence risk research. *JAMA*. 2011;305:822-3.

28. Nicholls SJ, et al. Effect of Evolocumab on Progression of Coronary Disease in Statin-Treated Patients: The GLAGOV Randomized Clinical Trial. *JAMA*. 2016;316:2373-2384.

29. Hu YJ, et al. Impact of Selection Bias on Estimation of Subsequent Event Risk. *Circulation Cardiovascular Genetics*. 2017;10.

American Heart Association
Circulation: Genomic and Precision Medicine

Table 1: Overview of each study participating in the GENIUS-CHD consortium

Alias	Cohort Name	Country	Study Design	Recruitment Period	CHD Type	Total recruited with CHD	European ancestry (%)	Europeans recruited with CHD	Mean Follow up Time (SD)	Age (SD)	Male (%)	PubMED ID
4C	Clinical Cohorts in Coronary disease Collaboration (4C)	UK	Clinical Cohort	2009-2014	CAD	3345	54.8	1832	2.56 (0.95)	61.8 (12.14)	61.5	NA
AGNES	Arrhythmia Genetics in The Netherlands	Netherlands	Clinical Cohort	2001-2005	ACS	1459	100.0	1459	6.73 (4.75)	57.8 (10.73)	79.2	20622880
ANGES	Angiography and Genes Study	Finland	Clinical Cohort	2002-2005	Mixed	588	100.0	588	8.20 (4.47)	64.1 (9.59)	65.5	21640993
ATVB	Italian Atherosclerosis, Thrombosis and Vascular Biology Group	Italy	Clinical Cohort	1997-2006	ACS	1741	100.0	1741	10.47 (4.45)	40.0 (4.40)	90.8	21757122
CABGenomics	CABG Genomics	USA	Clinical Cohort	2001-2014	Mixed	2694	85.5	2303	6.9 (3.5)	64.4 (10.38)	79	25649697
CARDIOLINES	CardioLines	Netherlands	Clinical Cohort	2011-	Mixed	1269	75.0	1,692	1.3 (0.5)	63.5 (11.6)	72.8	NA
CDCS	Coronary Disease Cohort Study	New Zealand	Clinical Cohort	2002-2009	ACS	2139	91.4	1956	5.21 (2.15)	67.4 (12.01)	71.3	20400779
COGEN	The Copenhagen Cardiovascular Genetic study	Denmark	Clinical Cohort	2011-2017	Mixed	3709	95.0	3,904	5.5 (1.01)	70.1 (17.4)	67.5	In press
COROGENE	Corogene Study	Finland	Clinical Cohort	2006-2008	ACS	1489	100.0	1489	7.7 (0.5)	64.7 (11.88)	70.9	21642350
CTMM	Circulating Cells	The Netherlands	Clinical Cohort	2009-2011	Mixed	713	96.5	688	0.97 (0.37)	62.6 (10.08)	69	23975238
CURE	Cure-Genetics Study	Canada	RCT	1998-2000	ACS	12434	82.1	10203	0.78 (0.28)	65.4 (11.19)	61.4	11102254
EGCUT	Estonian Biobank	Estonia	Population	2002-2011	CAD	2783	100.0	2783	6.65 (2.93)	66.6 (10.99)	51.5	24518929
EMORY	Emory Cardiovascular Biobank	USA	Clinical Cohort	2004-	Mixed	5873	72.0	4229	4.49 (3.15)	65.4 (11.74)	68.7	20729229
ERICO	Estratégia de Registro de Insuficiência Coronariana	Brazil	Clinical Cohort	2009-2014	ACS	738	61.0	450	2.85 (1.48)	63.8 (13.35)	56	23644870
FASTMI2005	The French Registry of Acute ST elevation MI	France	Clinical Cohort	2005-	ACS	3669	100.0	3669	1.72 (0.63)	67.3 (13.94)	68.5	17893635
FINCAVAS	Finnish Cardiovascular Study	Finland	Clinical Cohort	2001-2008	Mixed	1671	100.0	1671	8.57 (3.99)	60.9 (11.04)	69.4	16515696
FRISCII	FRISCII Study	Sweden	RCT	1996-1998	ACS	3147	99.3	3125	7.46 (2.09)	66.3 (9.82)	69.5	10475181
GENDEMIP	GENetic DEtermination of Myocardial Infarction in Prague	Czech Republic	Clinical Cohort	2006-2009	ACS	1302	100.0	1302	1.13 (0.78)	56.5 (8.66)	74.4	23249639
GENEBANK	Cleveland Clinic Genebank Study	USA	Clinical Cohort	2001-2007	Mixed	2345	100.0	2345	3.00 (0.00)	61.5 (11.06)	74.3	21475195
GENESIS-PRAXY	GENdEr and Sex determInantS of cardiovascular disease: From bench to beyond-Premature Acute Coronary Syndrome (GENESIS-PRAXY)	Canada	Clinical Cohort	2009-2013	ACS	784	99.4	779	1.00 (0.00)	48.3 (5.62)	69.1	22607849
GENOCOR	Genetic Mapping for Assessment of Cardiovascular Risk	Italy	Clinical Cohort	2007-2010	Mixed	497	100.0	497	5.68 (1.20)	65.2 (8.47)	86.7	22717531
GEVAMI	The GEnetic causes to Ventricular Arrhythmia in patients during first ST-elevation Myocardial Infraction	Denmark	Clinical Cohort	2011-	ACS	1033	100.0	1033	3.93 (1.40)	59.5 (10.37)	79.3	25559012
GoDARTS incident	Genetics of Diabetes Audit and Research in Tayside Scotland (I)	Scotland	Population	2004 - 2012	CAD	1261	99.8	1258	3.47 (2.95)	71.3 (10.91)	61.1	29025058
GoDARTS prevalent	Genetics of Diabetes Audit and Research in Tayside Scotland (P)	Scotland	Population	2004 - 2012	CAD	2514	99.7	2507	6.48 (3.06)	69.1 (9.41)	65.9	29025058
GRACE_B	Global Registry of Acute Coronary Events - Belgium	Belgium	Clinical Cohort	1999-2010	ACS	734	100.0	734	4.25 (1.80)	65.9 (11.91)	75.8	20231156
GRACE_UK	Global Registry of Acute Coronary Events - UK	UK	Clinical Cohort	2001-2010	ACS	1443	100.0	1443	9.54 (2.68)	64.3 (12.21)	69.6	20231156
IDEAL	Incremental Decrease in End Points Through Aggressive lipid Lowering (IDEAL)	Canada	RCT	1999-2005	ACS	8888	99.3	8823	4.63 (0.82)	61.8 (9.47)	80.8	16287954
INTERMOUNTAIN	Intermountain Heart Collaborative Study	USA	Clinical Cohort	1993-2009	Mixed	7556	89.5	6763	8.56 (5.39)	61.2 (11.06)	66.7	20691829
INVEST	INternational VErapamil SR Trandolopril STudy GENEtic Substudy INVEST-GENES	USA/ International	RCT	1997-2003	CAD	5979	38.0	2270	2.83 (0.82)	66.1 (9.70)	44	21372283, 17700361
JUMC	Krakow-GENIUS-CHD	Poland	Clinical Cohort	2010-2014	Mixed	747	100.0	747	0.84 (0.34)	68.3 (10.26)	71.6	28444280, 27481134
KAROLA	Karola Study	Germany	Clinical Cohort	1999-2000	Mixed	1206	100.0	1206	11.62 (3.01)	58.7 (8.15)	84.2	24829374
LIFE-Heart	Leipzig (LIFE) Heart Study	Germany	Clinical Cohort	2006-2014	Mixed	5564	100.0	5564	1.62 (2.03)	63.9 (11.09)	77.2	22216169
LURIC	The LUdwigshafen RIsk and Cardiovascular Health Study	Germany	Clinical Cohort	1997-2000	Mixed	2320	100.00	2320	8.58 (3.18)	63.8 (9.92)	76.6	11258203
MDCS	Malmo Diet and Cancer Study	Sweden	Population	1991-1996	CAD	4,546	100.00	4,546	8.3 (8.0)	58.0 (7.6)	60.2	19936945
NE_POLAND	North East Poland Myocardial Infarction Study	Poland	Clinical Cohort	2001-2005	ACS	646	100.0	646	7.20 (2.75)	62.3 (11.84)	75.4	26086777
NEAPOLIS	Neapolis Campania Italia	Italy	Clinical Cohort	2008-2012	Mixed	1394	100.0	1394	1.07 (0.54)	67.6 (10.50)	74.5	24262617
OHGS	Ottawa Heart Genomics Study	Canada	Clinical Cohort	2010-2013	Mixed	546	100.0	546	1.77 (0.27)	65.6 (11.11)	73.8	NA
PERGENE	Perindopril Genetic Association Study (EUROPA)	Netherlands	RCT	1997-2000	CAD	8746	99.0	8656	4.20 (0.62)	59.9 (9.27)	85.6	19082699

Circulation: Genomic and Precision Medicine

Alias	Cohort Name	Country	Study Design	Recruitment Period	CHD Type	Total recruited with CHD	European ancestry (%)	Europeans recruited with CHD	Mean Follow up Time (SD)	Age (SD)	Male (%)	PubMED ID
PLATO	The Study of Platelet Inhibition and Patient Outcomes	International	RCT	2006-2008	ACS	18624	98.3	18315	0.86 (0.24)	62.6 (10.96)	69.5	19332184
PMI	Post Myocardial Infarction Study	New Zealand	Clinical Cohort	1994-2001	ACS	1057	91.1	963	8.56 (3.58)	62.8 (10.56)	78	12771003
POPular	The POPular study	Netherlands	Clinical Cohort	2005-2007	Mixed	1024	98.2	1006	1.00 (0)	63.8 (10.39)	74.6	20179285
POPular Genetics	The POPular GENETICS Study	Netherlands & Belgium	RCT	2011-2017	ACS	2481	94.3	2287	1.00 (0)	NA	74.9	24952855
PROSPER	Prospective Study of Pravastatin in the Elderly at Risk	Netherlands	RCT	1997-1999	CAD	893	100.0	893	3.15 (0.71)	75.4 (3.38)	70.3	10569329
RISCA	Recurrance and Inflammation in the Acute Coronary Syndromes Study	Canada	Clinical Cohort	2001-2002	ACS	1054	100.0	1054	1.22 (0.18)	61.8 (11.45)	75.9	18549920
SHEEP	Stockholm Heart Epidemiology Program (SHEEP)	Sweden	Clinical Cohort	1992-1995	ACS	1150	100.0	1150	14.87 (5.91)	59.3 (7.21)	70.7	17667644
SMART	Second Manifestations of Arterial Disease	Netherlands	Clinical Cohort	1999-2010	Mixed	3057	98.2	3001	6.77 (3.86)	60.5 (9.31)	81.7	10468526
STABILITY	Stabilization of Atherosclerotic Plaque by Initiation of Darapladib Therapy trial	International	RCT	2008-2010	CAD	10786	86.1	9287	3.60 (0.57)	64.7 (9.10)	82	24678955
THI	Texgen	USA	Clinical Cohort	2001-2008	ACS	3875	73.1	2834	5.50 (3.42)	63.6 (10.61)	74.9	21414601
TNT	Treating to New Targets	Canada	RCT	1998-1999	CAD	10000	94.1	9409	4.36 (1.47)	61.1 (8.82)	81.6	15755765
TRIUMPH	Translational Research Investigating Underlying Disparities in Acute Myocardial Infarction Patient's Health Status	USA	Clinical Cohort	2005-2008	ACS	2062	100.0	2062	0.97 (0.15)	59.8 (12.10)	72.2	21772003
UCORBIO	Utrecht Coronary Biobank	Netherlands	Clinical Cohort	2011-2014	Mixed	1493	72.4	1081	1.6 (0.9)	65.4 (10.27)	75.6	NA
UCP	Utrecht Cardiovascular Pharacogenetics Study	Netherlands	Clinical Cohort	1985-2010	Mixed	1508	100.0	1508	8.00 (4.16)	64.1 (9.97)	75.4	25652526
UKB	UK Biobank	UK	Population	2006-2010	CAD	12045	94.2	11342	6.39 (1.72)	69.9 (6.07)	80.6	1001779
VHS	Verona Heart Study	Italy	Clinical Cohort	1996-	CAD	939	100.0	939	5.62 (2.97)	61.3 (9.74)	81	10984565
VIVIT	Vorarlberg Institute for Vascular Investigation and Treatment (VIVIT) Study	Austria	Clinical Cohort	1999-2008	CAD	1447	99.8	1444	7.43 (2.91)	64.5 (10.45)	72	24265174
WARSAW ACS	Warsaw ACS Genetic Registry	Poland	Clinical Cohort	2008-2011	ACS	681	100.0	681	2.97 (1.16)	63.5 (11.84)	74.2	NA
WTCC	WTCCC CAD Study	UK	Clinical Cohort	1998-2003	Mixed	1926	100.0	1926	10.05 (2.81)	60.0 (8.13)	79.3	16380912, 17634449

Alias denotes the abbreviated name of study used in figures and analyses; ACS = acute coronary syndrome, CAD = coronary artery disease; PubMed IDs are provided for individual study descriptions; mean (standard deviation) with proportions (%) are provided unless otherwise stated.

Circulation: Genomic and Precision Medicine

Table 2: Participant characteristics of each study contributing to GENIUS-CHD.

ALIAS	BMI, kg/m2 (SD)	Systolic BP (SD)	Diastolic BP (SD)	Diabetes (%)	Current Smoking (%)	Total cholesterol (SD), mmol/L	LDL-C (SD), mmol/L	HDL-C (SD), mmol/L	Creatinine (SD)	Statin use (%)	Prior Revasc (%)	Prior MI (%)
4C	30.2 (5.7)	133.8 (23)	77.9 (12.2)	21.8	19.1	4.64 (1.10)	NA	1.309 (0.42)	98.7 (81)	24.7	20.6	14.1
AGNES	26.6 (3.9)	NA	NA	7.9	61.0	5.26 (1.04)	3.25 (1.01)	1.198 (0.45)	NA	10.0	0.0	0.0
ANGES	28.1 (4.4)	NA	NA	30.8	14.7	4.71 (0.84)	2.68 (0.77)	1.166 (0.33)	83.0 (37)	69.4	42.4	24.7
ATVB	26.8 (4.0)	132.4 (21)	83.5 (13.5)	8.2	79.5	5.83 (1.39)	NA	1.080 (0.33)	NA	55.4	NA	NA
CABGenomics	29.8 (5.6)	NA	NA	9.0	10.3	4.32 (0.94)	2.13 (0.85)	1.085 (0.35)	NA	74.1	NA	37.0
CARDIOLINES	26.9 (3.8)	134.4 (23)	84.34 (14.6)	NA	0.6	5.43 (1.1)	3.84 (1.0)	1.16 (0.3)	73.09 (15)	NA	NA	NA
CDCS	27.3 (4.7)	129.1 (22)	74.6 (11.7)	15.2	5.8	5.01 (1.09)	2.95 (1.03)	1.175 (0.34)	100.8 (41)	46.0	26.5	30.4
COGEN	NA	NA	NA	16.7	26.2	NA	NA	NA	NA	NA	NA	NA
COROGENE	27.6 (4.8)	NA	NA	18.2	34.4	4.58 (0.99)	2.43 (0.88)	1.250 (0.37)	84.0 (46)	5.2	NA	NA
CTMM	27.6 (4.4)	135.5 (19)	77.4 (11.2)	21.0	20.9	4.54 (1.06)	2.59 (0.98)	1.135 (0.32)	86.2 (40)	NA	NA	30.3
CURE	27.7 (4.5)	135.1 (22)	77.1 (13.6)	20.9	23.0	NA	NA	NA	93.1 (35)	NA	14.8	31.7
EGCUT	29.0 (5.2)	135.7 (18)	80.4 (10.6)	18.9	19.8	5.70 (1.17)	3.84 (1.08)	1.340 (0.35)	NA	27.7	15.4	35.3
EMORY	29.8 (6.7)	137.0 (22)	75.0 (15.0)	34.2	7.8	4.49 (1.04)	2.42 (0.93)	1.090 (0.34)	100.2 (56)	74.2	59.6	26.8
ERICO	27.0 (5.1)	134.8 (32)	99.4 (38.0)	39.4	31.2	NA	NA	NA	NA	23.8	11.7	26.2
FASTMI2005	27.2 (4.8)	139.9 (28)	80.0 (17.0)	35.9	29.1	5.03 (1.22)	3.03 (1.07)	1.239 (0.43)	103.4 (62)	74.1	NA	18.2
FINCAVAS	27.8 (4.3)	140.2 (22)	82.2 (10.6)	18.4	24.3	4.70 (0.90)	2.62 (0.80)	1.300 (0.39)	90.8 (70)	57.3	32.6	39.0
FRISCII	26.8 (3.9)	143.4 (23)	82.0 (10.6)	12.8	27.0	5.81 (1.12)	3.72 (0.99)	1.151 (0.36)	90.6 (19)	12.3	12.1	27.2
GENDEMIP	28.6 (4.7)	137.1 (21)	84.0 (10.8)	19.0	61.0	5.42 (1.16)	3.58 (1.09)	1.183 (0.33)	NA	16.7	30.2	40.8
GENEBANK	29.4 (5.4)	132.7 (21)	75.0 (12.0)	11.8	16.8	4.38 (0.93)	2.51 (0.82)	0.903 (0.26)	NA	71.8	65.3	56.1
GENESIS-PRAXY	29.5 (6.5)	139.5 (27)	86.2 (17.2)	13.9	44.2	4.87 (1.19)	2.89 (1.13)	0.966 (0.30)	75.9 (20)	92.9	11.4	11.5
GENOCOR	NA	129.5 (20)	75.4 (11.1)	13.3	64.4	4.82 (0.92)	3.10 (0.83)	1.082 (0.28)	94.8 (27)	72.1	13.7	63.2
GEVAMI	27.2 (4.3)	124.8 (18)	73.2 (11.1)	8.9	52.4	NA	NA	NA	NA	13.4	0.0	0.0
GoDARTSincident	29.8 (5.6)	126.7 (17)	NA	70.9	NA	4.57 (1.02)	2.43 (0.91)	1.277 (0.41)	107.0 (65)	49.6	0.2	1.2
GoDARTSprevalent	30.2 (5.4)	136.0 (20)	NA	75.8	14.5	4.37 (0.84)	2.04 (0.74)	1.320 (0.38)	101.0 (34)	66.3	30.2	46.8
GRACE_B	27.0 (4.3)	138.3 (25)	78.7 (14.6)	81.1	49.3	5.19 (1.20)	3.06 (1.09)	1.343 (0.98)	102.6 (63)	79.4	NA	80.5
GRACE_UK	27.9 (5.0)	137.9 (27)	76.4 (16.5)	13.9	69.2	5.20 (1.27)	3.07 (1.14)	1.204 (0.49)	101.5 (38)	14.5	20.2	30.0
IDEAL	27.3 (3.8)	136.9 (20)	80.4 (10.2)	11.9	20.7	5.09 (1.00)	3.14 (0.90)	1.192 (0.31)	100.6 (17)	75.5	40.9	100.0
INTERMOUNTAIN	29.5 (6.1)	141.8 (24)	81.1 (13.3)	20.3	10.2	4.91 (1.12)	2.76 (0.94)	1.048 (0.35)	99.6 (67)	38.7	NA	6.6
INVEST	29.4 (5.6)	148.4 (18)	82.4 (10.5)	24.3	13.3	NA	NA	NA	NA	52.7	48.1	23.3
JUMC	26.3 (4.5)	148.2 (25)	80.3 (12.4)	36.1	27.5	4.97 (1.08)	3.11 (1.14)	1.232 (0.37)	91.3 (42)	87.5	49.8	39.9
KAROLA	26.9 (3.3)	120.0 (16)	73.1 (9.1)	18.6	31.8	4.44 (0.84)	2.61 (0.76)	1.030 (0.28)	82.7 (28)	77.0	42.8	22.4
LIFE-Heart	28.9 (4.7)	139.0 (22)	80.0 (12.9)	33.9	27.8	5.16 (1.19)	3.12 (1.05)	1.227 (0.35)	88.8 (34)	45.8	NA	13.3
LURIC	27.5 (4.0)	142.2 (24)	81.0 (11.5)	44.1	24.6	4.94 (0.99)	2.98 (0.89)	0.965 (0.26)	88.7 (38)	58.9	48.3	57.8
MDCS	25.8 (4.0)	141.1 (20)	85.6 (10.0)	4.4	26.6	6.17 (1.1)	4.16 (1.0)	1.38 (0.4)	84.76 (16)	0.03	0.00	0.00
NE_POLAND	24.8 (3.8)	138.7 (27)	88.1 (15.6)	22.3	48.5	5.12 (1.04)	3.31 (0.97)	1.126 (0.34)	92.0 (36)	81.2	1.7	11.2
NEAPOLIS	28.0 (4.2)	129.4 (14)	75.7 (7.7)	42.7	26.9	4.49 (1.03)	2.45 (0.99)	1.233 (0.66)	101.0 (68)	82.6	41.9	40.9
OHGS	28.5 (4.9)	132.2 (19)	72.1 (11.3)	5.5	19.3	5.57 (1.05)	3.46 (0.88)	1.222 (0.34)	89.1 (21)	91.6	27.8	23.3
PERGENE	27.5 (3.5)	136.9 (15)	81.8 (8.1)	12.7	14.7	5.41 (1.04)	NA	NA	86.5 (26)	55.3	54.6	65.4
PLATO	28.2 (4.5)	135.6 (22)	79.5 (12.9)	22.8	35.2	5.40 (1.23)	3.27 (1.11)	1.279 (0.35)	85.6 (26)	79.7	15.1	20.6
PMI	26.5 (3.8)	116.5 (16)	66.5 (9.6)	12.5	28.0	5.97 (1.19)	3.98 (1.07)	NA	88.0 (28)	44.6	NA	18.4
POPular	27.2 (4.1)	144.9 (22)	81.4 (12.1)	19.0	27.6	4.56 (0.94)	2.73 (1.15)	1.260 (0.32)	92.7 (27)	80.7	32.9	43.6
POPular Genetics	NA	NA	NA	NA	NA	NA	NA	NA	NA	NA	NA	NA

ALIAS	BMI, kg/m2 (SD)	Systolic BP (SD)	Diastolic BP (SD)	Diabetes (%)	Current Smoking (%)	Total cholesterol (SD), mmol/L	LDL-C (SD), mmol/L	HDL-C (SD), mmol/L	Creatinine (SD)	Statin use (%)	Prior Revasc (%)	Prior MI (%)
PROSPER	26.6 (3.9)	150.0 (22)	81.1 (11.4)	10.4	17.3	5.55 (0.84)	3.74 (0.74)	1.174 (0.31)	109.2 (23)	0.0	26.5	86.9
RISCA	27.2 (4.4)	NA	NA	19.8	30.4	NA	NA	NA	100.6 (29)	46.6	28.3	27.8
SHEEP	26.8 (4.0)	131.8 (21)	79.6 (10.3)	18.2	50.1	6.20 (1.16)	4.22 (1.01)	1.082 (0.31)	NA	0.0	0.0	0.0
SMART	27.4 (3.7)	137.0 (19)	80.1 (10.8)	17.1	24.2	4.66 (0.95)	2.64 (0.88)	1.231 (0.72)	92.3 (23)	77.5	100.0	44.5
STABILITY	29.9 (5.0)	131.7 (16)	79.1 (10.0)	38.4	21.4	NA	2.25 (0.85)	1.216 (0.32)	NA	97.3	74.6	58.6
THI	29.6 (5.6)	NA	NA	30.4	21.1	NA	NA	NA	NA	57.2	21.7	16.7
TNT	28.5 (4.5)	130.7 (17)	77.9 (9.4)	14.2	13.3	4.53 (0.61)	2.52 (0.45)	1.223 (0.28)	104.5 (17)	70.1	NA	58.2
TRIUMPH	29.6 (6.0)	117.7 (18)	68.1 (10.9)	29.1	37.4	NA	2.70 (1.02)	1.037 (0.33)	113.7 (81)	89.0	27.2	18.5
UCORBIO	27.2 (4.3)	NA	NA	21.4	23.1	4.80 (1.18)	2.64 (1.05)	1.205 (0.33)	92.0 (45)	63.9	NA	29.0
UCP	NA	153.4 (25)	87.1 (13.3)	NA	NA	5.66 (1.10)	3.36 (1.01)	1.244 (0.33)	94.7 (25)	27.0	NA	NA
UKB	29.4 (4.9)	139.1 (20)	78.7 (10.9)	22.2	75.9	NA	NA	NA	NA	82.9	59.6	36.7
VHS	26.8 (3.6)	NA	NA	18.4	69.1	5.51 (1.13)	3.69 (1.00)	1.175 (0.30)	96.7 (32)	46.4	17.6	59.4
VIVIT	27.4 (4.1)	137.4 (19)	80.6 (10.9)	31.0	19.4	5.36 (1.15)	3.33 (1.02)	1.348 (0.40)	89.9 (41)	49.9	20.6	30.4
WARSAW ACS	28.1 (4.7)	128.0 (23)	76.2 (13.2)	21.8	42.4	4.98 (1.06)	2.99 (1.02)	1.105 (0.33)	93.5 (44)	NA	NA	18.9
WTCC	27.6 (4.2)	143.6 (22)	84.3 (12.3)	11.7	12.8	5.31 (0.98)	3.12 (0.90)	1.198 (0.38)	NA	71.6	67.2	72.0

Data were collected through a federated analysis. Alias denotes the abbreviated name of study used in figures and analyses; BMI = body mass index; BP = blood pressure; LDL-C = low density lipoprotein cholesterol; HDL-C = high-density lipoprotein cholesterol; MI = myocardial infarction; mean (standard deviation) and proportions (%) are provided unless otherwise stated.

American Heart Association.
Circulation: Genomic and Precision Medicine

Figure Legends

Figure 1: Overview of the GENIUS-CHD consortium, illustrating inclusion criteria and governance structure. Following project approval by the steering committee, analyses scripts are prepared and distributed to all members, with sharing of summary level outputs before meta-analysis at the coordinating centres. CHD = coronary heart disease; QC = quality control. Further details can be found at www.genius-chd.org

Figure 2: Figure illustrating empirical power for detecting different effect sizes for biomarker variance and clinical events for both alpha 0.05 and 0.0001, by varying minor allele frequencies, for a conservative total N of 150,000 with an event rate of 10%. MAF = minor allele frequency; OR = odds ratio

American Heart
Circulation: Genomic and Precision Medicine

Figure 3: Meta analyses of the associations between age (per 5-year intervals) and different endpoints, adjusted for sex. Estimates are presented as hazard ratios (HRs) with 95% confidence intervals (95% CI). CHD = coronary heart disease; CVD = cardiovascular disease; MI = myocardial infarction.

Figure 4: Meta analyses of the associations between male sex and different endpoints, adjusted for age. Estimates are presented as hazard ratios (HRs) with 95% confidence intervals (95% CI). CHD = coronary heart disease; CVD = cardiovascular disease; MI = myocardial infarction.

Figure 5: Meta analyses for the associations between smoking at CHD indexing event compared to not smoking and for different endpoints, adjusted for age and sex. Estimates are presented as hazard ratios (HRs) with 95% confidence intervals (95% CI). CHD = coronary heart disease; CVD = cardiovascular disease; MI = myocardial infarction.

American Heart Association.
Circulation: Genomic and Precision Medicine

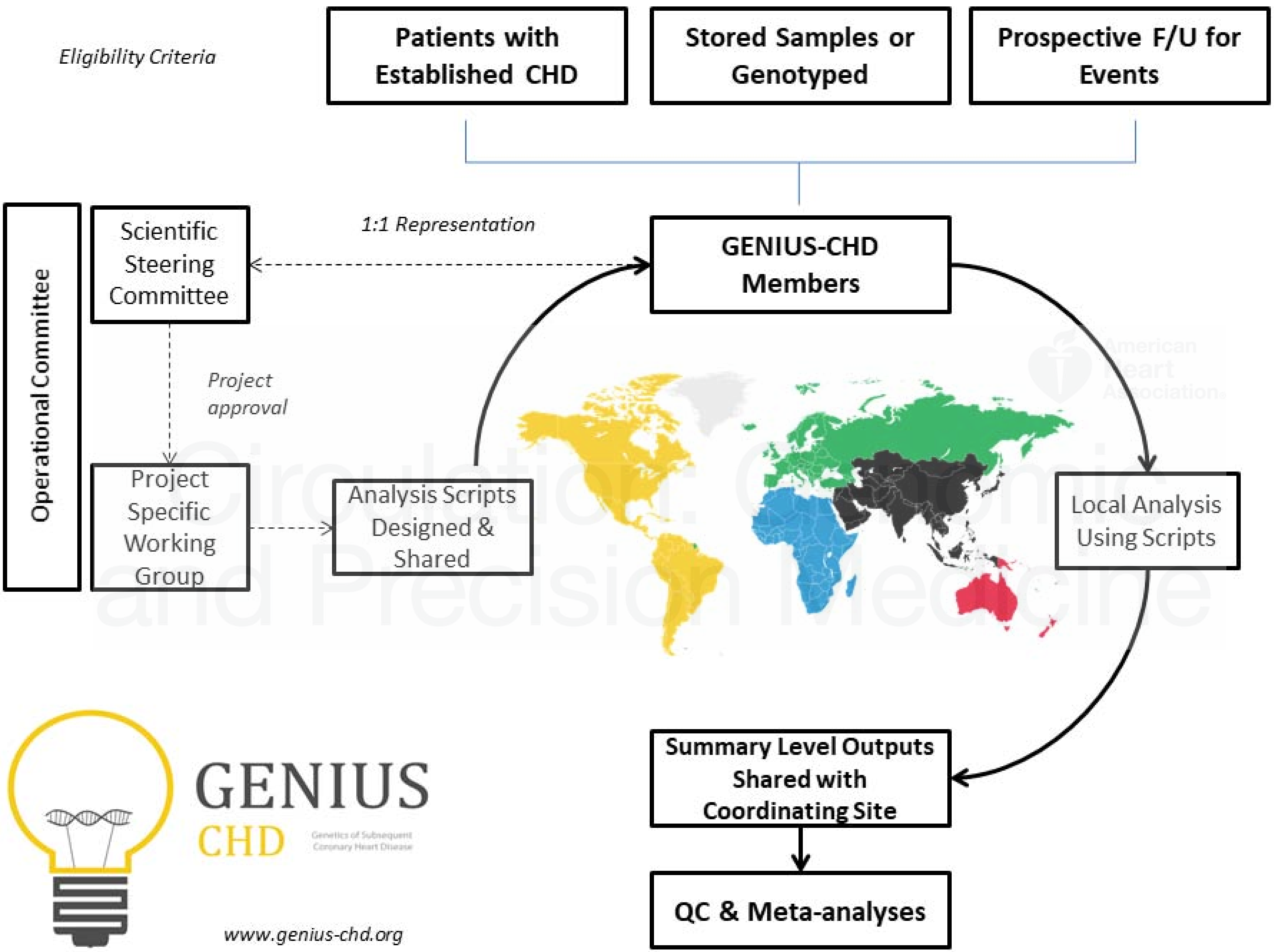

Circulation: Genomic and Precision Medicine
American Heart Association

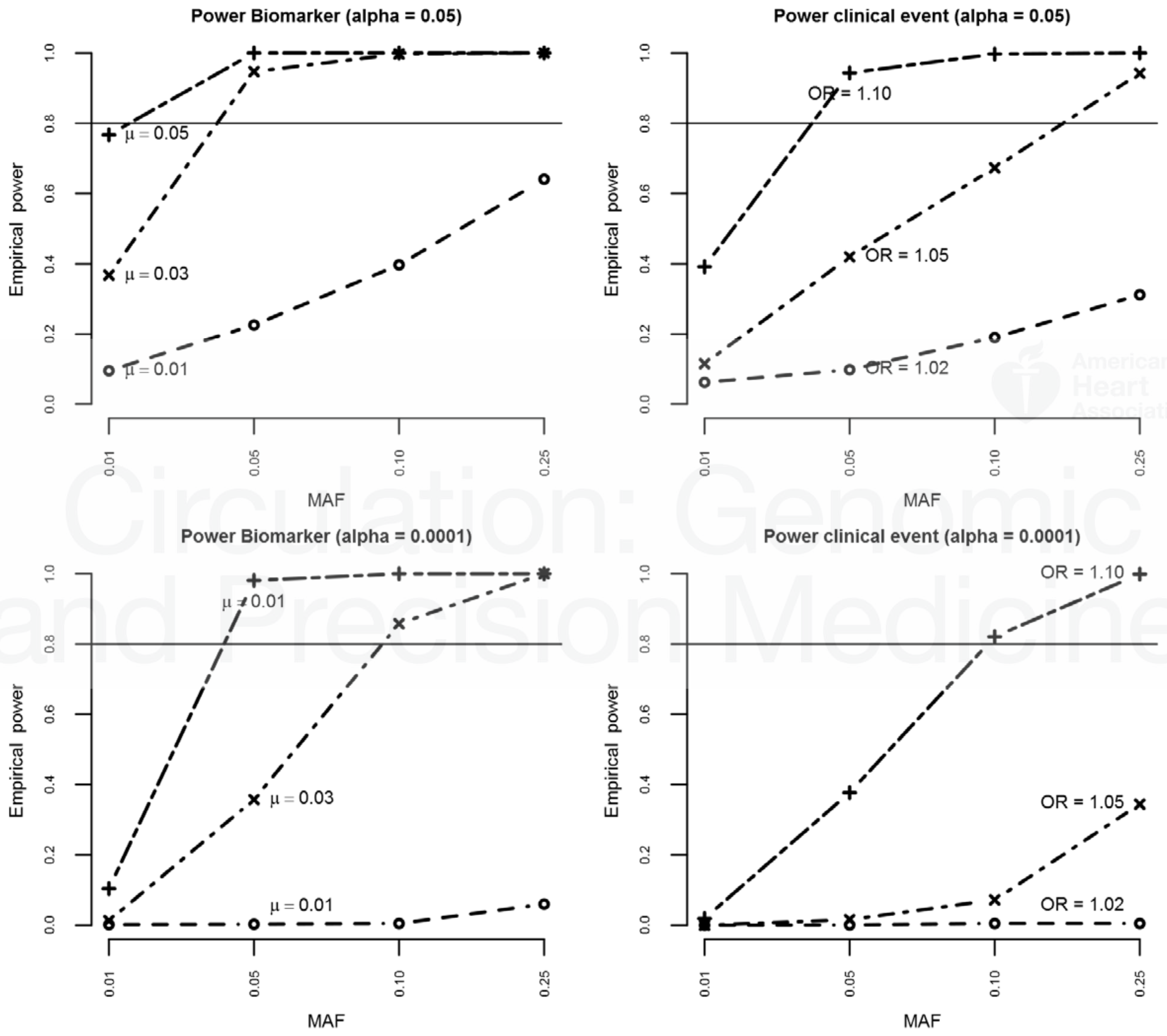

Circulation: Genomic and Precision Medicine
American Heart Association

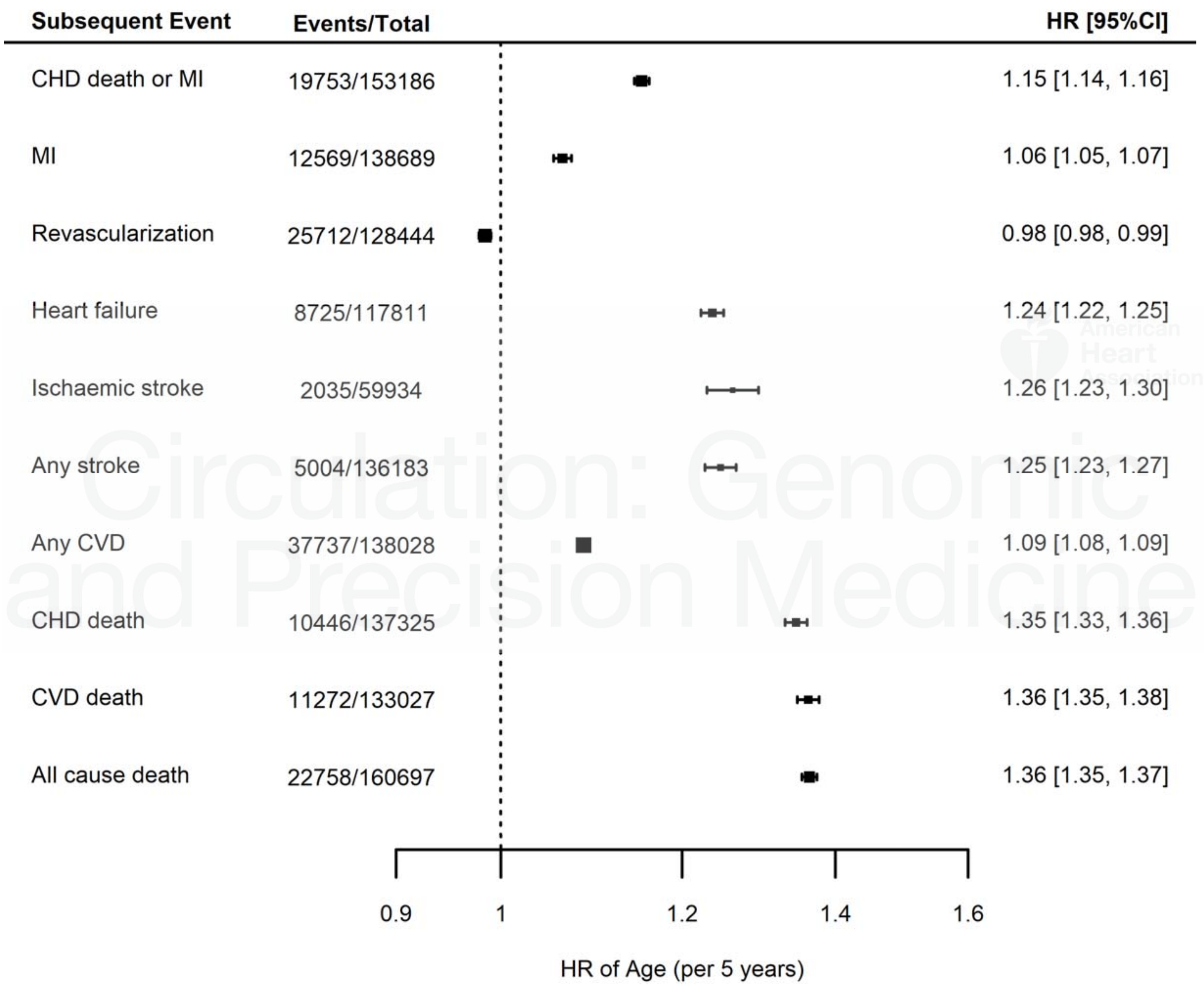

Circulation: Genomic and Precision Medicine
American Heart Association.

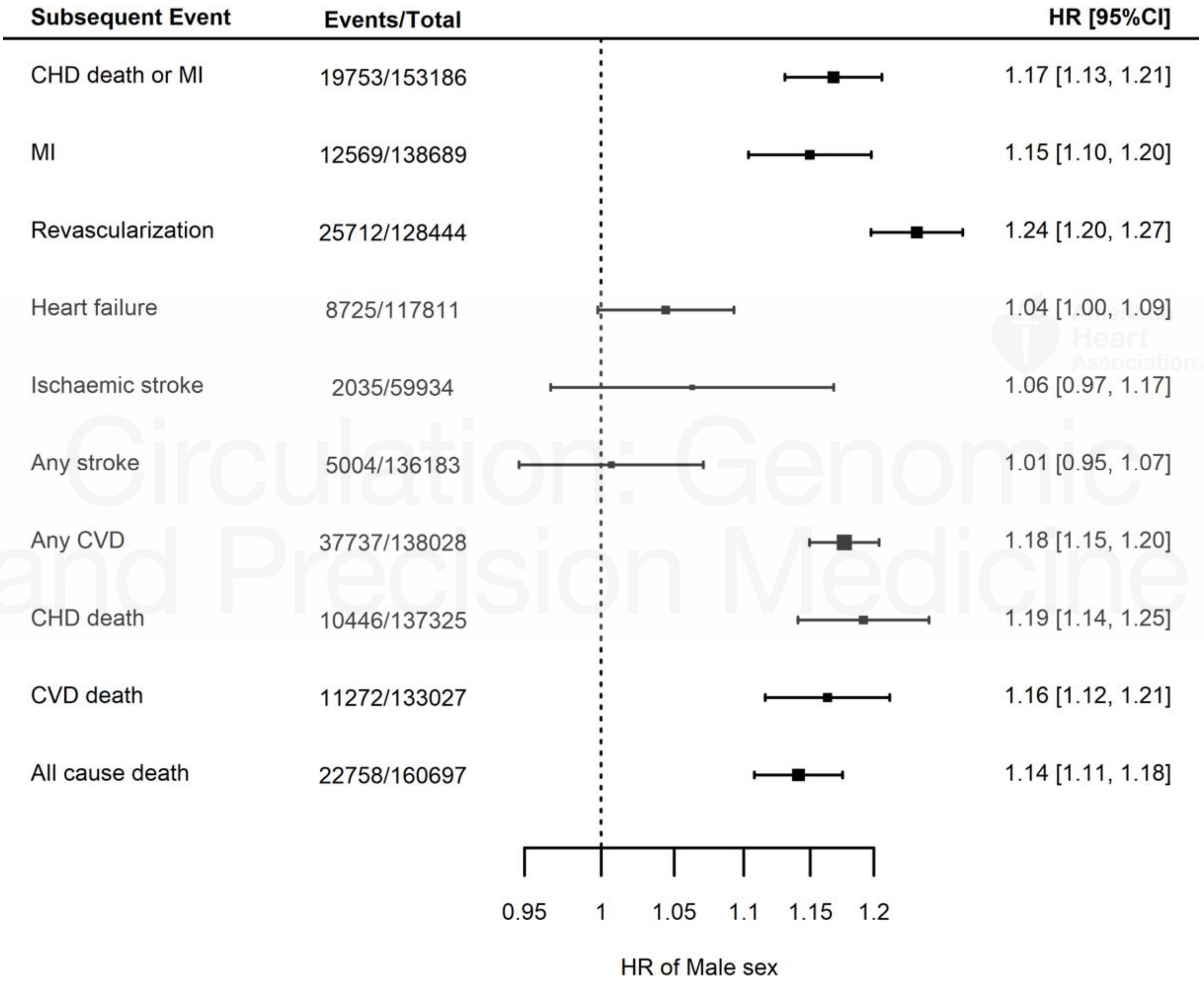

Circulation: Genomic and Precision Medicine

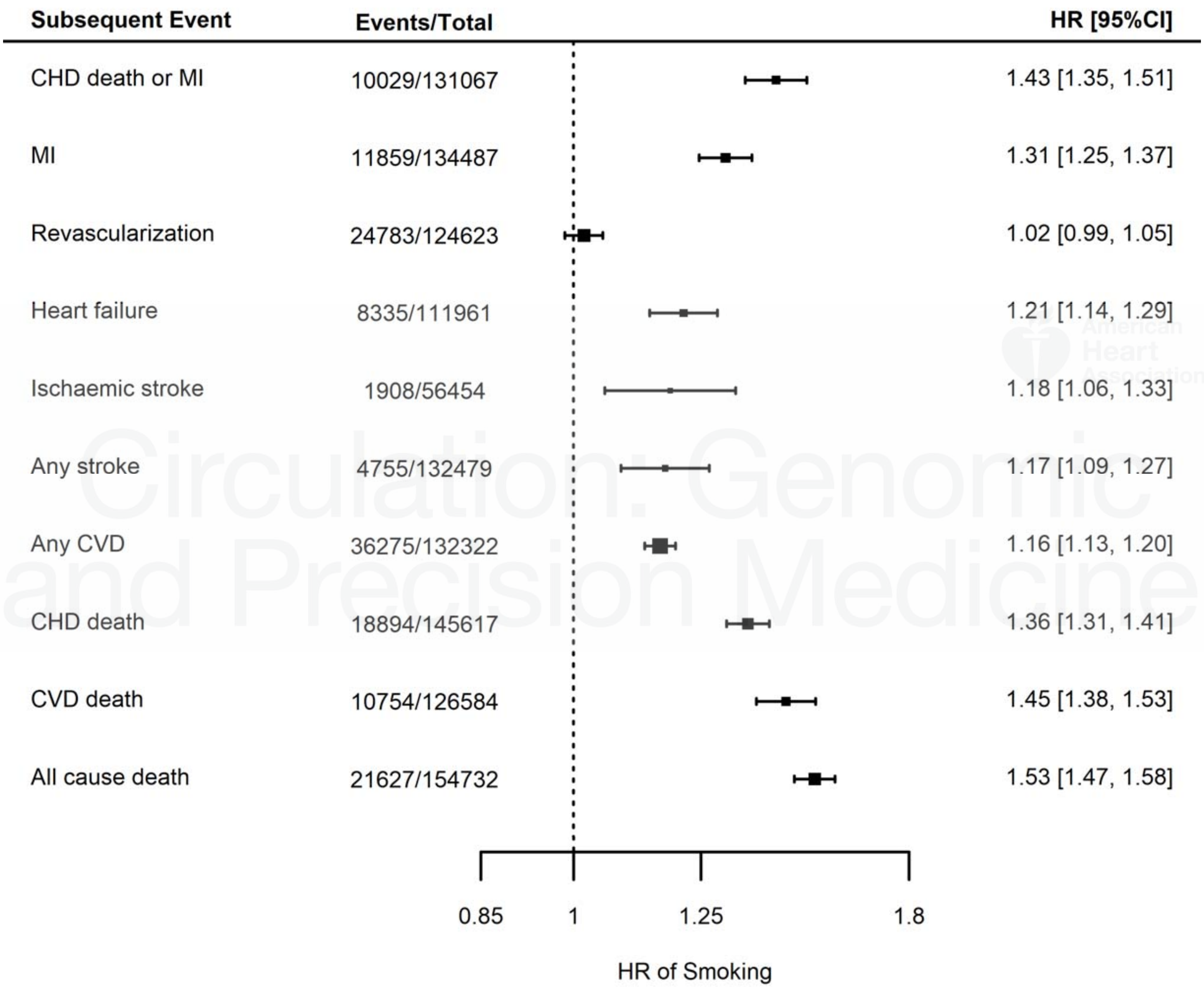

Circulation: Genomic and Precision Medicine
American Heart Association
